# Application of two-dimensional difference gel electrophoresis to identify protein changes between center, margin, and adjacent non-tumor tissues obtained from non-small-cell lung cancer with adenocarcinoma or squamous cell carcinoma subtype

**DOI:** 10.1371/journal.pone.0268073

**Published:** 2022-05-05

**Authors:** Andrzej Ciereszko, Mariola A. Dietrich, Mariola Słowińska, Joanna Nynca, Michał Ciborowski, Monika M. Kaczmarek, Kamil Myszczyński, Joanna Kiśluk, Anna Majewska, Anna Michalska-Falkowska, Natalia Kodzik, Joanna Reszeć, Ewa Sierko, Jacek Nikliński

**Affiliations:** 1 Department of Gametes and Embryo Biology, Institute of Animal Reproduction and Food Research, Polish Academy of Sciences, Olsztyn, Poland; 2 Metabolomics Laboratory, Clinical Research Centre, Medical University of Bialystok, Bialystok, Poland; 3 Molecular Biology Laboratory, Institute of Animal Reproduction and Food Research Polish Academy of Sciences, Olsztyn, Poland; 4 Department of Clinical Molecular Biology, Medical University of Bialystok, Bialystok, Poland; 5 Department of Medical Pathomorphology, Medical University of Bialystok, Bialystok, Poland; 6 Department of Oncology, Medical University of Bialystok, Bialystok, Poland; CHA University, REPUBLIC OF KOREA

## Abstract

Lung cancer is responsible for the most cancer-related mortality worldwide and the mechanism of its development is poorly understood. Proteomics has become a powerful tool offering vital knowledge related to cancer development. Using a two-dimensional difference gel electrophoresis (2D-DIGE) approach, we sought to compare tissue samples from non-small-cell lung cancer (NSCLC) patients taken from the tumor center and tumor margin. Two subtypes of NSCLC, adenocarcinoma (ADC) and squamous cell carcinoma (SCC) were compared. Data are available via ProteomeXchange with identifier PXD032736 and PXD032962 for ADC and SCC, respectively. For ADC proteins, 26 significant canonical pathways were identified, including Rho signaling pathways, a semaphorin neuronal repulsive signaling pathway, and epithelial adherens junction signaling. For SCC proteins, nine significant canonical pathways were identified, including hypoxia-inducible factor-1α signaling, thyroid hormone biosynthesis, and phagosome maturation. Proteins differentiating the tumor center and tumor margin were linked to cancer invasion and progression, including cell migration, adhesion and invasion, cytoskeletal structure, protein folding, anaerobic metabolism, tumor angiogenesis, EMC transition, epithelial adherens junctions, and inflammatory responses. In conclusion, we identified several proteins that are important for the better characterization of tumor development and molecular specificity of both lung cancer subtypes. We also identified proteins that may be important as biomarkers and/or targets for anticancer therapy.

## Introduction

Cancer is the leading cause of human mortality. Among cancers, lung cancer is the most common in the world and responsible for the most cancer-related mortality worldwide [1]. Non-small-cell lung cancer (NSCLC), the most common type of lung cancer (85% of cases), can be divided into three main subgroups: adenocarcinoma (ADC, 30–50% of cases), squamous cell carcinoma (SCC, ∼30%), and large cell carcinoma (LCC, ∼10%) [2]. The prevention of lung cancer is still a great challenge. The symptoms of lung cancer are usually very difficult to recognize until the disease has reached an advanced, non-curable state. Late diagnosis is a significant factor contributing to the poor prognosis for lung cancer [1]. It is estimated that only 16% of patients survive for five or more years after diagnosis. For this reason, the development of biomarkers for effective prognosis is of utmost importance [3].

Biomarkers are biological compounds that can be used to distinguish pathology from normal status. At the molecular level, proteins represent the most important functional unit that is directly responsible for a phenotype. For this reason, almost all the Food and Drug Administration approved cancer biomarkers are proteins [4]. Unfortunately, the number of potential protein markers is very limited and, at present, restricted to a few proteins, such as cytokeratin 19 fragments (CYFRA 21–1), carcinoembryonic antigen, SCC antigen, neuron-specific enolase, progastrin-releasing peptide, and epidermal growth factor receptor [1]. The most common approach for discovering new markers is to analyze proteins overexpressed in lung cancer tissue and use this knowledge to select prospective cancer markers [5, 6].

The proteome is defined as the total proteins expressed by a genome, including their numerous different forms (proteoforms) produced by post-translational modifications (PTMs). Proteomics has become a very powerful tool for studies of proteomes in relation to cancer, and among the methodical approaches, those based on two-dimensional electrophoresis (2DE) have proven especially useful [7]. Two-dimensional difference gel electrophoresis (2D-DIGE) is variation of 2DE and includes steps such as pre-electrophoretic fluorescent labelling, electrophoretic separation in the first dimension using electrofocusing, electrophoretic separation in the second dimension using SDS-PAGE, protein spot detection using the scanning of gels at three wavelengths to detect different fluorophores, and intragel comparisons with image analysis programs to evaluate differentially expressed proteins [8]. 2D-DIGE has become the favored method for the proteomic analysis of lung cancer due to its high reproducibility, high sensitivity, comprehensiveness, and high throughput [9, 10]. We recently successfully used 2D-DIGE for the identification of protein changes in the blood plasma of lung cancer patients subjected to chemotherapy [11]. Due to the extreme complexity of the blood proteome, spanning a concentration range of at least 10 orders of magnitude [12, 13], we were mainly able to detect blood proteins that change in lung cancer patients. However, to identify cancer-related proteins, the analysis of cancer tissues is preferred. It should be underlined that liquid chromatography mass spectrometry (LC-MS)-based proteomics is being introduced to cancer research as well and now is extensively used for large-scale protein analysis [14–26].

Several studies utilizing the 2D-DIGE approach for the identification of potential lung cancer biomarkers have been published to date, leading to the identification of several potential protein markers [21, 27–30]. The results of these studies have identified numerous protein characteristics for particular cancer types and their progression [10]. However, the biomarkers often differ among studies, which makes it difficult to recommend them as diagnostic targets. It is not surprising due to tumor heterogeneity and difference in analytical procedures used. Therefore the meta-analysis of such data is invaluable. To our knowledge, the majority of these studies have focused on comparing cancer tissue with adjacent normal tissue. Such an approach presents limitations due to intratumoral heterogeneity, which can result in differences when identifying biomarkers are based on the results of single biopsies [31–33]. It is especially important from the point of view of the occurrence of specific biochemical characteristics at the invasive front of lung cancer [34–36]. Therefore, comparative studies of the center and margin of the tumor could provide important insights into tumor characteristics and development.

In our study, using the 2D-DIGE approach, we sought to compare the proteomes of the tumor center and margin (representing tumor progression) of NSCLC. We hypothesized that these two tumor areas would differ in their proteomic profiles, reflecting different metabolic and structural characteristics. We performed our studies on two subtypes of NSCLC, ADC and SCC. For both subtypes, the proteomes of the tumor center and margin were also compared to the proteome of adjacent normal lung tissues.

## Results

### 2D-DIGE analysis of differentially expressed proteins between center and margin of ADC tumor

**Fig 1:**
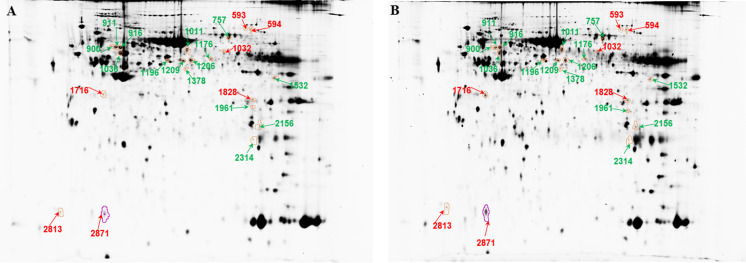
Representative 2D-DIGE profiling of differentially expressed proteins in the margin and center of ADC tumor. (A) single-channel image of proteins from tumor margin, (B) single-channel image of proteins from tumor center. Red numbers indicate proteins that are more abundant in center and green numbers indicate proteins with more abundance in margin. Twenty-two spots for ADC (numbers correlate with descriptions in S1 Table) with significantly different abundance between the margin and center of tumor are shown (p < 0.05).

We found 22 differentially abundant spots representing 17 proteins (Fig 1A and 1B and S1 Table). The tumor center was characterized by a higher abundance of LMNA (two proteoforms), dihydropyriminidase-related protein (DPYSL3), tubulin beta (TUBB), myosin light chain 3 (MYL3), and galectin-1 (LGALS1). On the other hand, the tumor margin was characterized by a higher abundance of moesin isoform X1 (MSN), plastin (LCP1 (three proteoforms), leukotriene A4 hydrolase (LTA4H), mitochondrial heat shock protein 60 kDa (HSPD1), T-complex protein 1, isoform 2 (CCT2), mitochondrial aldehyde dehydrogenase 2 (ALDH2, two proteoforms), tryptophan-tRNA ligase (WARS1), sorting nexin-6 (SNX6), citrate synthase (CS), albumin (ALB), and proteasome alpha 2 subunit (PSMA1). Two proteoforms of carbonic anhydrase 1 (CA1) showed changes in the opposite direction: spot 1828 increased in abundance in the tumor center, while spot 1961 increased in the margin.

### 2D-DIGE analysis of differentially expressed proteins between center and margin of SCC tumor

**Fig 2:**
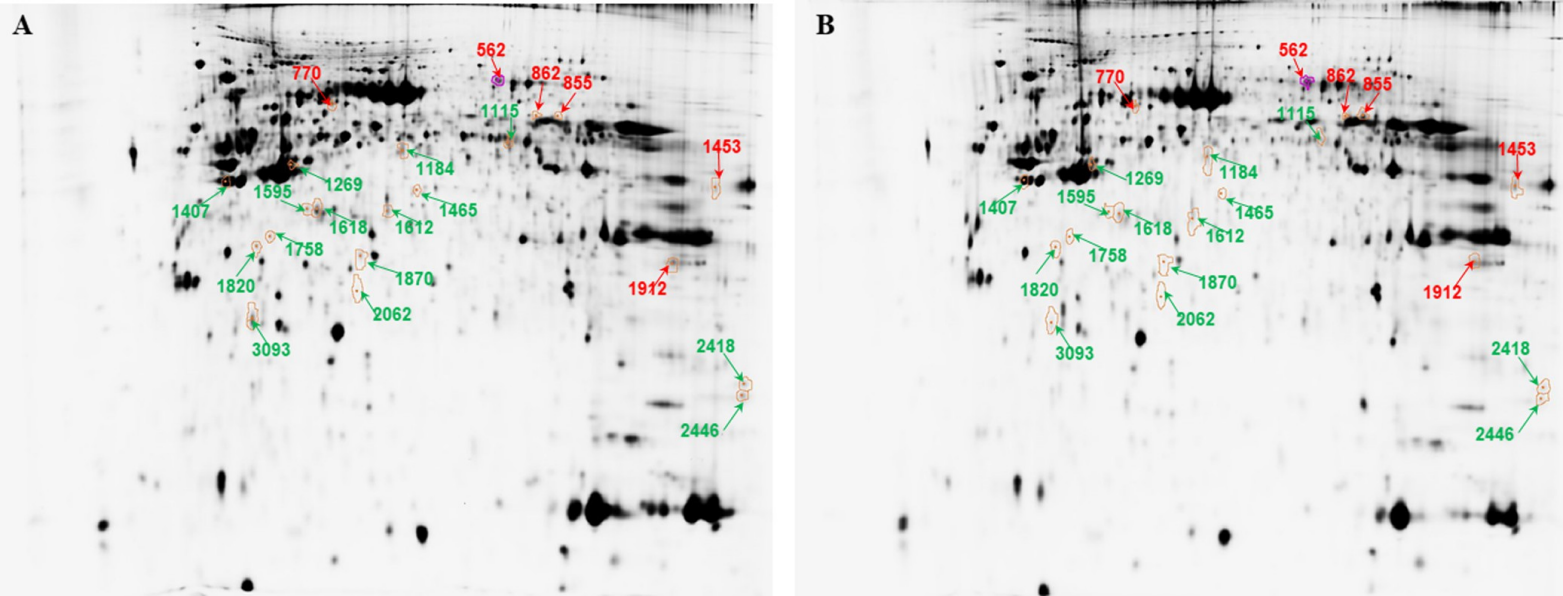
Representative 2D-DIGE profiling of differentially expressed proteins in the margin and center of SCC tumor. (A) single-channel image of proteins from tumor margin, (B) single-channel image of proteins from tumor center. Red numbers indicate proteins that are more abundant in center and green numbers indicate proteins with more abundance in margin. Twenty-one spots for SCC (numbers correlate with descriptions in S2 Table) with significantly different abundance between the margin and center of tumor are shown (p < 0.05).

We found 21 differentially expressed abundant spots representing 21 proteins (Fig 2A and 2B and S2 Table). The tumor center was characterized by a higher abundance of transferrin (TF), endoplasmic reticulum oxidoreductase 1 (ERO1)-like protein (ERO1A), EH domain-containing protein 1 (EHD1), keratin 5 (KRT5), serpin H1 protein (SERPINH1), and lactate dehydrogenase A (LDHA). On the other hand, the tumor margin was characterized by a higher abundance of TATA binding protein (RUVBL1), PKM isoform d, HNRPF protein (HNRPF), KRT19, serpin B1 protein (SERPINB1), inorganic pyrophosphatase (PPA1), N-acetyl-D-glucosamine kinase isoform 1 (NAGK), F-actin-capping protein (CAPZA1), alpha SNAP (NAPA), microtubule-associated protein RP/EB (MAPRE1), cathepsin D (CTSD), peroxiredoxin 4 (PRDX4), cyclophilin B (PPIB, two proteoforms), and ARHGDIB.

### 2D-DIGE analysis of differentially expressed proteins between center and margin of ADC tumor compared to control tissue

We found 79 differentially abundant spots representing 68 proteins; 40, 20, and 19 spots differed between the control and center and margin, control and center, and control and margin, respectively (S1 Table). Six proteins (LMNA, LCP1, HSPD1, CS, CA1, and ALB) were previously identified as differentially expressed between the tumor center and margin (S1 Table).

### 2D-DIGE analysis of differentially expressed proteins between center and margin of SCC tumor compared to control tissue

We found 111 differentially expressed protein spots representing 95 proteins; 84, 7, and 20 spots differed between the control and center and margin, control and center, and control and margin, respectively (S2 Table). Nine proteins (ERO1A, SERPINH1, NAGK, MAPRE1, PPA1, CTSD, LDHA, PRDX4, and PPIB) were previously identified as differentially expressed between the tumor center and margin (S2 Table).

### Verification of 2D-DIGE results by Western blotting

**Fig 3:**
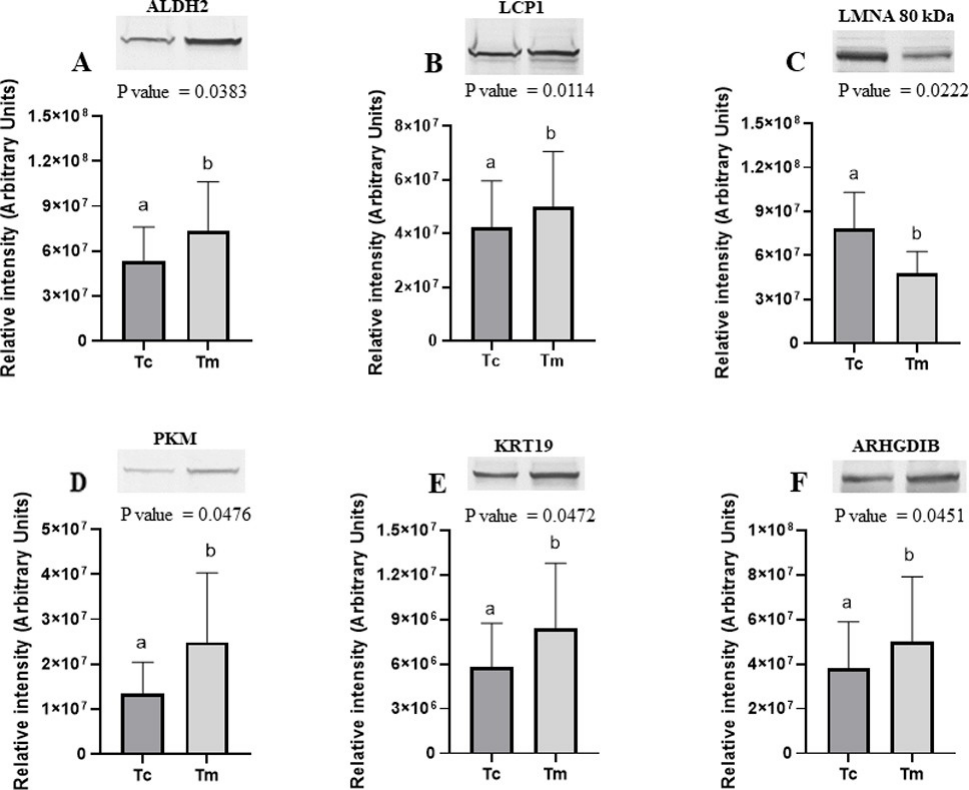
Immunoblotting verification of (A) mitochondrial aldehyde dehydrogenase 2 (ALDH2), (B) plastin (LCP1), (C) lamin 80 kDa (LMNA80 kDa) between center (Tc) and margin (Tm) of ADC tumor lung cancer and (D) pyruvate kinase PKM (PKM), (E) 40-kDa keratin (KRT19) and (F) rho GDP-dissociation inhibitor (ARHGDIB) between Tc and Tm of SCC tumor lung cancer. Results are expressed as means ± SD. Representative blots for one patient are shown. Asterisks indicate significant differences between Tc and Tm of tumor lung cancer (p < 0.05).

To verify the 2D-DIGE results, six proteins differing in abundance between the margin and center of ADC (ALDH2, LCP1, and LMNA) and SCC (PKM, KRT19, and ARHGDIB) were selected for further analysis using 1D Western blotting (6 biological replicates in each group). As presented in Fig 3, the changes in the abundance of the selected proteins were consistent with those obtained in the 2D-DIGE analysis. In ADC, the abundance of ALDH2 and LCP1 increased in the margin by 1.4- and 1.2-fold, respectively, while LMNA 80 kDa increased in the center by 1.6-fold (Fig 3A–3C). In SCC, the protein level of PKM and KRT19 increased in the margin by 1.8- and 1.4-fold, respectively, while ARHGDIB increased in the center by 1.4-fold (Fig 3D–3F).

### Ingenuity pathway analysis (IPA)

Complete sets of all the IPA results are presented in S1 and S2 Materials.

#### Canonical pathways

*ADC*. The top five canonical pathways are shown in Table 1. The margin vs. center comparison was characterized by two Rho signaling pathways, the semaphorin neuronal repulsive signaling pathway, epithelial adherens junction signaling, and phenylethylamine degradation. On the other hand, center vs. control and margin vs. control were characterized by three common pathways, acute phase response signaling, glycolysis I, and iron homeostasis signaling pathways. Moreover, the center vs. control comparison was distinguished by phagosome maturation and the remodeling of epithelial adherens junctions, and the margin vs. control comparison was distinguished by aldosterone signaling in epithelial cells and glucocorticoid receptor signaling.

**Table 1:** Top canonical pathways of differentially expressed proteins (DEPs) in ADC tumor.

Top Canonical Pathways	p-value	No of molecules	Proteins
**DEPs between Center and Margin**			
RhoA Signaling	3.35E-03	2	MSN, MYL3
Semaphorin Neuronal Repulsive Signaling Pathway	3.73E-03	2	DPYSL3, MYL3
Epithelial Adherens Junction Signaling	5.03E-03	2	MYL3, TUBB
RhoGDI Signaling	7.25E-03	2	MSN, MYL3
Phenylethylamine Degradation I	7.62E-03	1	ALDH2
**DEPs between Center and Control**			
Acute Phase Response Signaling	2.25E-05	5	ALB, APOA1, HNRNPK, HP, SERPINA1
Glycolysis I	6.78E-05	3	ENO1, GAPDH, PKM
Iron homeostasis signaling pathway	1.91E-04	4	HBA1/HBA2, HBB, HBD, HP
Phagosome Maturation	2.21E-04	4	CALR, PRDX2, PRDX5, TUBB
Remodeling of Epithelial Adherens Junctions	2.86E-04	3	ACTB, NME1, TUBB
**DEPs between Margin and Control**			
Acute Phase Response Signaling	2.25E-05	5	ALB, FGB, HNRNPK, HP, SERPINA1
Glycolysis I	6.78E-05	3	ENO1, GAPDH, PKM
Iron homeostasis signaling pathway	1.91E-04	4	HBA1/HBA2, HBB, HBD, HP
Aldosterone Signaling in Epithelial Cells	2.80E-04	4	AHCY, HSP90AB1, HSPA1A/HSPA1B, HSPD1
Glucocorticoid Receptor Signaling	4.44E-04	5	ACTB, HSP90AB1, HSPA1A/HSPA1B, KRT19, KRT34

*SCC*. The top five canonical pathways are shown in Table 2. The margin vs. center was characterized by hypoxia-inducible factor-1α (HIF-1α) signaling, thyroid hormone biosynthesis, phagosome maturation, pyruvate fermentation to lactate, and N-acetylglucosamine degradation II. On the other hand, center vs. control and margin vs. control were characterized by five common pathways: glucocorticoid receptor signaling, the unfolded protein response, aldosterone signaling in epithelial cells, glycolysis I, and gluconeogenesis I.

**Table 2:** Top canonical pathways of differentially expressed proteins (DEPs) in SCC tumor.

Top Canonical Pathways	p-value	No of molecules	Proteins
**DEPs between Center and Margin**			
HIF1α Signaling	6.47E-04	3	LDHA, PKM, TF
Thyroid Hormone Biosynthesis	5.71E-03	1	CTSD
Phagosome Maturation	7.30E-03	2	CTSD, NAPA
Pyruvate Fermentation to Lactate	8.15E-03	1	LDHA
N-acetylglucosamine Degradation II	9.77E-03	1	NAGK
**DEPs between Center and Control**			
Glucocorticoid Receptor Signaling	3.52E-11	12	ACTB, HSP90AA1, HSP90AB1, HSP90B1, HSPA1A/HSPA1B, HSPA5, HSPA8, KRT10, KRT17, KRT5, KRT6B, KRT8
Unfolded protein response	4.97E-09	6	CALR, HSP90B1, HSPA1A/HSPA1B, HSPA5, HSPA8, P4HB
Aldosterone Signaling in Epithelial Cells	7.90E-09	8	HSP90AA1, HSP90AB1, HSP90B1, HSPA1A/HSPA1B, HSPA5, HSPA8, HSPD1, PDIA3
Glycolysis I	5.94E-08	5	ALDOA, ENO1, GAPDH, PGAM1, PGK1
Gluconeogenesis I	1.06 E-07	5	ALDOA, ENO1, GAPDH, PGAM1, PGK1
**DEPs between Margin and Control**			
Glucocorticoid Receptor Signaling	1.98E-10	12	ACTB, HSP90AA1, HSP90AB1, HSP90B1, HSPA1A/HSPA1B, HSPA5, HSPA8, KRT10, KRT17, KRT5, KRT6B, KRT8
Aldosterone Signaling in Epithelial Cells	1.09E-09	9	HSP90AA1, HSP90AB1, HSP90B1, HSPA1A/HSPA1B, HSPA5, HSPA8, HSPB1, HSPD1, PDIA3
Unfolded protein response	1.17E-08	6	CALR, HSP90B1, HSPA1A/HSPA1B, HSPA5, HSPA8, P4HB
Glycolysis I	1.21E-07	5	ALDOA, ENO1, GAPDH, PGAM1, PGK1
Gluconeogenesis I	2.15E-07	5	ALDOA, ENO1, GAPDH, PGAM1, PGK1

#### Disease and disorder categories

*ADC*. The top five disease and disorder categories are shown in Table 3. The margin vs. center comparison was characterized by immunological, hematological, and inflammatory disease; inflammatory responses and organismal injury and abnormalities were indicated as well. The latter category for all the comparisons comprised several cancers, including lung cancer annotations (for details, see S1 Material). On the other hand, center vs. control and margin vs. control were characterized by the same three categories: organismal injury and abnormalities, gastrointestinal disease, and inflammatory responses. Additionally, the categories endocrine system disorders and immunological disease were indicated for center vs. control and cancer and hepatic system disease for margin vs. control comparison.

**Table 3:** Top disease and disorder of differentially expressed proteins (DEPs) in ADC tumor.

Disease and Disorder	p-value	No of molecules	Proteins
**DEPs between Center and Margin**			
Immunological Disease	1.79E-02–2.15E-06	10	ALB, ALDH2, CA1, HSPD1, LCP1, LGALS1, LTA4H, MSN, TUBB, WARS1
Hematological Disease	1.59E-02–3.88E-06	4	ALB, ALDH2, CA1, TUBB
Inflammatory Disease	1.79E-02–3.88E-06	9	ALB, ALDH2, CA1, HSPD1, LCP1, LGALS1, LTA4H, TUBB, WARS1
Inflammatory Response	1.93E-02–3.88E-06	10	ALB, ALDH2, CA1, CCT2, HSPD1, LCP1, LGALS1, LTA4H, MSN, TUBB
Organismal Injury and Abnormalities	1.99E-02–3.88E-06	17	ALB, ALDH2, CA1, CCT2, CS, DPYSL3, HSPD1, LCP1, LGALS1, LMNA, LTA4H, MSN, MYL3, PSMA1, SNX6, TUBB, WARS1
**DEPs between Center and Control**			
Endocrine System Disorders	1.65E-03–4.84E-15	43	AKR1B1, ALB, ANXA2, ANXA3, ANXA4, APOA1, ATIC, CA1, CALR, EEF1A1, EEF1B2, EEF2, ENO1, G6PD, GAPDH, HBA1/HBA2, HBB, HBD, HDGF, HNRNPK, HP, HSP90AB1, IDH1, ILF2, LCP1, LPP, MZB1, PDIA4, PFN1, PKM, PPA1, PPIA, PRDX2, PRDX5, PSMD7, RBBP4, RPSA, S100A4, SERPINA1, SND1, TUBB, UGDH, YWHAZ
Organismal Injury and Abnormalities	1.88E-03–4.84E-15	46	ACTB, AKR1B1, ALB, ANXA2, ANXA3, ANXA4, APOA1, ATIC, CA1, CALR, EEF1A1, EEF1B2, EEF1G, EEF2, ENO1, G6PD, GAPDH, HBA1/HBA2, HBB, HBD, HDGF, HNRNPK, HP, HSP90AB1, IDH1, ILF2, LCP1, LPP, MZB1, NME1, PDIA4, PFN1, PKM, PPA1, PPIA, PRDX2, PRDX5, PSMD7, RBBP4, RPSA, S100A4, SERPINA1, SND1, TUBB, UGDH, YWHAZ
Inflammatory Response	1.65E-03–2.95E-13	33	ACTB, ALB, ANXA2, ANXA3, ANXA4, APOA1, ATIC, CA1, CALR, EEF1A1, EEF1G, EEF2, ENO1, GAPDH, HBA1/HBA2, HBB, HP, HSP90AB1, IDH1, ILF2, LCP1, MZB1, PFN1, PKM, PPIA, PRDX2, PRDX5, PSMD7, RPSA, S100A4, SERPINA1, SND1, TUBB
Immunological Disease	1.88E-03–3.31E-12	36	ACTB, AKR1B1, ALB, ANXA2, ANXA3, ANXA4, APOA1, ATIC, CA1, CALR, EEF1A1, EEF1G, EEF2, ENO1, G6PD, GAPDH, HBA1/HBA2, HBB, HP, HSP90AB1, IDH1, LCP1, LPP, MZB1, NME1, PFN1, PKM, PPIA, PRDX2, PRDX5, RPSA, S100A4, SERPINA1, SND1, TUBB, YWHAZ
Gastrointestinal Disease	1.88E-03–7.04E-12	45	ACTB, AKR1B1, ALB, ANXA2, ANXA3, ANXA4, APOA1, ATIC, CA1, CALR, EEF1A1, EEF1B2, EEF2, ENO1, G6PD, GAPDH, HBA1/HBA2, HBB, HBD, HDGF, HNRNPK, HP, HSP90AB1, IDH1, ILF2, LCP1, LPP, MZB1, NME1, PDIA4, PFN1, PKM, PPA1, PPIA, PRDX2, PRDX5, PSMD7, RBBP4, RPSA, S100A4, SERPINA1, SND1, TUBB, UGDH, YWHAZ
**DEPs between Margin and Control**			
Cancer	1.88E-03–2.44E-14	45	ACTB, AHCY, ALB, ALDH1A1, ANXA3, ATIC, CA1, CS, EEF1A1, EEF1B2, EEF1D, EEF2, ENO1, FGB, GAPDH, HBA1/HBA2, HBB, HBD, HNRNPK, HP, HSP90AB1, HSPA1A/HSPA1B, HSPD1, IDH1, IMPDH2, KRT19, KRT34, LMNA, NME1, PDIA4, PKM, PPA1, PPIA, PRDX5, PSMD7, RBBP4, RPSA, S100A4, SERPINA1, SERPINB1, SND1, TAGLN, TXNRD1, UGDH, VIM
Gastrointestinal Disease	1.88E-03–2.44E-14	46	ACTB, AHCY, ALB, ALDH1A1, ANXA3, ATIC, CA1, CS, EEF1A1, EEF1B2, EEF1D, EEF2, ENO1, FGB, GAPDH, HBA1/HBA2, HBB, HBD, HNRNPK, HP, HSP90AB1, HSPA1A/HSPA1B, HSPD1, IDH1, IMPDH2, KRT19, KRT34, LMNA, NME1, PDIA4, PKM, PPA1, PPIA, PRDX5, PSMD7, RBBP4, RPSA, S100A4, SERPINA1, SERPINB1, SND1, TAGLN, TXNRD1, UGDH, VIM
Hepatic System Disease	1.88E-03–2.44E-14	33	ACTB, ALB, ALDH1A1, ANXA3, ATIC, CS, EEF1A1, EEF2, ENO1, FGB, HBA1/HBA2, HBB, HNRNPK, HP, HSP90AB1, HSPD1, IDH1, IMPDH2, KRT19, KRT34, LMNA, NME1, PDIA4, PKM, PPIA, RBBP4, RPSA, S100A4, SERPINA1, SND1, TXNRD1, UGDH, VIM
Organismal Injury and Abnormalities	1.88E-03–2.44E-14	46	ACTB, AHCY, ALB, ALDH1A1, ANXA3, ATIC, CA1, CS, EEF1A1, EEF1B2, EEF1D, EEF2, ENO1, FGB, GAPDH, HBA1/HBA2, HBB, HBD, HNRNPK, HP, HSP90AB1, HSPA1A/HSPA1B, HSPD1, IDH1, IMPDH2, KRT19, KRT34, LMNA, MZB1, NME1, PDIA4, PKM, PPA1, PPIA, PRDX5, PSMD7, RBBP4, RPSA, S100A4, SERPINA1, SERPINB1, SND1, TAGLN, TXNRD1, UGDH, VIM
Inflammatory Response	1.56E-03–2.95E-13	30	ACTB, AHCY, ALB, ANXA3, ATIC, CA1, EEF1A1, EEF2, ENO1, FGB, GAPDH, HBA1/HBA2, HBB, HP, HSP90AB1, HSPA1A/HSPA1B, HSPD1, IDH1, IMPDH2, MZB1, PKM, PPIA, PRDX5, PSMD7, RPSA, S100A4, SERPINA1, SERPINB1, SND1, VIM

*SCC*. The top five disease and disorder categories are shown in Table 4. Similar to ADC, the margin vs. center comparison was characterized by inflammatory responses and organismal injury and abnormalities; connective tissue, developmental, and hereditary disorders were indicated as well. On the other hand, center vs. control and margin vs. control were characterized by the same categories, hematological disease and organismal injury and abnormalities, comprising several cancers, including lung cancer annotations (for details, see S2 Material). Cancer was also indicated in the center vs. control comparison, together with gastrointestinal and hepatic system disease, whereas margin vs. control was characterized by immunological and inflammatory disease and inflammatory response.

**Table 4:** Top disease and disorder of differentially expressed proteins (DEPs) in SCC tumor.

Disease and Disorder	p-value	No of molecules	Proteins
**DEPs between Center and Margin**			
Inflammatory Response	4.92E-02–6.94E-06	13	ARHGDIB, CTSD, EHD1, KRT5, NAGK, NAPA, PKM, PPIB, PRDX4, RUVBL1, SERPINB1, SERPINH1, TF
Organismal Injury and Abnormalities	4.95E-02–6.94E-06	20	ARHGDIB, CAPZA1, CTSD, EHD1, ERO1A, HNRNPF, KRT19, KRT5, LDHA, MAPRE1, NAGK, NAPA, PKM, PPA1, PPIB, PRDX4, RUVBL1, SERPINB1, SERPINH1, TF
Connective Tissue Disorders	4.26E-02–4.93E-05	5	KRT5, MAPRE1, PPIB, SERPINH1, TF
Developmental Disorder	4.79E-02–4.93E-05	8	ARHGDIB, CTSD, EHD1, KRT5, LDHA, PPIB, SERPINH1, TF
Hereditary Disorder	4.79E-02–4.93E-05	9	ARHGDIB, CTSD, KRT5, LDHA, NAPA, PKM, PPIB, SERPINH1, TF
**DEPs between Center and Control**			
Cancer	1.11E-05–1.19E-19	58	ACO2, ACTB, ALB, ALDOA, CA1, CA2, CALR, CFL1, CLIC1, CS, CTSD, EEF1A1, EEF2, EIF4A2, ENO1, ERO1A, EZR, GAPDH, GSTP1, HBA1/HBA2, HBB, HBD, HNRNPH1, HNRNPK, HSP90AA1, HSP90AB1, HSP90B1, HSPA1A/HSPA1B, HSPA5, HSPA8, HSPD1, IDH1, KRT10, KRT17, KRT5, KRT6B, KRT8, LDHA, MAPRE1, NME1, P4HB, PDIA3, PDIA4, PGAM1, PGK1, RACK1, RPSA, S100A11, SELENBP1, SERPINH1, SFN, SOD2, TPSAB1/TPSB2, TUBA1B, VDAC1, YWHAB, YWHAE, YWHAZ
Gastrointestinal Disease	9.30E-06–1.19E-19	55	ACO2, ACTB, ALB, ALDOA, CA1, CA2, CALR, CFL1, CLIC1, CS, CTSD, EEF1A1, EEF2, EIF4A2, ENO1, ERO1A, EZR, GAPDH, GSTP1, HBA1/HBA2, HBB, HBD, HNRNPH1, HNRNPK, HSP90AA1, HSP90AB1, HSP90B1, HSPA5, HSPA8, HSPD1, IDH1, KRT10, KRT17, KRT5, KRT6B, KRT8, LDHA, MAPRE1, NME1, P4HB, PDIA3, PDIA4, PGK1, RACK1, RPSA, S100A11, SELENBP1, SERPINH1, SFN, SOD2, TPSAB1/TPSB2, VDAC1, YWHAB, YWHAE, YWHAZ
Hepatic System Disease	8.58E-06–1.19E-19	38	ACO2, ACTB, ALB, CA2, CLIC1, CS, CTSD, EEF1A1, EEF2, ENO1, GSTP1, HBA1/HBA2, HBB, HNRNPH1, HNRNPK, HSP90AA1, HSP90AB1, HSP90B1, HSPA5, HSPA8, HSPD1, IDH1, KRT10, KRT8, LDHA, MAPRE1, NME1, P4HB, PDIA3, PDIA4, PGK1, RACK1, RPSA, SERPINH1, SOD2, TPSAB1/TPSB2, VDAC1, YWHAZ
Organismal Injury and Abnormalities	1.11E-05–1.19E-19	59	ACO2, ACTB, ALB, ALDOA, CA1, CA2, CALR, CFL1, CLIC1, CS, CTSD, EEF1A1, EEF1G, EEF2, EIF4A2, ENO1, ERO1A, EZR, GAPDH, GSTP1, HBA1/HBA2, HBB, HBD, HNRNPH1, HNRNPK, HSP90AA1, HSP90AB1, HSP90B1, HSPA1A/HSPA1B, HSPA5, HSPA8, HSPD1, IDH1, KRT10, KRT17, KRT5, KRT6B, KRT8, LDHA, MAPRE1, NME1, P4HB, PDIA3, PDIA4, PGAM1, PGK1, RACK1, RPSA, S100A11, SELENBP1, SERPINH1, SFN, SOD2, TPSAB1/TPSB2, TUBA1B, VDAC1, YWHAB, YWHAE, YWHAZ
Hematological Disease	9.30E-06–1.07E-17	40	ACO2, ACTB, ALB, ALDOA, CA2, CALR, CFL1, EEF1A1, ENO1, GAPDH, GSTP1, HBA1/HBA2, HBB, HSP90AA1, HSP90AB1, HSP90B1, HSPA1A/HSPA1B, HSPA5, HSPA8, HSPD1, IDH1, KRT10, KRT17, KRT5, LDHA, NME1, P4HB, PDIA3, PGK1, RACK1, RPSA, S100A11, SELENBP1, SERPINH1, SOD2, TPSAB1/TPSB2, TUBA1B, VDAC1, YWHAE, YWHAZ
**DEPs between Margin and Control**			
Hematological Disease	9.53E-06–3.66E-19	44	ACO2, ACTB, ALB, ALDOA, CA2, CALR, CFL1, EEF1A1, ENO1, G6PD, GAPDH, GSTP1, HBA1/HBA2, HBB, HSP90AA1, HSP90AB1, HSP90B1, HSPA1A/HSPA1B, HSPA5, HSPA8, HSPB1, HSPD1, IDH1, KRT10, KRT17, KRT5, LDHA, MYH9, NME1, P4HB, PDIA3, PGK1, PPIA, PRDX4, RACK1, RPSA, S100A11, S100A9, SELENBP1, SOD2, TUBA1B, VDAC1, YWHAE, YWHAZ
Immunological Disease	9.53E-06–3.66E-19	50	ACO2, ACTB, ALB, ALDOA, APOA1, CA1, CA2, CALR, CFL1, EEF1A1, EEF1G, EEF2, ENO1, G6PD, GAPDH, GSTP1, HBA1/HBA2, HBB, HSP90AA1, HSP90AB1, HSP90B1, HSPA1A/HSPA1B, HSPA5, HSPA8, HSPB1, HSPD1, IDH1, KRT10, KRT17, KRT5, LDHA, MAPRE1, MYH9, NAGK, NME1, P4HB, PDIA3, PGK1, PPIA, PRDX4, RACK1, RPSA, S100A11, S100A9, SELENBP1, SOD2, TUBA1B, VDAC1, YWHAE, YWHAZ
Inflammatory Disease	7.46E-06–3.66E-19	42	ACO2, ACTB, ACTR3, ALB, ALDOA, APOA1, CA1, CA2, CALR, CFL1, EEF1A1, EEF1G, EEF2, ENO1, G6PD, GAPDH, GSTP1, HBA1/HBA2, HBB, HSP90B1, HSPA1A/HSPA1B, HSPA5, HSPA8, HSPD1, KRT10, KRT17, KRT5, KRT8, MAPRE1, MYH9, NAGK, P4HB, PDIA3, PGK1, PPIA, RACK1, RPSA, S100A11, S100A9, SELENBP1, SFN, SOD2
Inflammatory Response	7.46E-06–3.66E-19	47	ACO2, ACTB, ALB, ALDOA, APOA1, CA1, CA2, CALR, CCT8, CFL1, EEF1A1, EEF1G, EEF2, ENO1, GAPDH, GSTP1, HBA1/HBA2, HBB, HSP90AA1, HSP90AB1, HSP90B1, HSPA1A/HSPA1B, HSPA5, HSPA8, HSPD1, IDH1, KRT10, KRT17, KRT5, KRT8, MAPRE1, MYH9, NAGK, P4HB, PDIA3, PGAM1, PGK1, PPIA, PRDX4, RACK1, RPSA, S100A11, S100A9, SELENBP1, SFN, SOD2, TTR
Organismal Injury and Abnormalities	1.17E-05–3.66E-19	69	ACO2, ACTB, ACTR3, ALB, ALDOA, APOA1, CA1, CA2, CALR, CCT8, CFL1, CLIC1, CS, EEF1A1, EEF1G, EEF2, EIF4A2, ENO1, EZR, G6PD, GAPDH, GSTP1, HBA1/HBA2, HBB, HBD, HNRNPH1, HNRNPK, HSP90AA1, HSP90AB1, HSP90B1, HSPA1A/HSPA1B, HSPA5, HSPA8, HSPB1, HSPD1, IDH1, KRT10, KRT17, KRT5, KRT6B, KRT8, LDHA, MAPRE1, MYH9, MYL6, NAGK, NME1, P4HB, PDIA3, PDIA4, PGAM1, PGK1, PPA1, PPIA, PPIB, PRDX4, RACK1, RPSA, S100A11, S100A9, SELENBP1, SFN, SOD2, TTR, TUBA1B, VDAC1, YWHAB, YWHAE, YWHAZ

#### Molecular and cellular functions

*ADC*. The top five molecular and cellular functions categories are shown in Table 5. The margin vs. center comparison was characterized by cellular movement, cell morphology, cell death and survival, cellular development, and lipid metabolism. On the other hand, center vs. control and margin vs. control were characterized by the same four categories: cellular compromise, cellular movement, cell death and survival, and free radical scavenging. Cellular development was also indicated in the center vs. control comparison, and small molecule biochemistry, in margin vs. control.

**Table 5:** Molecular and cellular functions of differentially expressed proteins (DEPs) in ADC tumor.

Molecular and Cellular Functions	p-value	No of molecules	Proteins
**DEPs between Center and Margin**			
Cellular Movement	2.00E-02–3.88E-06	10	ALB, ALDH2, DPYSL3, HSPD1, LCP1, LGALS1, LMNA, MSN, TUBB, WARS1
Cell Morphology	2.02E-02–2.99E-05	8	ALB, DPYSL3, HSPD1, LCP1, LGALS1, LMNA, MSN, MYL3
Cell Death and Survival	1.95E-02–4.80E-05	12	ALB, ALDH2, CCT2, CS, DPYSL3, HSPD1, LCP1, LGALS1, LMNA, MSN, SNX6, TUBB
Cellular Development	1.88E-02–1.81E-04	7	ALB, DPYSL3, HSPD1, LCP1, LGALS1, LMNA, MSN
Lipid Metabolism	1.86E-02–1.82E-04	6	ALB, CS, LGALS1, LMNA, LTA4H, MSN
**DEPs between Center and Control**			
Cellular Compromise	1.44E-12–2.95E-13	16	ALB, ANXA2, ANXA3, APOA1, EEF1A1, EEF2, HBB, HP, HSP90AB1, IDH1, ILF2, PKM, PPIA, PSMD7, SERPINA1, TUBB
Cell Death and Survival	1.88E-03–1.16E-12	35	ACTB, AKR1B1, ALB, ANXA2, ANXA4, APOA1, ATIC, CALR, EEF1A1, EEF2, ENO1, G6PD, GAPDH, HBA1/HBA2, HBB, HDGF, HNRNPK, HSP90AB1, IDH1, ILF2, MZB1, NME1, PFN1, PKM, PPIA, PRDX2, PRDX5, PSMD7, RBBP4, RPSA, S100A4, SERPINA1, SND1, TUBB, YWHAZ
Free Radical Scavenging	1.76E-03–1.41E-12	18	ACTB, AKR1B1, ALB, ANXA2, APOA1, G6PD, HBA1/HBA2, HBB, HNRNPK, HP, HSP90AB1, IDH1, PFN1, PPIA, PRDX2, PRDX5, SERPINA1, YWHAZ
Cellular Movement	1.76E-03–2.52E-12	29	ACTB, AKR1B1, ALB, ANXA2, ANXA3, APOA1, CALR, ENO1, G6PD, GAPDH, HDGF, HNRNPK, HP, HSP90AB1, IDH1, LCP1, LPP, NME1, PFN1, PKM, PPIA, PRDX2, RPSA, S100A4, SERPINA1, SND1, TUBB, UGDH, YWHAZ
Cellular Development	1.87E-03–2.93E-08	25	ACTB, AKR1B1, ANXA2, APOA1, CALR, EEF1A1, EEF1B2, G6PD, GAPDH, HDGF, HNRNPK, ILF2, LCP1, NME1, PFN1, PKM, PPIA, PRDX2, RPSA, S100A4, SERPINA1, SND1, TUBB, UGDH, YWHAZ
**DEPs between Margin and Control**			
Cellular Compromise	1.88E-03–2.95E-13	17	ALB, ANXA3, EEF1A1, EEF2, FGB, HBB, HP, HSP90AB1, HSPA1A/HSPA1B, IDH1, IMPDH2, PKM, PPIA, PSMD7, S100A4, SERPINA1, SERPINB1
Cell Death and Survival	1.88E-03–5.12E-10	34	ACTB, ALB, ALDH1A1, ATIC, CS, EEF1A1, EEF1D, EEF2, ENO1, GAPDH, HBA1/HBA2, HBB, HNRNPK, HSP90AB1, HSPA1A/HSPA1B, HSPD1, IDH1, IMPDH2, KRT19, LMNA, MZB1, NME1, PKM, PPIA, PRDX5, PSMD7, RBBP4, RPSA, S100A4, SERPINA1, SERPINB1, SND1, TXNRD1, VIM
Cellular Movement	1.56E-03–1.05E-09	27	ACTB, AHCY, ALB, ANXA3, ENO1, FGB, GAPDH, HNRNPK, HP, HSP90AB1, HSPA1A/HSPA1B, HSPD1, IDH1, KRT19, LMNA, NME1, PKM, PPIA, RPSA, S100A4, SERPINA1, SERPINB1, SND1, TAGLN, TXNRD1, UGDH, VIM
Free Radical Scavenging	7.50E-04–2.62E-08	13	ACTB, ALB, HBA1/HBA2, HBB, HNRNPK, HP, HSP90AB1, IDH1, PPIA, PRDX5, SERPINA1, TAGLN, TXNRD1
Small Molecule Biochemistry	1.88E-03–1.86E-07	26	AHCY, ALB, ALDH1A1, CS, EEF1A1, EEF1B2, ENO1, GAPDH, HBA1/HBA2, HBB, HSP90AB1, HSPA1A/HSPA1B, HSPD1, IDH1, IMPDH2, LMNA, MZB1, NME1, PKM, PPA1, PRDX5, RPSA, SERPINA1, TXNRD1, UGDH, VIM

*SCC*. The top five molecular and cellular function categories are shown in Table 6. The margin vs. center comparison was characterized by post-translational modification, protein folding, cellular development, cellular growth and proliferation, and cellular assembly and organization. On the other hand, center vs. control and margin vs. control were characterized by the same four categories: cellular movement, cell death and survival, cellular compromise, and post-translational modification. Cellular function and maintenance were also indicated in the center vs. control comparison, and protein folding, in margin vs. control.

**Table 6:** Molecular and cellular functions of differentially expressed proteins (DEPs) in SCC tumor.

Molecular and Cellular Functions	p-value	No of molecules	Proteins
**DEPs between Center and Margin**			
Post-Translational Modification	3.62E-02–1.77E-05	6	CAPZA1, CTSD, ERO1A, PRDX4, RUVBL1, TF
Protein Folding	1.77E-05–1.77E-05	2	ERO1A, PRDX4
Cellular Development	4.95E-02–2.36E-05	12	ARHGDIB, CTSD, EHD1, ERO1A, KRT19, LDHA, MAPRE1, PKM, PRDX4, RUVBL1, SERPINH1, TF
Cellular Growth and Proliferation	4.95E-02–2.36E-05	12	ARHGDIB, CTSD, EHD1, ERO1A, KRT19, LDHA, MAPRE1, PKM, PRDX4, RUVBL1, SERPINH1, TF
Cellular Assembly and Organization	4.40E-02–8.84E-05	9	CTSD, EHD1, KRT19, MAPRE1, NAPA, PKM, PPIB, SERPINH1, TF
**DEPs between Center and Control**			
Cellular Movement	9.72E-06–9.28E-18	42	ACO2, ACTB, ALB, ALDOA, CA2, CALR, CFL1, CLIC1, CTSD, ENO1, EZR, GAPDH, HNRNPK, HSP90AA1, HSP90AB1, HSP90B1, HSPA1A/HSPA1B, HSPA5, HSPA8, HSPD1, IDH1, KRT10, KRT17, KRT6B, KRT8, LDHA, MAPRE1, NME1, P4HB, PDIA3, PPIB, RACK1, RPSA, S100A11, SELENBP1, SERPINH1, SFN, SOD2, TPSAB1/TPSB2, VDAC1, YWHAE, YWHAZ
Cell Death and Survival	7.54E-06–7.88E-16	47	ACO2, ACTB, ALB, ALDOA, CA2, CALR, CFL1, CS, CTSD, EEF1A1, EEF2, ENO1, EZR, GAPDH, GSTP1, HBA1/HBA2, HBB, HNRNPH1, HNRNPK, HSP90AA1, HSP90AB1, HSP90B1, HSPA1A/HSPA1B, HSPA5, HSPA8, HSPD1, IDH1, KRT10, KRT17, KRT8, LDHA, NME1, P4HB, PDIA3, PPIB, RACK1, RPSA, S100A11, SELENBP1, SERPINH1, SFN, SOD2, TPSAB1/TPSB2, VDAC1, YWHAB, YWHAE, YWHAZ
Cellular Compromise	5.99E-06–1.51E-11	21	ALB, ALDOA, CALR, CTSD, EEF1A1, EEF2, ERO1A, GSTP1, HBB, HSP90AA1, HSP90AB1, HSP90B1, HSPA1A/HSPA1B, HSPA5, HSPA8, HSPD1, IDH1, P4HB, PGAM1, S100A11, SERPINH1
Cellular Function and Maintenance	7.55E-06–1.51E-11	36	ACTB, ALB, ALDOA, CA2, CALR, CFL1, CLIC1, CTSD, EEF2, ERO1A, EZR, GAPDH, GSTP1, HBA1/HBA2, HBB, HNRNPK, HSP90AA1, HSP90AB1, HSP90B1, HSPA1A/HSPA1B, HSPA5, HSPA8, HSPD1, IDH1, KRT17, KRT6B, KRT8, LDHA, MAPRE1, P4HB, PPIB, RACK1, SERPINH1, SOD2, VDAC1, YWHAE
Post-Translational Modification	3.35E-07–4.22E-11	9	CALR, ERO1A, HSP90AA1, HSP90AB1, HSPA1A/HSPA1B, HSPA5, HSPA8, HSPD1, PDIA3
**DEPs between Margin and Control**			
Cellular Movement	1.23E-06–3.24E-20	47	ACO2, ACTB, ACTR3, ALB, ALDOA, APOA1, CA2, CALR, CFL1, CLIC1, ENO1, EZR, G6PD, GAPDH, HNRNPK, HSP90AA1, HSP90AB1, HSP90B1, HSPA1A/HSPA1B, HSPA5, HSPA8, HSPB1, HSPD1, IDH1, KRT10, KRT17, KRT6B, KRT8, LDHA, MAPRE1, MYH9, NME1, P4HB, PDIA3, PPIA, PPIB, RACK1, RPSA, S100A11, S100A9, SELENBP1, SFN, SOD2, TTR, VDAC1, YWHAE, YWHAZ
Cell Death and Survival	8.81E-06–3.51E-16	52	ACO2, ACTB, ALB, ALDOA, APOA1, CA2, CALR, CCT8, CFL1, CS, EEF1A1, EEF2, ENO1, EZR, G6PD, GAPDH, GSTP1, HBA1/HBA2, HBB, HNRNPH1, HNRNPK, HSP90AA1, HSP90AB1, HSP90B1, HSPA1A/HSPA1B, HSPA5, HSPA8, HSPB1, HSPD1, IDH1, KRT10, KRT17, KRT8, LDHA, NME1, P4HB, PDIA3, PPIA, PPIB, PRDX4, RACK1, RPSA, S100A11, S100A9, SELENBP1, SFN, SOD2, TTR, VDAC1, YWHAB, YWHAE, YWHAZ
Post-Translational Modification	5.90E-07–3.26E-15	12	CALR, CCT8, HSP90AA1, HSP90AB1, HSPA1A/HSPA1B, HSPA5, HSPA8, HSPB1, HSPD1, PDIA3, PPIA, PRDX4
Protein Folding	5.90E-07–3.26E-15	12	CALR, CCT8, HSP90AA1, HSP90AB1, HSPA1A/HSPA1B, HSPA5, HSPA8, HSPB1, HSPD1, PDIA3, PPIA, PRDX4
Cellular Compromise	2.54E-06–4.06E-14	24	ALB, ALDOA, APOA1, CALR, CCT8, EEF1A1, EEF2, GSTP1, HBB, HSP90AA1, HSP90AB1, HSP90B1, HSPA1A/HSPA1B, HSPA5, HSPA8, HSPD1, IDH1, P4HB, PGAM1, PPIA, PRDX4, S100A11, S100A9, TTR

#### Upstream regulator

*ADC*. The upstream regulators are shown in S1 Material. In the center vs. margin comparison L-triiodothyronine (p = 3.31E-04; z score -2.000) was marked as significantly inhibited regulator. In the center vs. control comparison La-related protein 1 (LARP1; p = 2.09E-05; z-score -2.000); IND S1 (p = 9.09E-08; z-score -2.000); IND S7 (p = 2.52E-07; z-score -2.000) and MEL S3 (p = 2.92E-07; z-score -2.000) were marked as inhibited regulators whereas interleukin-15 (IL15; p = 2.40E-05; z-score 2.000); prostate cancer gene expression marker 1 (PCGEM1; p = 1.83E-08; z-score 2.201); lysine demethylase 8 (KDM8; p = 3.32E-07 z-score 2.219); testosterone (p = 9.11E-04; z-score 2.236) and nuclear factor erythroid 2-related factor 2 (NFE2L2; p = 2.42E-04; z-score 2.391) were activated regulators. In the margin vs. control comparison 5-fluorouracil (p = 5.79E-04; z-score -2.000); caseinolytic protease P (CLPP; p = 7.91E-06; z-score -2.000) were inhibited regulators, whereas NFE2L2 (p = 2.47E-06; z-score 2.184); PCGEM1 (p = 1.83E-08; z-score 2.207); N-myc proto-oncogene protein (MYCN; p = 7.11E-15; z-score 2.382), serine/threonine kinase 11 (STK11; p = 2.73E-10; z-score 2.530) and Myc proto-oncogene protein (MYC; p = 8.90E-10; z-score 2.789) were activated regulators.

*SCC*. The upstream regulators are shown in S2 Material. No upstream regulators were identified in the center vs. margin comparison. In the center vs. control comparison sirolimus (p = 3.20E-13; z-score -3.492); CD 437 (p = 1.41E-13; z-score 3.464); ST1926 (p = 3.19E-13; z-score -3.317); tretinoin (p = 2.19E-04; z-score -2.885) and CLPP (p = 3.02E-10; z-score -2.646) were marked as inhibited regulators, whereas 6-hydroxydopamine (6-OHDA; p = 7.79E-12; z-score 2.985); NFE2L2 (p = 9.75E-11; z-score 3.050); beta-estradiol (p = 5.74E-20; z-score 3.229); lipopolysaccharide (p = 2.97E-06; z-score 3.379) and insulin (p = 2.99E-12; z-score 3.411) were activated regulators. In the margin vs. control comparison sirolimus (p = 2.30E-14; z-score -3.758); CD 437 (p = 8.31E-13; z-score -3.464); ST1926 (p = 1.61E-12; z-score -3.317); tretinoin (p = 7.08E-05; z-score -3.053); CLPP (p = 8.27E-10; z-score -2.646) were inhibited regulators, whereas 1.2-dithiol-3-thione (p = 2.41E-11; z-score 3.289); lipopolysaccharide (p = 7.25E-08; z-score 3.318); insulin (p = 8.67E-15; z-score 3.3570); NFE2L2 (p = 4.74E-11; z-score 3.653) and MYC (p = 5.26E-13; z-score 3.684) were activated regulators.

### STRING analysis

Complete sets of all the STRING results are presented in S3–S8 Materials.

#### ADC

**Fig 4:**
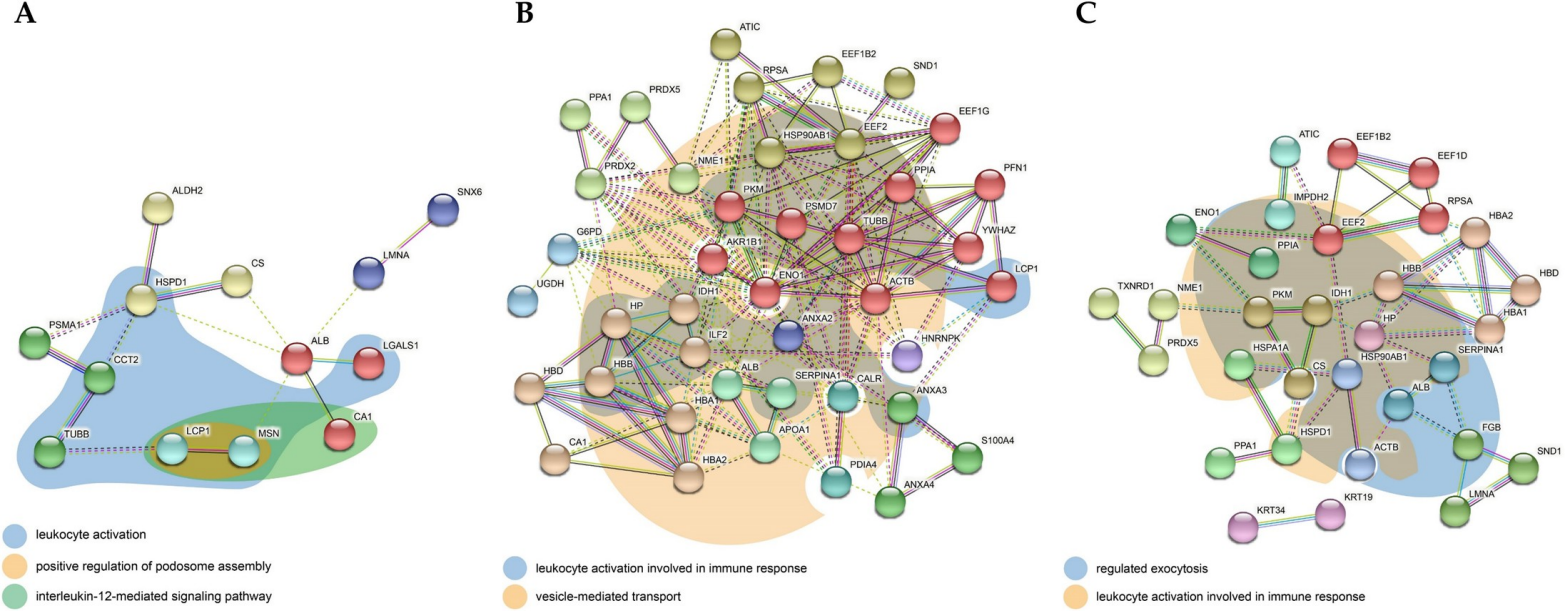
Protein-protein interaction network of differentially expressed proteins in (A) margin vs. center, (B) center vs. control, (C) margin vs. control comparison for ADC. The network nodes represent proteins while the edges represent predicted functional associations. There are 5 types of associations presented: neighborhood (green), experimental (purple), text mining (yellow), database (light blue), coexpression (black) evidence. The color of the nodes represents cluster membership. Inter-cluster edges are depicted by dashed lines. The colored areas represent functional pathways.

The margin vs. center comparison indicated 19 significant interactions specified by GO analysis. Fig 4A highlights interactions related to the interleukin-12-mediated signaling pathway, leukocyte activation, and the positive regulation of podosome assembly. The center vs. control comparison indicated 158 significant interactions specified by GO analysis. Fig 4B highlights interactions related to leukocyte activation involved in the immune response and vesicle-mediated transport. The margin vs. control comparison indicated 139 significant interactions specified by GO analysis. Fig 4C highlights interactions related to regulated exocytosis and leukocyte activation involved in the immune response.

#### SCC

**Fig 5:**
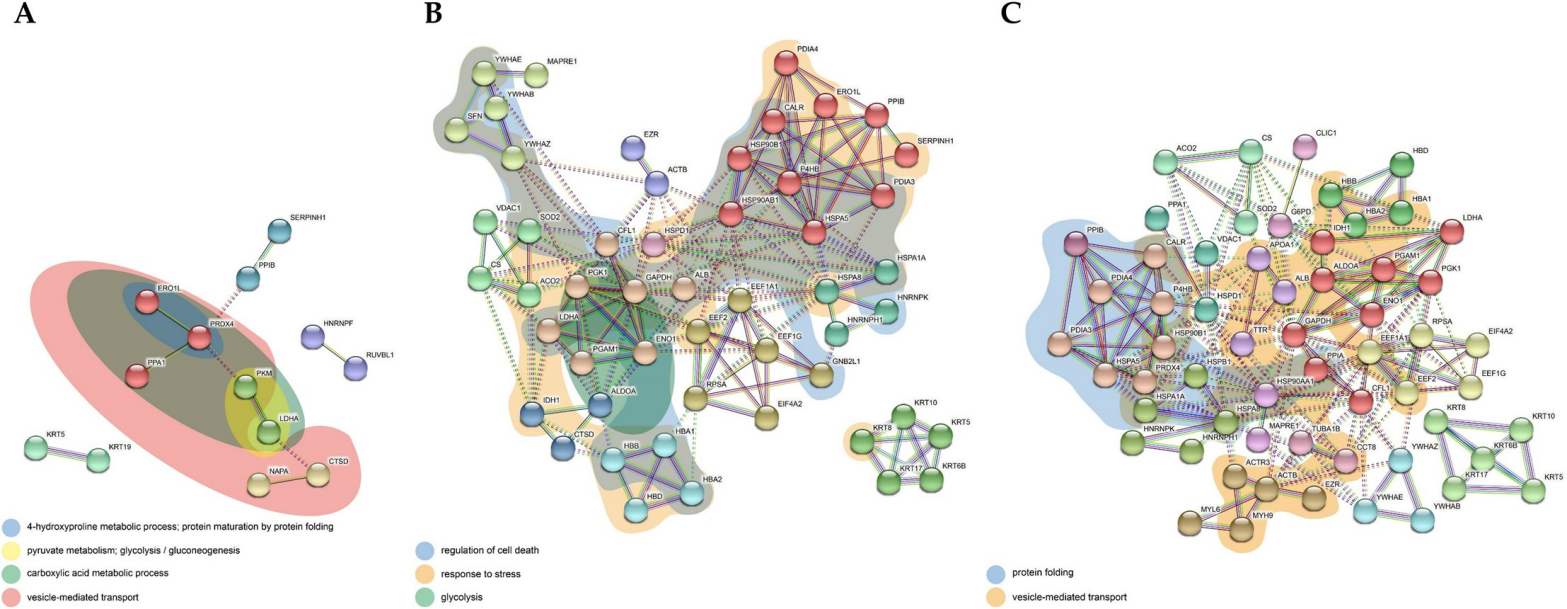
Protein-protein interaction network of differentially expressed proteins in (A) margin vs. center, (B) center vs. control, (C) margin vs. control comparison for SCC. The network nodes represent proteins while the edges represent predicted functional associations. There are 5 types of associations presented: neighborhood (green), experimental (purple), text mining (yellow), database (light blue), coexpression (black) evidence. The color of the nodes represents cluster membership. Inter-cluster edges are depicted by dashed lines. The colored areas represent functional pathways.

The margin vs. center comparison indicated four significant interactions specified by GO analysis. Fig 5A highlights interactions related to the 4-hydroxyproline metabolic process, protein maturation by protein folding, vesicle-mediated transport, pyruvate metabolism, glycolysis/gluconeogenesis, the carboxylic acid metabolic process, and vesicle-mediated transport. The center vs. control comparison indicated 365 significant interactions specified by GO analysis. Fig 5B highlights interactions related to the regulation of cell death, glycolysis, and response to stress. The margin vs. control comparison indicated 373 significant interactions specified by GO analysis. Fig 5C highlights interactions related to protein folding and vesicle-mediated transport.

### IPA networks

#### ADC

**Fig 6:**
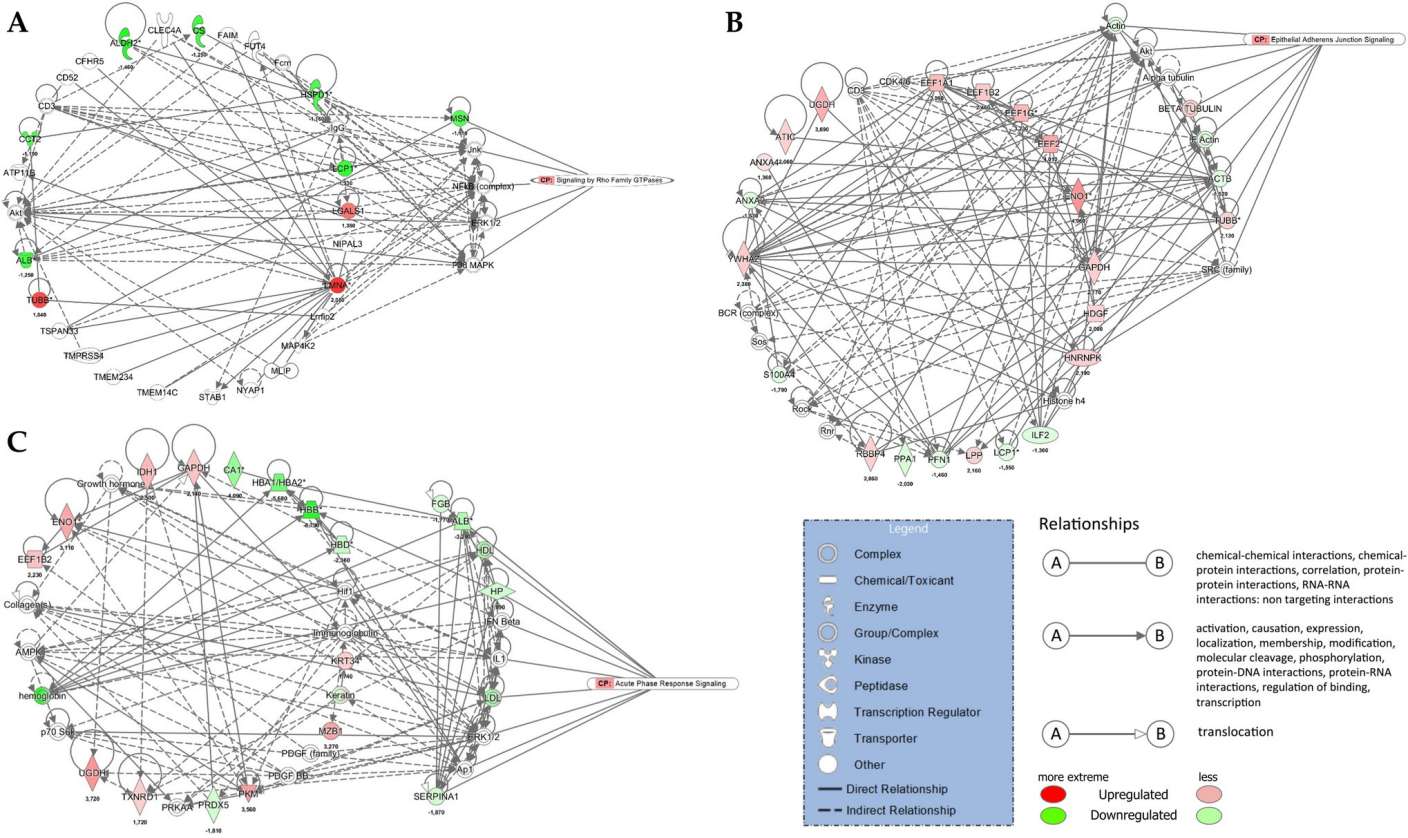
IPA networks for ADC. (A) margin vs. center comparison in ADC. Cellular Movement, Hematological Disease, Immunological Disease Network is presented together with Signaling with Rho GTPases canonical pathway; (B) center vs. control comparison in ADC. Cellular Movement, Hematological Disease, Immunological Disease is presented together with Signaling with Epithelial Adherens Junction Signaling canonical pathway and (C) margin vs. control comparison in ADC. Cardiovascular Disease, Free Radical Scavenging, Small Molecule Biochemistry is presented together with Acute Phase Response Signaling canonical pathway.

For the margin vs. center comparison, IPA indicated two networks: (i) cellular movement, hematological disease, and immunological disease, consisting of 10 proteins, and (ii) cell death and survival, cellular movement, and organismal injury and abnormalities, consisting of seven proteins. The cellular movement, hematological disease, immunological disease network is shown in Fig 6A, overlaid with signaling by the Rho GTPase canonical pathway. The center vs. control comparison indicated four networks: (i) cellular movement, hematological disease, and immunological disease, consisting of 22 proteins; (ii) cell death and survival, connective tissue disorders, and free radical scavenging, consisting of 15 proteins; (iii) cancer, endocrine system disorders, and organismal injury and abnormalities, consisting of seven proteins; and (iv) cell death and survival, embryonic development, and post-translational modification, consisting of two proteins. The cellular movement, hematological disease, immunological disease network is shown in Fig 6B overlaid with the epithelial adherens junction signaling canonical pathway. The margin vs. control comparison indicated three networks: (i) cardiovascular disease, free radical scavenging, and small molecule biochemistry, consisting of 18 proteins; (ii) cancer, endocrine system disorders, and organismal injury and abnormalities, consisting of 15 proteins; and (iii) cancer, cellular growth and proliferation, and cellular movement, consisting of 13 proteins. The cardiovascular disease, free radical scavenging, and small molecule biochemistry network is shown in Fig 6C overlaid with the acute phase response signaling canonical pathway.

#### SCC

**Fig 7:**
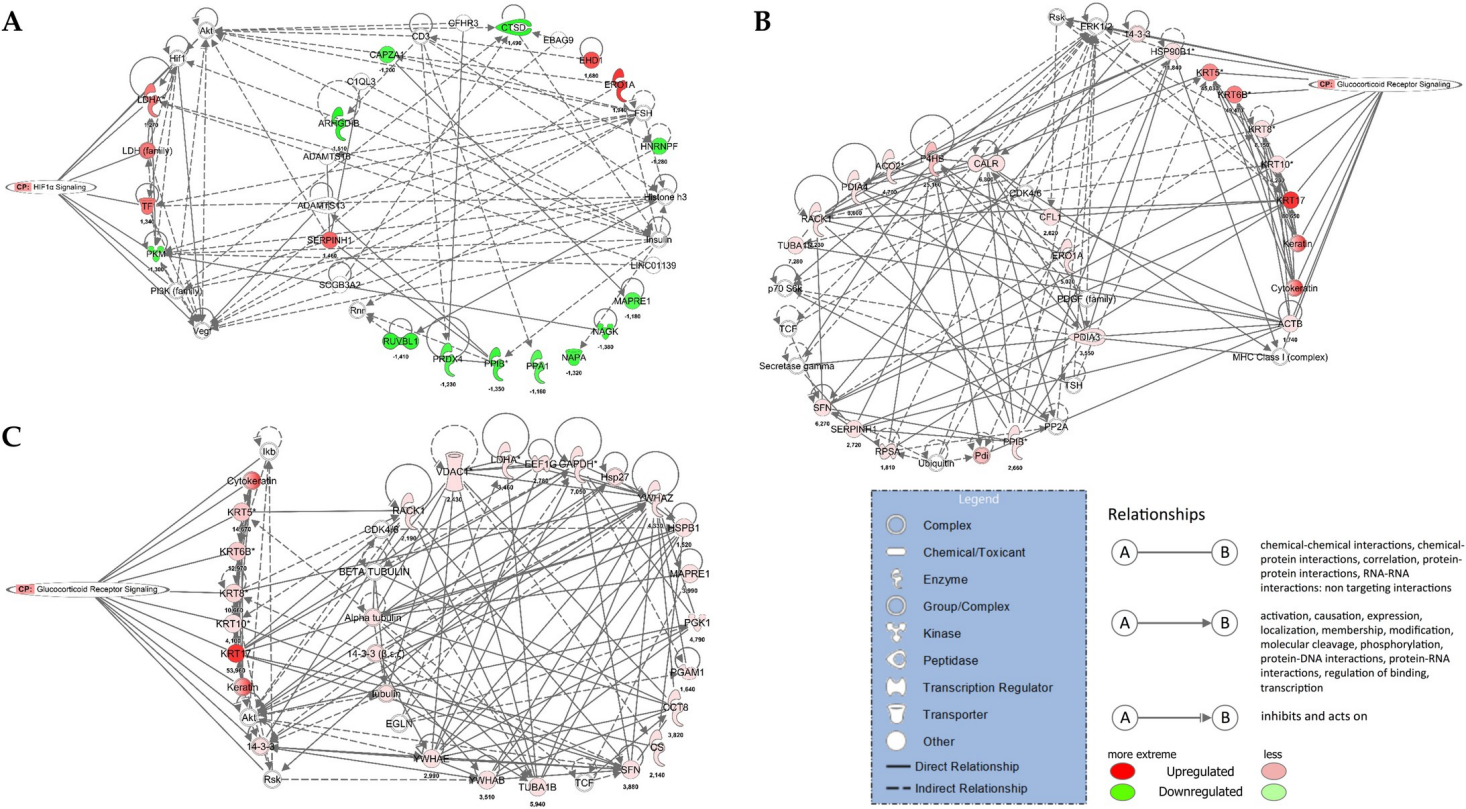
IPA networks for SCC. (A) center vs. margin comparison in SCC. Connective tissue disorders, post-translational modification, protein folding is presented together with HIF 1α signaling canonical pathway; (B) center vs. control comparison in SCC. Cell morphology, embryonic development, hair and skin development and function is presented together with glucocorticoid receptor signaling canonical pathway and (C) margin vs. control comparison in SCC. Cell morphology, embryonic development, hair and skin development and function is presented together with glucocorticoid receptor signaling canonical pathway.

For the margin vs. center comparison, IPA indicated two networks: (i) connective tissue disorders, post-translational modification, and protein folding, consisting of 17 proteins, and (ii) cell morphology, embryonic development, and hair and skin development and function, consisting of three proteins. The connective tissue disorders, post-translational modification, protein folding network is shown in Fig 7A overlaid with HIF 1α signaling canonical pathway. The center vs. control comparison indicated five networks: (i) cell morphology, embryonic development, and hair and skin development and function, consisting of 20 proteins; (ii) carbohydrate metabolism, cellular movement, and hematological disease, consisting of 16 proteins; (iii) cancer, gastrointestinal disease, and hepatic system disease, consisting of 12 proteins; (iv) cardiovascular disease, cell death and survival, and molecular transport, consisting of seven proteins; and (v) cancer, organismal injury and abnormalities, and renal and urological disease, consisting of five proteins. The cell morphology, embryonic development, hair and skin development and function network is shown in Fig 7B overlaid with glucocorticoid receptor signaling canonical pathway. The margin vs. control comparison indicated the same networks as the center vs. control comparison overlaid with glucocorticoid receptor signaling (Fig 7C).

### Comparison of SCC and ADC

**Fig 8:**
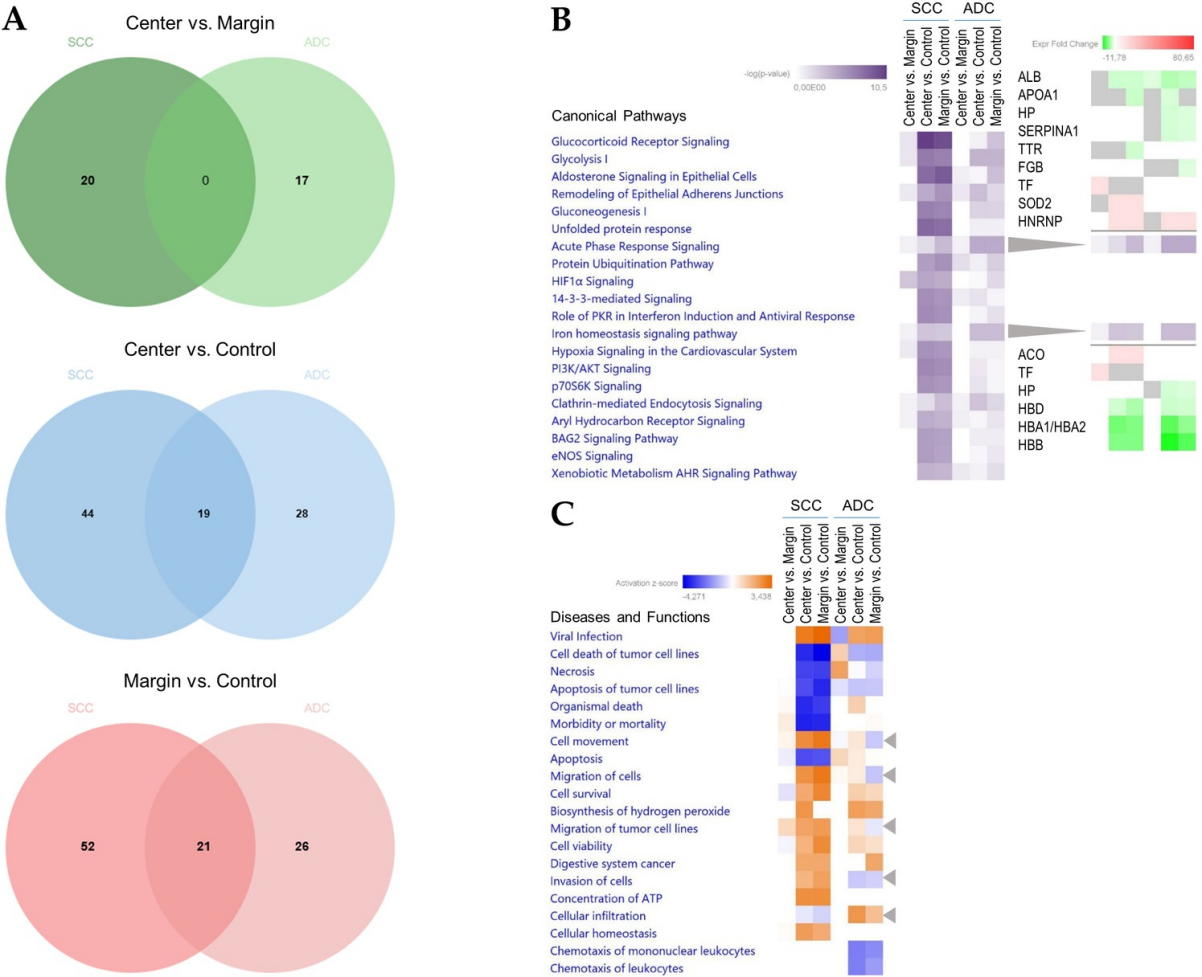
Venn diagrams and IPA comparison analysis for proteins in each NSCLC subtype, ADC and SCC. (A) Venn diagrams comparing proteins identified in SCC or ADC for the following comparisons: center vs. margin, center vs. control, and margin vs. control. (B) The heat map of the top 20 Canonical Pathways (left panel) across different comparisons displaying the Fisher’s exact test p-value (expressed as–log[p-value]). Proteins positively or negatively correlated with Acute phase response signaling or Iron homeostasis signaling are shown as a protein expression fold change heat maps (right panel). (C) The heat map displays the z-scores (abs(z) ≥ 2 in at least on comparison) from top 20 Diseases and Functions analysis (orange and blue rectangles represent activation and suppression, respectively).

Venn diagrams and the results of the enrichment analysis performed across the tested comparisons for SCC and ADC are presented in Fig 8. Unique proteins were identified for the center vs. margin comparison (20 for SCC and 17 for ADC); however, no common proteins were identified (Fig 8A). On the other hand, the center vs. control and margin vs. control comparisons revealed 19 and 21 common proteins, respectively (Fig 8A). Among them, 16 proteins were common to both cancer subtypes, while three (calreticulin (CALR), eukaryotic translation elongation factor 1 gamma (EEF1G), and YWHAZ protein (YWHAZ)) were unique for center vs. control and five (citrate synthase (CS), heat shock 70 kDa protein (HSPA1A), mitochondrial heat shock 60 kD protein (1 HSPD1), PPA1, and inorganic pyrophosphatase (PPIA) were unique to margin vs. control. In addition, several unique proteins were detected in each comparison: center vs. control: 44 proteins unique to SCC and 28 to ADC; margin vs. control: 52 proteins unique to SCC and 26 to ADC.

**Fig 9:**
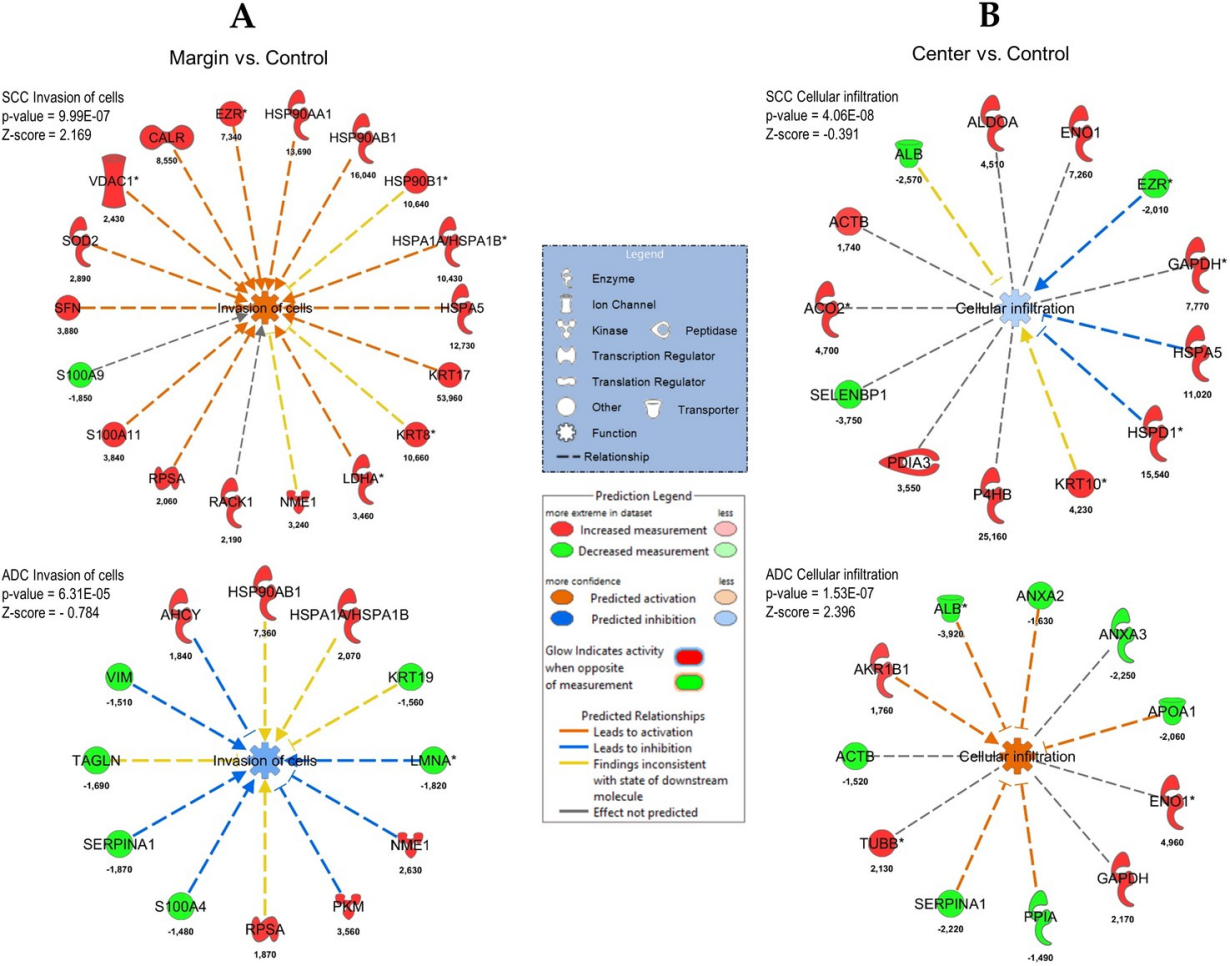
Invasion of cells and cellular infiltration functions overlaid with proteins from the particular comparison for SCC and ADC. (A) margin vs. control; (B) center vs. control. Each network shows the proteins in the particular analysis that have a causal or correlative relationship with the function and indicates how they might increase or decrease selected function. Legends are a graphical explanation of icons, lines and colors used in a graph. Difference in protein abundance is indicated below each protein.

Simultaneous pathway, disease, and function enrichment analyses of two tested subtypes of NSCLC are shown in Fig 8B and 8C. The proteins in SCC and ADC enriched similar canonical pathways or diseases and functions; however, some unique patterns could also be observed. In general, greater p-values for canonical pathways were noted for SSC, but some exceptions were identified (Fig 8B). For center vs. control and margin vs. control comparisons, acute phase response signaling and iron homeostasis signaling were more pronounced in ADC. Disease and function comparisons for margin vs. control revealed opposite predicted activation states for functions related to cell movement and migration, activated in SCC and inhibited in ADC (Fig 8C). The invasion of cells was activated in SCC according to both comparisons and inhibited in ADC (Figs 8C and 9A). The reverse activation state was detected for cellular infiltration (Figs 8C and 9B). Among the top 20 diseases and functions, the inhibition of leucocyte chemotaxis was only identified in the center vs. control (z-score = -2.177, -2.159) and margin vs. control (z-score = -1.941, -1.673) comparisons for ADC.

**Fig 10:**
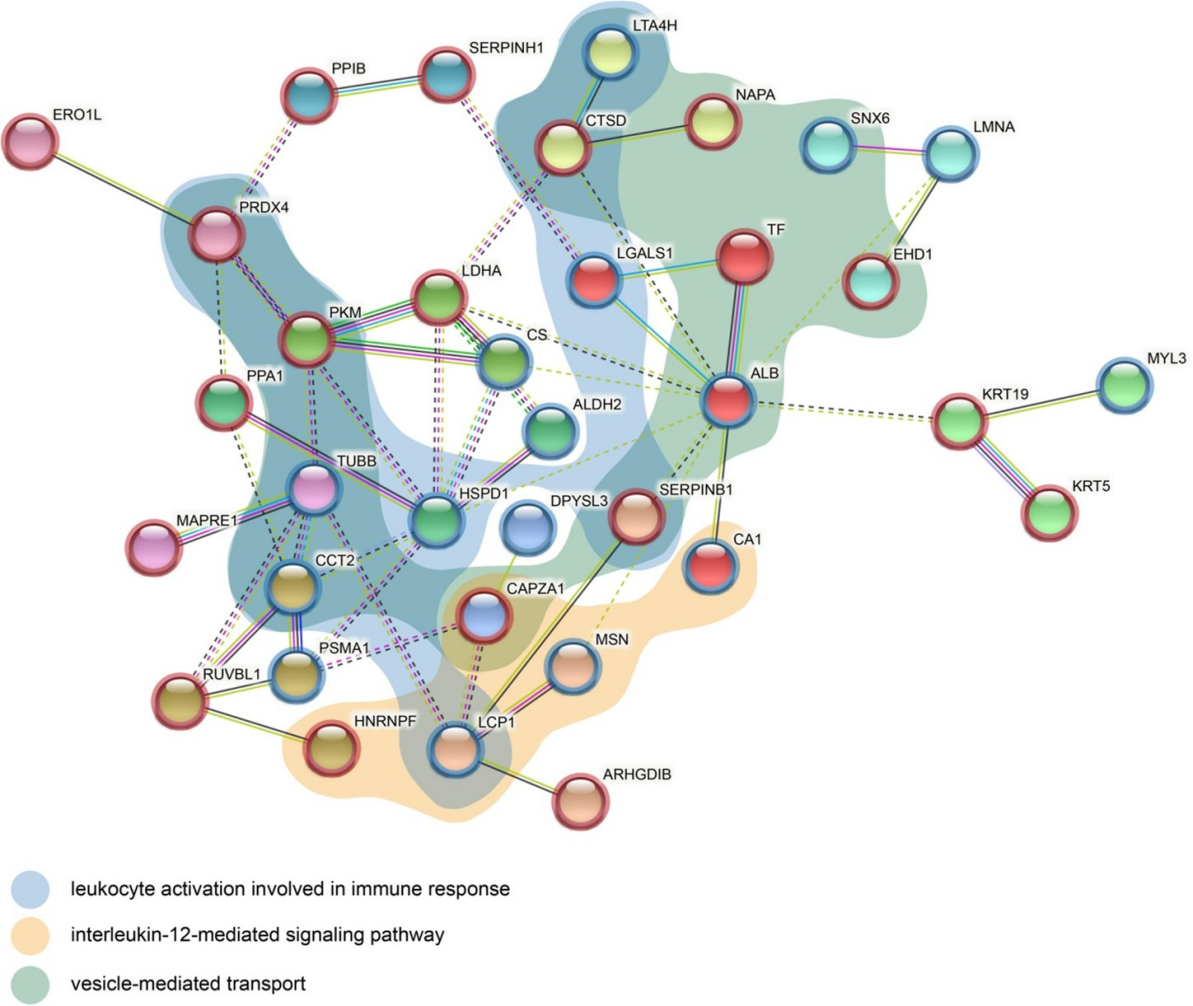
Combined protein-protein interaction network of differentially expressed proteins in center vs. margin comparison for both SCC (node red border) and ADC (node blue border). The network nodes represent proteins while the edges represent predicted functional associations. There are 6 types of associations presented: neighborhood (green), co-occurrence (blue), experimental (purple), text mining (yellow), database (light blue), co-expression (black) evidence. The color of the nodes represents cluster membership. Inter-cluster edges are depicted by dashed lines. The colored areas represent functional pathways. HNRNPF = HNRPF.

To further investigate protein–protein interactions within tumor tissue, we performed STRING analysis, taking into account only unique proteins identified in the center vs. margin comparison in SCC and ADC. Even though there was no overlap in proteins between the two subtypes of NSCLC, some proteins were associated with similar pathways in the center vs. margin comparison (Fig 10). Among the top five GO biological processes, leucocyte activation, involved in the immune response (FDR = 5.36E-05) and vesicle-mediated transport (FDR = 7.80E-4), was enriched by 10 (4 SCC/6 ADC) and 13 (8 SCC/5 ADC) proteins, respectively. The second most enriched biological process, the interleukin-12-mediated signaling pathway (FDR = 5.36E-05), was enriched by five proteins (2 SCC/3 ADC).

## Discussion

In this study, we identified, for the first time, several proteins differentially expressed between the tumor center and margin in both ADC and SCC. For some proteins, different proteoforms were also identified. For each lung cancer subtype, a different set of specific proteins was found to be involved in a variety of mechanisms of cancer progression. Therefore, cancer progression in the two lung cancer subtypes may be related to different mechanisms. We also identified several proteins differentially expressed between the control and margin or center of ADC and SCC that could be potential biomarkers for tumors. IPA indicated several canonical pathways and numerous categories related to cancer, including lung cancer. Several proteins detected in our study belonged to “high-abundance” category, including hemoglobin, cytoskeletal, keratins, HSP’s, metabolic and other housekeeping proteins, components of the translational machinery. This likely reflects the limitations of resolving power of 2D-DIGE.

### Proteins related to cancer invasion expressed in ADC tumors

#### Margin vs. center

In this study, we identified several proteins specifically linked to lung cancer invasion and progression. The invasion of cells was also indicated by bioinformatics analysis (see Figs 8 and 9). LMNAs, proteins in the nuclear matrix, were recently associated with lung cancer development and metastasis via epigenetic mechanisms [37]. MSN is a member of the ezrin–radixin–moesin family of proteins involved in various aspects of cell migration, adhesion, and invasion [38, 39], and DPYSL3 and heat-shock chaperonin (HSP60) were linked to lung cancer metastasis [40, 41]. Moreover, CCT was found to be a biomarker of cancer progression and related to the survival of lung cancer patients [42, 43]. A similar role was attributed to ALDH2 for lung and liver cancer patients [44]. The latter has also been linked to chemotherapy resistance [45], whereas TUBB is recognized as a cellular target for chemotherapeutic agents and a marker of centrosome abnormalities in lung cancer [46, 47]. Both TUBB and LGALS1 are involved in podosome assembly, as indicated by GO and STRING analysis. Podosomes are invasive or degenerative structures involved in the degeneration of the extracellular matrix (ECM). Podosomes are found in normal cells as well as cancer cells (named invadosomes) [48, 49]. Subunits of proteasome alpha have been found to participate in the malignant progression of several human cancers, including lung cancer [50], and the inhibition of these subunits was found to be a promising strategy for the radiosensitization of non-small-cell lung cancer [51]. CS, an enzyme of the tricarboxylic acid cycle, has been recognized as an important factor in the progression of cancer pathogenesis and was found to be a marker of chemoresistance in non-small-cell lung carcinoma [27]. Elevated levels of CS were also found in bronchoalveolar lavage fluid from lung adenocarcinoma cancers [52]. CA1 was recognized as a promising biomarker for the early detection of non-small-cell lung cancer in both sera [53] and lung cancer tissues [54, 55]. It is worth to mention that APOA1 was indicated in the recent study [56] to have a prognostic potential to be used in the progression of obstructive pulmonary disease (COPD) to lung cancer.

#### Tumor vs. control comparisons for ADC

The bioinformatic analysis of the margin vs. control proteins clearly supports the role of differentially expressed proteins in the invasion of cells. It is interesting that this analysis provided different sets of proteins than the margin vs. center comparison (with the exception of LMNA; see above). The analysis indicated six upregulated and six downregulated proteins in the margin. These proteins should be targeted for future studies aimed at better understanding ADC tumorigenesis, especially HSP90AB1, which is a potential marker of ADC (see below). It is interesting to note that this analysis did not indicate any predicted activation relationships; rather, relationships related to inhibition (for seven proteins: LMNA, NME1, PKM, S100A4, SERPINA1, VIM, and AHCY) were indicated for both increased and decreased proteins in the tumor (see Fig 9A). These results are currently difficult to interpret but may indicate the complex nature of cell invasion in ADC. Contrary to the analysis of the invasion of cells, the analysis of ADC cellular infiltration clearly indicated the predicted activation of this pathway in ADC for six proteins (ALB, ANXA2, APOA1, PPIA, SERPINA1, and AKR1B1); interestingly, most of the proteins were decreased in the tumor, with the exception of AKR1B1. In addition to APOA1 (see above) also FGB, HP and SERPINA1 were found to be involved in progression from COPD to lung cancer [56]. SERPINA1 was proposed to be useful to monitor evolution of NSCLS reflecting individual cancer progression [57]. In summary, proteins identified in adenocarcinoma patients are associated with several aspects of cancer invasion and progression, including cell migration, adhesion and invasion, cytoskeleton structure, protein folding, and metabolism. This knowledge should provide a foundation for future detailed studies aimed at unraveling the mechanisms of ADC tumorigenesis in detail. In our analysis we have found inconsistent finding with state of downstream molecule (yellow lines present in Fig 9). We would like to emphasize that the inconsistencies were also observed by other scientist when IPA was applied for cancer proteome analysis [58–63].

### Proteins related to cancer invasion expressed in SCC tumors

#### Margin vs. center

Similar to ADC, several proteins specifically linked to cancer invasion and progression were identified, with a wide variety of possible mechanisms. PRDX4 was found to promote human lung cancer progression via the modulation of specific phosphokinase signaling [64]. LDHA in solid tumors was found to be linked to aggressive cancer, which is related to its upregulation due to hypoxia [65]. A recent meta-analysis indicated that high LDH levels might be associated with poor prognosis in lung cancer [66]. Another glycolytic enzyme, PKM, is associated with poor prognosis in non-small-cell lung cancer [67, 68]. Recent studies have indicated a relationship between KRT19 and the aggressive and metastatic dissemination of lung adenocarcinoma [69]. This suggests that KRT19 can be used for the evaluation of both SCC and ADC [70]. A similar relationship was also found for KRT5.

ERO1A is a major regulator of disulfide isomerase, which was found to be a prognostic indicator of NSCLC [71]. EHD1, a member of a family of highly conserved proteins involved in regulating endocytic recycling, is strongly linked to poor survival for lung cancer patients [72–74]. It is possible that the role of EHD1 in tumor progression is related to its role in tumor angiogenesis [75, 76]. RUVBL1, a novel C-RAF-binding protein, was found to promote lung cancer tumorigenesis through the activation of the RAF/MEK/ERK pathway [77]. CTSD, a lysosomal protease, has long been recognized as a SCC biomarker [78, 79]. However, CTSD was also found to be a potential prognostic marker for ADC [80]. ARHGDIB was found to be a suppressor of the migration and invasion of human lung cancer [81, 82]. An interesting finding revealed by STRING analysis indicates vesicle-mediated transport as an important factor in SCC tumorigenesis. Extracellular vesicles play an important role in cancer metastasis via their export to target organs [83]. Exosomes are also recognized as potential regulators of intracellular communication in cancer and are involved in several processes important for cancer progression [84]. As such, they are viewed as part of a series of not linear, but rather concurrent partially overlapped processes critical for metastasis [85]. A progression from COPD to lung cancer was also indicated for TF and for APOA1 [56]. Moreover, exosomal proteins can contain information about tumor identity and most importantly can be involved in regulating of tumor progression and growth [86].

#### Tumor vs. control comparison in SCC

The bioinformatic analysis of the margin vs. control proteins in SCC clearly supports the role of differentially expressed proteins in the invasion of cells. The analysis indicated 17 upregulated and one downregulated protein in the margin. It is interesting to note that, contrary to the case for ADC, this analysis indicated 13 predicted activation relationships and no inhibition at the same time. This strongly points to the importance of the invasion of cells in SCC tumorigenesis. Contrary to the analysis of the invasion of cells, the analysis of SCC cellular infiltration did not indicate the predicted activation of this pathway; however, 13 proteins were indicated as participating in this pathway, mostly via increases in their concentrations (10 proteins).

### Canonical pathways and diseases or functions related to ADC tumor margin and center

The canonical pathway analysis revealed several pathways related to cancer progression. Two canonical pathways related to Rho signaling were identified in our study as being involved in signaling mediated by the Rho GTPase family. Rho GTPases are small GTPases involved in the regulation of the actin cytoskeleton, cell migration, and stem cell differentiation [87]. Consequently, their activity is strongly linked to cancer invasion, including that in lung cancer [88]; their main mechanisms of action are thought to be the formation of dynamic actin-rich protrusions and their role in the turnover of cell–cell and cell–extracellular matrix adhesions [89, 90].

Semaphorins, a family of proteins initially characterized as axon guidance factors, have recently been implicated in several physiological functions, such as immune responses and angiogenesis [91]. Recent evidence suggests that semaphorins are important in the etiology of several forms of cancer [92–94]. There are multiple mechanisms by which semaphorins promote tumor progression, including their effects on angiogenesis and lymphangiogenesis, and direct effects on tumor cells [94]. Recent reports indicate that semaphorins are involved in epithelial–mesenchymal transition (EMT), which is associated with cancer cell heterogeneity, plasticity, and metastasis. Notably, MYL3 and DYPSL3 were upregulated in the tumor center, which strongly suggests the importance of these proteins in the center’s characteristics. It is also interesting to note the association of semaphorins with the RhoA signaling pathway [95, 96]. This signaling pathway is also involved in epithelial adherens junctions (AJs), indicated in our analysis, which are part of the mechanism binding epithelial cells together [97]. It is of interest that both molecules involved in AJs, MYL3 and TUBB, are upregulated in the tumor center, which may be important for AJs in this region. The disturbance of AJs with a loss of epithelial features and gain of mesenchymal phenotype is a typical feature of EMT [98].

The ubiquitin system is conserved in eukaryotes; its main role is the degradation of proteins, but it also plays numerous roles in signal transduction, cell–cell progression, receptor trafficking and endocytosis, and immune responses. The ubiquitination of proteins is a common post-translational modification in most cell types. Disturbances of ubiquitin-mediated processes often result in tumorigenesis and metastasis, including lung cancer [99–101]. Ubiquitination is part of the mechanism leading to the dissociation of AJs during EMT [97]. The upregulation of HSPD1 and PSMA1, the molecules involved in ubiquitination, in the margin suggests that protein turnover is important for ADC progression.

Agranulocyte adhesion and diapedesis belong to the immune/inflammatory-related canonical signaling pathway and are recognized as the primary line of host defense against infection [102]. This pathway has been indicated to be involved in cancer invasion and metastasis in breast and lung cancer [103, 104]. The exact role of agranulocyte adhesion and diapedesis in cancerogenesis is not clear at present; it may be involved in immune-related mechanisms, such as the development of the tumor microenvironment [105], or extracellular matrix remodeling to promote mesenchymal shift and cell transformation [106]. Our results indicate that the molecules involved in this pathway (MSN and MYL3) are either upregulated or downregulated in the margin and center, which suggests the importance of agranulocyte adhesion in both parts of the tumor.

Biogenic amines have been linked to cancer cell growth [107]. Phenylethylamine degradation was indicated as a canonical pathway in our study, and this pathway was shown to be related to drug resistance in various cancer cell lines [108]. However, to our knowledge, phenylethylamine degradation has not yet been linked to lung cancer. Further studies should explore the role of this pathway in lung cancer development.

In summary, we identified several canonical pathways related to tumor progression. These pathways often overlap, indicating their involvement in several specific mechanisms of ADC tumorigenesis. Important physiological phenomena related to cancer invasion were identified, especially including EMT and AJs. Moreover, the functional categories highlight functions related to cell death and survival and cell movement, which indeed are related to cancer invasion. Several proteins were categorized to different cancers, which strongly suggests that they are part of a general mechanism of carcinogenesis. It should be underlined that all signature pathways were predicted by <5 molecules therefore further studies are necessary to validate our results by other analytical approaches, such as IHC, WB, or ELISA.

### Canonical pathways and diseases or functions in SCC tumor margin and center

The canonical pathway analysis of the SCC tumor margin and center revealed pathways related to cancer progression and metabolism. HIF-1α signaling, identified in our study, is involved in the adaptive response of cells to hypoxia through a switch in cell metabolism from aerobic to anaerobic (Warburg effect) [109]. It is worth mentioning that two proteins indicated by this pathway, LDH and TF, were upregulated in the center, whereas a third, PKM, was upregulated in the margin, which may reflect the metabolic specificity of the tumor parts. Hypoxia (a reduced amount of oxygen in the cancer microenvironment, below 10 mmHg of O_2_) is a characteristic feature of several cancers and was found to be linked to a number of features of tumor invasion and metastasis, e.g., EMT, the tumor cell invasion of the basement membrane and ECM, cell motility, angiogenesis and lymphangiogenesis, and extravasation [110]. Overall, these results suggest that a switch in metabolism is an important feature that discriminates the margin and center of SCC tumors. This is supported by the identification of the pyruvate-to-lactate pathway, which is an indication of anaerobic metabolism. It is also interesting that EMC and metastasis has been linked to N-acetylglucosamine (a pathway indicated in this study) via its involvement in post-translational modifications of proteins important for tumorigenesis [111].

Phagosome maturation is related to autophagic processes in tumor cells, which may be related to antitumor action, especially in the earlier stages, or may facilitate tumor progression, especially in the later stages [112]. This agrees with our results indicating the upregulation in the tumor margin of both CTSD and NAPA, which are present in the phagosome maturation pathway. The enrichment of phagosome maturation was observed in NSCLC cells exposed to cigarette smoke [113]. Pathways related to neurodegenerative diseases, such as Huntington’s disease (HD), have been described in metastasis-derived breast cancer cells, which suggests an association between cancer and HD [114]. Our results suggest a similar relationship in lung cancer, which is in agreement with the results of Moreira Sousa et al. [115].

The proper expression of glucocorticoid receptors (GRs) is essential for the normal development of the lung, and the disruption of its expression is linked to cancer development [116]. The tumorigenic action of GR is associated with tumor cell invasion and lung metastasis, leading to EMT induction [117]. On the other hand, GR was also identified as a tumor suppressor gene via regulation mitotic progression [118]. Interestingly, CTSD was also indicated as a molecule related to the biosynthesis of thyroid hormone. A link between lung cancer and thyroid hormone synthesis was indicated decades ago [119]. Its mechanism of action has been linked to the proliferative activity of lung cancer cells via nongenomic action [120]. Moreover, the influence of thyroid hormone drives tumor cell proliferation and survival as well as angiogenesis [121], which links thyroid hormone with HIF-1α signaling indicated by IPA (see above). It is also interesting that thyroid hormone signaling was indicated by microRNA analysis as a pathway enriched in NCLS [122].

### Upstream regulators in ADC and SCC

In our study we employed IPA Upstream Regulator analytic tool which is focused on the identification of the cascade of upstream transcriptional regulators that can explain the observed gene expression changes in our dataset, which can identify the biological activities occurring in the tumor and control tissues. IPA’s definition of upstream transcriptional regulator is quite broad–and include “any molecule that can affect the expression of other molecules”, which means that upstream regulators (or “transcriptional regulators” as they are referred to in this document) can be almost any type of molecule, from transcription factor, to microRNA, kinase, compound, or drug”. Indeed, in our study apart from transcription regulators other compounds were identified as upstream regulators, including proteins (translation regulators, hormone precursors, and enzymes), long non-coding RNAs, several hormonal small molecules, and synthetic or exogenous origin molecules.

#### Transcription regulators

Several upstream regulators were found for center or margin as compared to control both for ADC and SCC. NFE2L2, MYC and MYCN (the later found only for ADC) were identified as activated transcription regulators in our study. The Myc proto-oncogene family (reviewed recently by Wang et al. [123]) consists of three members, C-MYC, MYCN, and MYCL, which encodes the transcription factor c-Myc, N-Myc, and L-Myc, respectively. Diverse mechanisms of aberrant MYC pathway activation in human cancers were identified [124]. MYC transcriptionally activates several hundred target genes that participate in diverse biological processes. Myc oncoproteins are involved in several physiological processes, including cell proliferation, differentiation, survival, and apoptosis. Being the main regulator of cellular oxidative stress, NFE2L2 is redox-sensitive transcription factor which controls the expression of several genes with cytoprotective and antioxidant function, and interestingly can be also controlled by microRNAs [125]. Genes in the KEAP1-NFE2L2 pathway are mutated in ~33% of lung squamous cell carcinoma and ~22% of lung adenocarcinoma [126], which agrees with our results indication the presence of NFE2L2 as activated upstream regulator both in ADC and SCC.

#### Other proteins

Our results also revealed the presence of upstream regulators proteins other than transcription regulators. These proteins belong to several groups, including translation regulators, hormone precursors, and enzymes. LARP1, KDM8 and STK11 were indicated as upstream regulators only in ADC. LARP1 is RNA-binding protein and a member of the LARP family which regulates both mRNA translation and stability and functions as an oncogene in non-small cell lung carcinoma [127]. At a functional level, LARP1 promotes cell migration, invasion, anchorage-independent growth, and in vivo tumorigenesis [128]. It should be underlined however that upstream regulators analysis indicated that LARP1 –mediated pathway is rather inhibited in the center of ADC tumor (negative z-score for center vs. control comparison). Further studies should establish the dynamic of LARP1 upstream regulator in relation to position of cancer cells within the tumor.

KDM8 is involved in epigenetics modifications of genetic information via histone methylation. Several studies have shown that KDMs disorders play an important role in malignant tumors, including lung cancer [129, 130]. Interestingly, KDM8 has been linked to hypoxia-driven epigenetic regulation in cancer progression [131]. STK11 protein plays a role in the metabolism of lipids, glucose, and cholesterol by activating the AMP-activated protein kinase [132]. STK11 is recognized significant gene in cancer which induces tumor heterogeneity, promotes different responses to therapies [133] and is among the most often mutated genes in lung adenocarcinoma [134].

CLPP is located in the mitochondrial matrix. CLPP is an oligomeric serine protease that is similar to the cytoplasmic/nuclear proteasome and plays a central role in mitochondrial protein quality control by degrading misfolded proteins [135]. CLPP is overexpressed in several cancers, including NSCLC [136]. It should be underlined that for CLPP z-score was negative similarly to LARP1 (see above).

#### Long non-coding RNAs

Long non-coding RNAs (lncRNAs) are transcripts without the protein-coding capability with more than 200 nucleotides in length that are mainly generated from gene introns, intragenic regions, promoter regions of coding mRNA, antisense strands of mRNAs and pseudogenes. LncRNA PCGEM1 found in ADC only as activated upstream regulator, plays an important oncogenic role in cancer progression [137] and is widely implicated in a variety of human cancers, including lung cancer [138, 139].

#### Small molecules

*Natural*. The upstream regulators can also be a small molecule that affect gene expression in some direct or indirect way [140]. Our analysis indicated several hormonal small molecules as both inhibited (negative z-score) and activated (positive z-score) upstream regulators. For ADC L-triiodothyronine has been identified as negative upstream regulator. It is well known that thyroid hormone signaling is interrelated with lung cancer in a dual manner, either promoting or inhibitory [141] and our results suggest the latter is true for center of the tumor as compared to the margin. Identification of insulin as upstream regulator in SCC points out well described relationship between diabetes mellitus and cancers, including lung cancer, and hyperinsulinemia and exogenous insulin and insulin analog therapy were indicated as common risk factors for development of cancer [142].

Identification of IL15 as activated upstream regulator for ADC agrees with the knowledge that inflammatory pathways are implicated in lung cancer development [143] and IL15-mediated cross-talk between patrolling monocytes and NK cells is involved in metastasis formation [144]. Testosterone, indicated in our analysis as activated for ADC, has been found to be related to several cancers and its action is related to gender [145]. Moreover, testosterone acts as a precursor for local estrogen production within lung tumors, independent of reproductive organs [146, 147]. Our results also clearly indicated β-estradiol as activated upstream regulator for SCC. Similar to testosterone, estrogen actions were found to contribute to female gender-specific risks in the development of lung carcinoma [148]. The mechanism of estradiol tumorigenesis has been linked to its interaction with estrogen receptors that have been detected in lung cancer cells [149].

*Synthetic or exogenous origin*. We identified small natural-like synthetic compounds, namely biphenyl and terphenyl compounds for ADC, such as: IND S1, IND S7 and MEL S3 [150] that has been pointed out as involved in resistance in cancer cells [151] and 5-fluorouracil is well-known chemotherapy agent [152]. On the other hand, sirolimus and lipopolysaccharide were identified as upstream regulators for SCC. Sirolimus is macrocyclic lactone of bacterial origin and is immunosuppressive agent for considerable improvements in acute and chronic organ rejection and the life expectancy of transplant recipients [153]. Its mechanism is related to the phosphatidylinositol 3-kinase (PI3K)/Akt pathway and reduction of angiogenesis important for cancer development [154]. Lipopolysaccharide‑induced tumor necrosis factor-α factor enhances inflammation and is associated with cancer [155].

Our analysis identified three synthetic retinoids as upstream regulators for SCC, ST1926, tretinoin, and CD437. ST1926 is synthetic retinoid tested for prevention or treatment of certain types of cancer [156, 157]. ST1926 has shown efficacy against several solid tumor models including lung carcinoma [158]. Another synthetic retinoid–tretinoin has also been used advanced in lung cancer studies [159]. CD437 is toxic to numerous cancer cell lines, including NSCLC cancer via induced apoptosis [160, 161]. We have also identified 1,2-dithiole-3-thione, which is organosulfur compound cancer chemopreventive agent. The key mechanism of action of dithiolethiones involves activation of Nrf2 signaling and induction of phase II enzymes [162].

6-OHDA was found as upstream regulator only in SCC. 6-OHDA was found to induce secretion of PARK7/DJ-1 which is a Parkinson disease- and cancer-associated protein that functions as a multifunctional protein involved in gene transcription regulation and anti-oxidative defense [163].

In summary, our analysis revealed several potential upstream regulators of different nature for future studies of the mechanism for ADC and SCC carcinogenesis. Both common and different upstream regulators were identified for both cancer types, for example testosterone for ADC and β-estradiol for SCC. This knowledge can be helpful for better understanding of specificity of ADC and SCC development.

### Differences between tumor margin and center can be explained by a “relay race” model

In our opinion, the results obtained in this study indicating significant differences between the tumor margin and center may be related to differences in the specific metabolisms of these tumor parts. Giatromanolaki et al. [35] proposed the “relay race” model of tumor vascular growth and regression. These authors indicated an extreme difference between the tumor center, with a prevalence of unfavorable environmental conditions, including hypoxia, acidity, and apoptosis (also indicated in our study), accompanied by a decrease in angiogenesis and a shift in metabolism to anaerobic glycolysis. This suggestion is also supported for ADC (but not SCC), in which the activation of necrosis in the tumor center was indicated by IPA. This is the opposite of the case for the tumor margin, where the conditions for growth are favorable for proliferation and angiogenesis. The vascularization of such tumors is known as an “edvin” (“edge vs. inner”) design [35]. The edvin type of tumor vascularization was also attributed to non-small-cell lung cancer [164].

As expected, and as shown in our study, the differences in anatomical and environmental features between the tumor margin and center have to be reflected in differences in biochemical characteristics, including proteins and/or their proteoforms. For example, González et al. [165] demonstrated differences between the invasive margin and breast tumor core in the expression of matrix metalloproteases and their inhibitors on mononuclear inflammatory cells. The results of our study provide detailed information concerning several proteins differentiating the tumor margin and center.

Moreover, comparative IPA indicated pathways that may reflect differences in the microenvironment of either ADC or SCC tumors. For example, canonical pathways (see Fig 8) indicate the importance of glucocorticoid receptor signaling, glycolysis, HIF-1α signaling, the iron homeostasis signaling pathway, and hypoxia signaling in the cardiovascular system for SCC, and aldosterone signaling in epithelial cells, 14-3-3-mediated signaling, and the xenobiotic metabolism AHR signaling pathway for ADC. Disease and function IPA of SCC highlighted activation of morbidity or mortality and the migration of tumor cell lines activation in the tumor center, with the concomitant suppression of cell survival and apoptosis. On the other hand, for the ADC tumor center, the activation of the cell death of tumor lines, necrosis, and apoptosis were indicated, with the concomitant suppression of viral infection and apoptosis of tumor cell lines. The importance of particular pathways is not currently clear, especially in light of the contradictory results; for example, apoptosis in ADC was indicated to be both activated and suppressed in the tumor center. In our opinion, these results open a new area of research focusing on a detailed analysis of biochemical and physiological pathways and the mechanisms related to the dynamics of tumor development. Such detailed studies are also justified by the need for the biochemical profiling of tumor development and identification of potential biomarkers.

### Post-translational modifications

Post-translational modifications are important modifications of protein structures that cannot be predicted from genomic analysis alone but are important for the development of cancer [166]. Several PTMs can be identified using two-dimensional electrophoresis, mainly those resulting in changes in protein mass or charge. Recently, using 2D-DIGE, we identified several proteoforms of blood plasma proteins from lung cancer patients, including proapolipoprotein, apolipoprotein AIV, clusterin, gelsolin, fibrinogen, haptoglobin, hemopexin, transferrin, and serotransferrin [11]. In this study, several proteins with proteoforms were also identified in the tumor margin and center, including lamin, L-plastin, aldehyde dehydrogenase 2, and carbonic anhydrase 1 for ADC and cyclophilin B for SCC. Indeed, in several studies, these proteins were found to be subjected to numerous PTMs. For example, L-plastin phosphorylation influences its actin binding, which was found to be important for cancer invasion, while S-glutathionylation reduces its binding to actin [111, 167]. Oshita et al. [168] found several proteoforms of ALDH2 and suggested that they underwent post-translational modifications such as phosphorylation, acetylation, and glycosylation. These modified forms may be biomarkers that can be used to predict tumor recurrence in patients with early-stage NSCLC. The activity of carbonic anhydrase can be modified via several reactions, such as phosphorylation, disulfide-bridge formation, S-glutathionylation, S-nitrosylation, O- and N-linked glycosylation, non-enzymatic glycation, acetylation, ubiquitination, glycosylphosphatidylinositol anchoring, and methylation, as reviewed by Di Fiore et al. [169]. That study pointed out the significance of PTMs, often detected at conserved amino acids, and noted the need for further studies to unravel the functional importance of these PTMs in both physiological and pathological conditions. The dysregulation of functional networks as a result of PTM variability is clearly recognized as an important factor that needs to be taken into account in the context of biomarker complexity [170]. Our results strongly suggest the need for such an approach in further studies of lung cancer. In our study we used two-dimensional electrophoresis to distinguish protein changes, including those that are likely related to PTMs. We are aware that by using this method we were able to identify only limited number of PTMs as a consequence of restrictions of electrophoresis, namely because only proteins that change their migration on gel (due to changes in protein’s MW and/or charge) could be detected. For example, gene mutations produced by changes in amino acids with similar properties could not be detected in our study and gene and/or protein sequencing are necessary to identify such mutations.

### Comparative analyses between control and tumor tissues

Contrary to the comparison between the margin and center of the lung tumor, comparative analyses between the control and ADC and SCC tumor tissues revealed that, besides repetitions of a few proteins identified in the margin and center, several new proteins differentiating the two tissues were identified. Besides individual proteins, four groups emerged: proteins related to hemoglobin (five proteins), heat-shock proteins (12 proteins), eukaryotic translation elongation factors (six proteins), and keratins (six proteins). Among these groups, both proteins upregulated and downregulated in lung cancer tissues were detected. In the next paragraphs, we discuss their importance in cancer studies.

#### Hemoglobin beta chain

In our previous study, we identified hemoglobin beta chain (HBB) in the blood plasma of lung cancer patients subjected to chemotherapy, which was related to the presence of HBB in erythrocytes [11]. In the current study, four hemoglobins, including alpha and beta chains, were found in lower abundance in both ADC and SCC tissues. These results strongly suggest alternating expression of globin chains in lung cancer cells. This agrees with recent data obtained utilizing single-cell RNA-seq, showing the induction of HBB in breast, prostate, and lung cancer cells [171]. Hemoglobin alpha 1 globin chain (HBA1), identified in our study, was recently shown to be expressed in lung cancer tissues and in the exhaled breath condensate of lung cancer patients [172, 173]. The mechanism of hemoglobin’s action in tumor cells is unknown at present, but it is speculated that HBB can protect tumor cells and their ability to metastasize by controlling ROS inside the cells [174].

#### Heat-shock proteins

The heat shock protein (HSP) family comprises important, highly conserved molecular chaperones involved in the response to several stress conditions, such as heat induction, hypoxia, virus infection, and neoplasia, including lung cancer [175–177]. Among the HSPs identified in our study, we recognized members of the heat shock protein 90 (HSP90) family, including TRA1, HSP90AA1, HSP90AB1, and HSP90B1 [178]; the overexpression of the last has been linked to lung tumor growth, including that in ADC [179–181]. Recently, Klimczak et al. [182] proposed that heat-shock proteins could be used to create signatures for predicting clinical outcomes in breast cancer. Our results clearly demonstrate differences in the occurrence of particular HSPs in ADC and SCC, which suggests that protein signatures based on chaperones may be useful for discriminating ADC and SCC.

#### Eukaryotic translation factors

Eukaryotic translation factors are key proteins in protein synthesis that have been recently identified as a new class of potential oncogenes acting through reducing the fidelity of protein translation, inducing cytoskeleton alterations, and disrupting signaling pathways [183, 184]. Among the elongation factors in this study, we identified EEF1A1, EEF1AL1, EEF1D, EEF1G, and EEF2. EEF1D and EEF1G have been implicated in lung cancer [184, 185]. EEF2 was found to be a novel tumor-associated biomarker that is overexpressed in various cancer types, including lung cancer [186]. EEF1G was found to be included in a 16-gene expression signature for distinguishing stage I from stage II squamous carcinoma of the lung [187]. It is interesting that HSP90AA1, identified in our study (see above), was also included in that signature. Our results reveal that, besides the previously described elongation factors, initiation factors such as EIF3G and EIF-4II are also involved in lung cancer. To date, these factors have only been attributed to other cancers, such as mesothelioma [188] and colorectal cancers [189].

#### Keratins

Besides the differential expression of KRT5 and KRT19 between the margin and tumor center, KRT6B, KRT8, KRT10, and KRT17 were identified among the proteins differentiating the cancer from control tissue. KRT6B and KRT10 were found to be abundant in exhaled breath condensate and to be potential biomarkers for the early diagnosis of lung cancer [172]. The expression of KRT8 was found to correlate with KRT19 and was linked to EMT [190], similar to KRT17 [191]. KRT8 was used for cancer prognosis, including for ADC patients [192, 193], whereas KRT10 was found to be a useful biomarker for the treatment response in lung cancer [194]. Keratins appeared to be subjected to post-translational modifications when they were expressed in tumors [195, 196].

### Proteins upregulated in cancer tissues with high fold changes compared to control

In our study, several proteins of cancer tissues were upregulated with high fold changes compared to control tissues, which makes them possible candidates as tumor biomarkers. The complete list of these proteins is presented in S1 and S2 Tables, and here, we describe potential major biomarkers (selected based on fold changes >5). For ADC, such proteins include HSP90AB1 and HSPD1, which belong to the heat-shock family described above, a major protein group expressed in tumor tissues. Interestingly, the combination of CALR and disulfide isomerase (PDIA3) was found to be a potential prognostic biomarker for non-small-cell lung cancer [197], which coincides with our finding of the upregulation of PDIA4 in ADC and PDIA3 and PDIA4 in SCC tissues.

Similar to ADC, several proteins were attributed to all of the above-mentioned protein groups: heat-shock proteins, eukaryotic translation factors, keratins, and hemoglobin beta chain. Other proteins detected in our study reflect further important functions related to tumorigenesis. For example, ezrin has been linked to cancer invasion and progression as a member of the ezrin–radixin–moesin family of proteins involved in various aspects of cell migration, adhesion, and invasion (see above). In our study, alpha tubulin (TUBA1B) was found to exhibit a high fold change compared to control in SCC samples. It is worth mentioning that beta tubulin was also identified in ADC samples (see above), which strongly supports the universal significance of microtubules for tumorigenesis. Our results suggest potential prognostic value of identified proteins which calls for further studies on larger group of patients and the uses of biostatistical calculations between candidate proteins with various clinicopathological factors.

Tyrosine 3 monooxygenase/tryptophan 5-monooxygenase activation protein zeta (YWHAZ, also named 14-3-3ζ) was identified as a central hub protein for several transduction pathways, with a significant role in tumor progression [198]. YWHAZ is involved in promoting EMT and lung cancer metastasis [199] and was found to be a reliable prognostic biomarker for NSCLS [200]. In addition, 14-3-3 signaling was also indicated by the IPA of our results.

Ingenuity canonical pathways of the ADC and control samples revealed numerous canonical pathways of disease and function for both the margin vs. control and center vs. control comparisons. Some of these categories, such as acute phase response signaling, glycolysis I, and the iron homeostasis signaling pathway, are the same and others differ, which suggests that it is possible to discriminate between markers from different parts of the tumor. Most of these pathways are linked to tumorigenesis and are in agreement with the information discussed above concerning the characterization of the tumor margin and center. A chronic inflammatory-like state is regarded as a hallmark of cancer and is associated with cancer development and disease progression, including that of lung cancer [201]. Glycolysis and HIF-1α signaling (also identified in SCC tumors; see below), which were indicated in our study, are often linked together, because the HIF-1α pathway plays a vital role in tumor cell survival by redirecting glucose metabolism from oxidative phosphorylation to glycolysis [202]. This issue was discussed above.

Contrary to ADC, the IPA of SCC revealed a very high similarity between the tumor center vs. control and margin vs. control comparisons. This difference between the ADC and SCC analyses is difficult to explain; we think that it could stem from differences in the tumor structure and/or the composition of the control samples. Some major canonical pathways were also identified (and described) for SCC, including glucocorticoid receptor signaling, aldosterone signaling in epithelial cells, and PKR acting in interferon induction and antiviral response. The identification of the sirtuin signaling pathway in our study highlights the importance of post-translational modifications in SCC. Interestingly, sirtuin has also been linked to the regulation of pro-tumorigenic exosomes (indicated by STRING analysis; see above) [203].

## Conclusions

Several proteins differentially expressed in the tumor center and tumor margin in ADC and SCC were identified. Proteins differentiating the tumor center and margin were linked to several aspects of cancer invasion and progression, including cell migration, adhesion and invasion, cytoskeletal structure, protein folding, anaerobic metabolism, tumor angiogenesis, EMT, AJs, and inflammatory responses. We identified several new proteins differentiating tumor and control tissues, including ones that showed high fold changes, which makes them possible candidates for tumor biomarkers. This suggestion should be supported in the future by analyzing an independent validation set.

## Materials and methods

### Sample collection

The study was conducted in accordance with the Declaration of Helsinki and approved by the local ethics committee of the Medical University of Bialystok (No. R-I-002/36/20014). Table 7 shows the clinical characteristics of the patients included in this study. Informed consent was obtained from 8 ADC patients (5 men and 3 women; age range, 54–78 years; mean age, 67.2 ± 7.1 years) and 7 SCC patients (4 men and 3 women; age range, 58–81; mean age, 68.4 ± 8.2 years). Tissue specimens from tumor center and margin were collected according to the standard operating procedure of the oncological biobank and the procedure described by Niemira et al. [6]. The details of tissue samples collection in the clinical setting–macroscopic evaluation of resected specimen is presented in S9 Material. In brief, after lung tumor resection, whole specimen was examined macroscopically by the pathologist to determine the exact tumor localization, presence or absence of macroscopic residual tumor, presence or absence of macroscopic infiltration of pulmonary pleura, macroscopic evaluation of possible presence of necrosis in the tumor center. Pathologist cut the exact tissue samples that represent the tumor center and tumor margin. Moreover, pathologist determine the possibility to collect adjacent pulmonary tissue (referred as normal tissue) if the distance from the tumor border was greater than 2 centimeters, pathologist was cutting the samples of adjacent tissue. Then, Study Nurses from Biobank were putting the tissue samples alternately into cryotubes for vapour phase of liquid nitrogen (fresh frozen samples) and into tubes with 10% buffered formalin (formalin-fixed samples).

**Table 7:** Clinical and pathological characteristics of ADC and SCC patients whose samples were included in this study.

Tumor type	Age	Gender	Stage	TNM classification	Smoking habit	Histology of control tissue
ADC	70	F	IIb	pT2b pN1 cM0	Smoker	Focal atelectasis
ADC	64	M	Ib	pT2a pN0 cM0	Ex-smoker	Hemosiderin-laden macrophage deposits, atelectasis
ADC	71	M	Ib	pT2a pN0 cM0	Smoker	Fibrosis, hemosiderin-laden macrophage deposits, atelectasis
ADC	68	F	IIb	pT2b pN1 cM0	Smoker	Focal atelectasis
ADC	54	M	IIIa	pT3 pN0 cM0	Smoker	Fibrosis, hemosiderin-laden macrophage deposits, atelectasis
ADC	78	F	Ib	pT2a pN0 cM0	Smoker	Atelectasis, pneumoconiosis, focal fibrosis
ADC	70	M	Ia3	pT1c pN0 cM0	Smoker	Fibrosis, atelectasis
ADC	63	M	IIIa	pT4 pN0 cM0	Smoker	Emphysema, pneumoconiosis
SCC	71	F	IV	pT2b pN0 pM1a	Ex-smoker	Fibrosis, atelectasis, pneumoconiosis, inflammatory cell infiltration consisted of lymphocytes
SCC	81	M	IIa	pT2b pN0 cM0	Ex-smoker	Emphysema, pneumoconiosis, congestion
SCC	63	F	IIa	pT2b pN0 cM0	Ex-smoker	Atelectasis, pneumoconiosis
SCC	68	M	IIIa	pT2a pN2 cM0	Ex-smoker	Focal fibrosis, emphysema, congestion
SCC	76	M	IIa	Not determined	Ex-smoker	Focal edema, inflammatory cell infiltration, atelectasis
SCC	58	M	IIb	pT2a pN1 cM0	Smoker	Hemosiderin-laden macrophage deposits, atelectasis, emphysema, congestion and fibrosis
SCC	62	F	IIb	pT3 pN0 cM0	Smoker	Emphysema, pneumoconiosis, congestion

Histopathological analyses were performed once more by a physician with pathomorphological expertise to confirm the diagnosis of ADC or SCC or the presence of non-malignant lung tissue. The details of microscopic evaluation of fresh frozen and formalin-fixed paraffin-embedded (FFPE) tissue samples is presented in S9 Material. Quality control and microscopic evaluation were performed for fresh frozen tissue samples and the corresponding FFPE samples. The whole procedure of microscopic quality control of hematoxylin and eosin (H&E) stained tissue sections prepared from fresh frozen tissue samples and FFPE samples was performed by a pathologist, and tissue section preparation was done by the laboratory staff from the Pathology Department. In brief, the preparation of frozen sections were performed from tissues frozen in liquid nitrogen in a cryostat, where cryotissue sections were cut at 5 μm. The microscopic quality assessment of H&E-stained tissue section included following attributes: i) confirmation of the specified organ, ii) histopathological diagnosis compatible with the present H&E section, iii) percentage of tumor content: the epithelial part of the tumor (no tumor stroma), iv) percentage of necrosis (%), v) severity of presence of acellular substance, vi) severity of inflammation, vii) severity of fibrosis, viii) severity of hemorrhage. Following tissue characteristics: acellular substance, inflammation, fibrosis, and hemorrhage were classified according to severity based on a number system (low– 1, medium– 2, high– 3).

For adjacent normal tissue samples (referred as normal tissue), pathologist was ensuring that they contain a representative proportion of organ-specific epithelium or tissue. Normal tissue samples that contained a tumor cells were evaluated as a tumor sample depending on tumor content and tissue quality. The adjacent normal tissue (control tissue) was collected 3–5 cm from the tumor.

**Fig 11:**
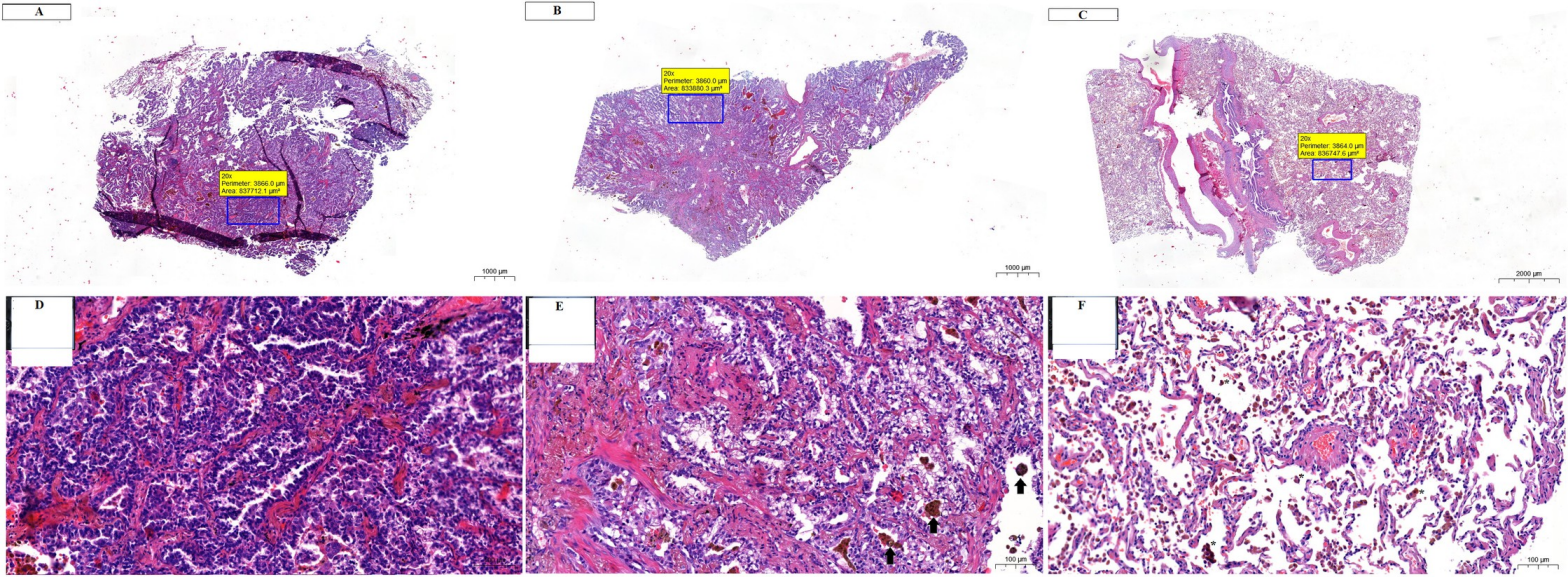
Microscopic features of lung adenocarcinoma (figures A to B and D to E) following hematoxylin and eosin staining. (A) Whole slide image of tissue collected from the margin of tumor diagnosed as pulmonary adenocarcinoma. (B) Whole slide image of tissue collected from the center of tumor diagnosed as pulmonary adenocarcinoma. (C) Whole slide image of tissue collected from non-tumor tissue referred as normal tissue. (D) Region of interest: magnification 20x. Lung adenocarcinoma with mixed subtypes–sample collected from tumor margin. A tumor content of 90% was determined by the pathologist and can be seen in the slide scan. (E) Region of interest: magnification 20x. Lung adenocarcinoma with mixed subtypes–sample collected from tumor center. A tumor content of 90% was determined by the pathologist and can be seen in the slide scan. Dust-laden macrophages were marked with solid arrows. (F) Region of interest: magnification 20x. Lung parenchyma–sample collected from non-tumor tissue referred as normal tissue. Numerous hemosiderin-laden macrophages can be seen in this sample and the examples of these cells are marked with asterisks.

**Fig 12:**
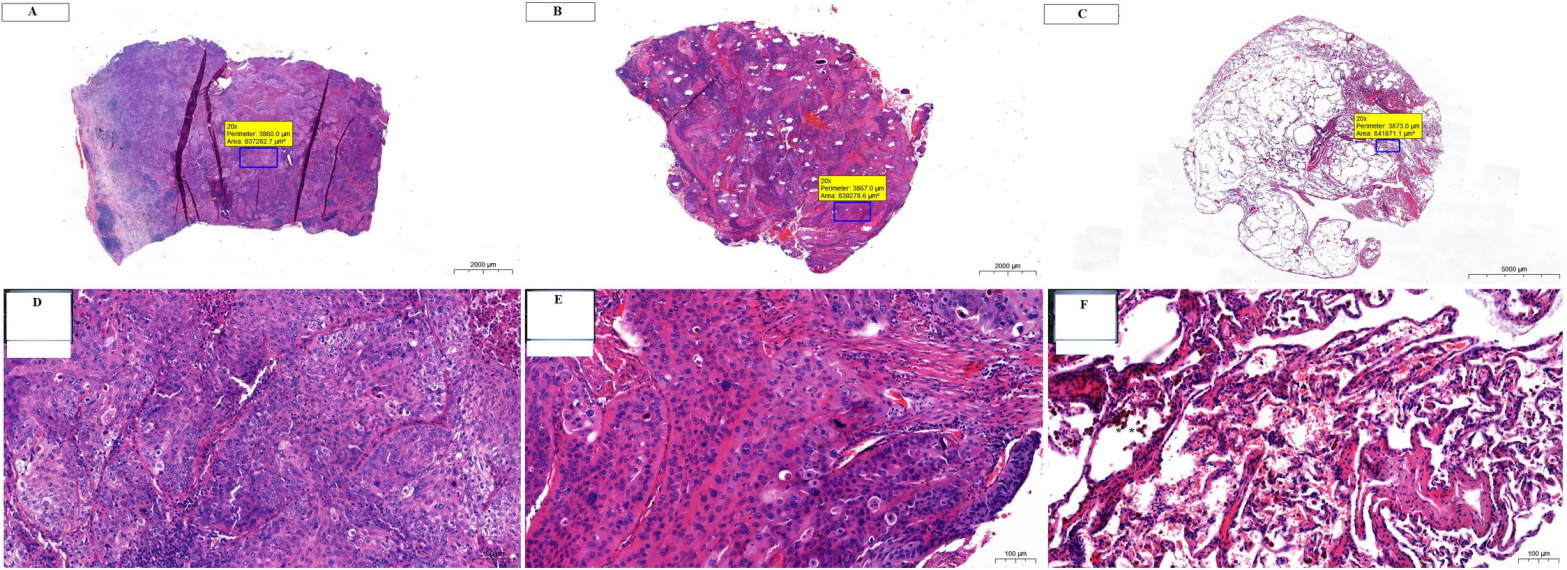
Microscopic features of lung squamous cell carcinoma (figures A to B and D to E) following hematoxylin and eosin staining. (A) Whole slide image of tissue collected from the margin of tumor diagnosed as pulmonary adenocarcinoma. (B) Whole slide image of tissue collected from the center of tumor diagnosed as pulmonary adenocarcinoma. (C) Whole slide image of tissue collected from non-tumor tissue referred as normal tissue. (D) Region of interest: magnification 20x. Squamous cell carcinoma, large cell, non-keratinizing, tumor grade G2 –moderately differentiated–sample collected from tumor margin. A tumor content of 80% was determined by the pathologist and can be seen in the slide scan. Tumor cells are visible in this section part of the slide. The presence of tumor necrosis was determined by a pathologist during microscopic evaluation in 2% of the tissue section area. (E) Region of interest: magnification 20x. Squamous cell carcinoma, large cell, non-keratinizing, tumor grade G2 –moderately differentiated–sample collected from tumor center. A tumor content of 80% was determined by the pathologist and can be seen in the slide scan. The presence of tumor necrosis was determined by a pathologist during microscopic evaluation in 2% of the tissue section area. (F) Region of interest: magnification 20x. Lung parenchyma–sample collected from non-tumor tissue referred as normal tissue. A small number of hemosiderin-laden macrophages can be seen in this sample and these cells are marked with asterisks.

Criteria used to distinguish the tumor margin during microscopic evaluation by pathologist included determination of invasive margin and borderline between normal and tumor tissue. Every tissue sample collected from tumor center and tumor margin defined so in the macroscopic evaluation after tumor resection, were examined in the context of intratumoral morphological heterogeneity. Since the width of the peritumoral area was not defined clearly in official standards, in this study and for routine examination of biobanked samples, the tumor margin was characterized within 1 μm. The cancer samples that were verified histochemically contained tumor cell contents of 72.5 ± 15.8 and 71.2 ± 15.8% for the ADC margin and center, respectively, and 80.0 ± 5.8 and 80.0 ± 8.2% for the SCC margin and center, respectively. No tumor cells were found in normal tissue samples; however, several pathological conditions were identified (Table 7). The representative histological pictures of cancer and control tissues were shown in Figs 11 and 12.

### Protein extraction

Proteins were extracted from lung tissue according to a previously described method [5] with some modifications. In brief, fragments of normal tissue and tissues of the tumor center and margin (~100 mg) were sonicated using a VC-13 PB (Sonics, Newtown, CT, USA) set at 35% relative output, for 30 s in 100 μL of extraction buffer (7 M urea, 2 M thiourea, 30 mM Tris, 4% 3-([3-cholamidopropyl] dimethylammonio) 1-propanesulfonate (CHAPS), 100 mM dithiothreitol, 2% (v/v) immobilized pH gradient (IPG) buffer (3−10 NL), 1% (v/v) Triton X-100) containing 1% (v/v) protease inhibitor cocktail (Sigma-Aldrich, St. Louis, MO, USA). After sonication, tissue extracts were kept on ice for 1 h and then centrifuged at 12,800 ×g for 20 min at 4°C, and 200 μg of protein was precipitated using a 2-D Clean-up Kit (GE Healthcare, Uppsala, Sweden). The precipitate was dissolved in labeling buffer (7 M urea, 2 M thiourea, 4% w/v CHAPS, and 30 mM Tris; pH 8.0). The protein concentrations before and after precipitation were measured using a Coomassie Plus Kit (Thermo Fisher Scientific, Waltham, MA, USA) with bovine serum albumin as the standard.

### 2D-DIGE analysis of lung tissue from ADC and SCC patients

Two independent 2D-DIGE analyses were performed to compare the protein profiles of the normal lung tissue and tissues of the tumor center and margin collected from the ADC patients (n = 8 for each group) and SCC patients (n = 7 for each group). Protein labelling with CyDye DIGE fluor and 2D electrophoresis was performed as previously described by Ciereszko et al. [11]. Aliquots of 50 μg of protein from each sample (normal tissue and tissues of tumor center and margin) were labelled with CyDye DIGE Fluor minimal dyes (GE Healthcare) at a concentration of 400 pmol dye/50 μg of protein according to the scheme presented in S3 and S4 Tables. A dye swap (Cy3/Cy5) was performed between the samples of the control and tumor tissues to exclude dye bias. The internal standard was generated by combining equal amounts of each sample within the experiment and was labelled with Cy2. Differentially labelled samples were mixed together according to the scheme in S3 and S4 Tables and loaded onto Immobiline DryStrip gel strips (24 cm, pH 3 to 10 non-linear; GE Healthcare). Isoelectric focusing was performed with an IPGphor isoelectric focusing unit (GE Healthcare), and SDS-PAGE was run using the ETTAN Dalt six electrophoresis unit (GE Healthcare) as previously described [11].

### Image acquisition and quantitative analysis

The CyDye-labelled gels were scanned using a Typhoon FLA 9500 instrument (GE Healthcare). After the multiplexed images were acquired, image analysis was performed with the use of DeCyder differential analysis software (version 5.0; GE Healthcare). Intragel spot detection and quantification and intergel matching and quantification were performed using differential in-gel analysis. During spot detection, the estimated number of spots was set at 10,000, and the volume, <30,000. Only spots that were successfully matched on >80% of the gel images were considered. To properly select and identify the spots, the DIGE gels were stained using CBB-G250 (Bio-Rad, Hercules, CA, USA), followed by spot excision and identification using matrix-assisted laser desorption/ionization time-of-flight/time-of-flight (MALDI TOF/TOF) mass spectrometry (MS; Bruker Daltonics, Bremen, Germany).

### MALDI TOF/TOF protein identification

Spots of interests were cut from the gel and subjected to reduction, alkylation, and in-gel trypsin digestion as described by Ciereszko et al. [11]. The peptides were concentrated and desalted using Zip-Tip pipette tips (Sigma-Aldrich). Finally peptides were eluted with 1 μL of matrix solution (5 mg of α-cyano-4-hydroxycinnamic acid (CHCA, Bruker Daltonics) in 1 ml of 50% acetonitrile and 0.1% trifluoroacetic acid and spotted directly on a steel MALDI target plate (MT 34 Target Plate Ground Steel, Bruker Daltonics). Additionally, peptide calibration standard (Bruker Daltonics) was spotted following the dried-droplet method with the CHCA matrix for the calibration of mass spectrometer. Peptide calibration standard was composed of a mixture of following peptides with monoisotopic [M+H]+ m/z values: bradykinin 1–7 [757.3992], angiotensin II [1046.5418], angiotensin I [1296.6848], substance P [1347.7354], bombesin [1619.8223], ACTH clip 1–17 [2093.0862], ACTH clip 18–39 [2465.1983] and somatostatin 28 [3147.4710].

Mass spectra were acquired in the range of 500–3500 m/z, using an MALDI TOF AutoFlex Speed TOF/TOF mass spectrometer equipped with a Smartbeam II laser (355 nm, Bruker Daltonics). Operating conditions were as follows: laser frequency = 1000.0 Hz, ion source 1 = 19.10 kV, ion source 2 = 16.80 kV, lens voltage = 7.50 kV, reflector voltage = 20.99 kV, reflector 2 voltage = 9.59 kV, optimised pulsed ion extraction time = 120 ns, matrix suppression = 500 Da and positive reflectron mode was used. The strongest precursors were selected for MS/MS analysis with the following operating conditions: detection range = 40–2285 Da, laser frequency = 200.0 Hz, ion source 1 = 6.04 kV, ion source 2 = 5.34 kV, lens voltage = 3.00 kV, reflector voltage = 26.99 kV, reflector 2 voltage = 11.59 kV, lift 1 voltage = 18.96 kV, lift 2 voltage = 4.00 kV, optimised pulsed ion extraction time = 130 ns and positive reflectron mode was used. The MS peptide mass fingerprint (PMF) and fragment mass spectra (MS/MS) from each individual spot were combined and used to search against the National Center for Biotechnology Information *Homo sapiens* database (searched on December 4, 2019) using the Mascot Server (Matrix Science, London, UK) with the following settings: cleavage enzyme, trypsin; max missed cleavages, 2; fragment ion mass tolerance, 0.5 Da; parent ion mass tolerance, 100 ppm; alkylation of cysteine by carbamidomethylation as a fixed modification; and oxidation of methionine as a variable modification. The protein was considered identified if it met the following criteria: 1) data sets were thresholded below 1% protein FDR and 2) the minimum threshold to two uniquely mapping peptides according to Omenn et al. [204]. The mass spectrometry proteomics data have been deposited to the ProteomeXchange Consortium via the PRIDE [205] partner repository with the dataset identifier PXD032736 and PXD032962 for ADC and SCC, respectively.

### Verification of 2D-DIGE results by Western blotting

Western blotting was used to verify the results obtained in the proteomic study. We used the V3 stain-free workflow, which eliminates the need for stripping and reprobing the blot for housekeeping proteins [206]. The expression of three proteins of interest was evaluated in the margin and center of the ADC tumor (plastin, LCP1, lamin, LMNA, and mitochondrial aldehyde dehydrogenase 2, ALDH2) as well as the SCC tumor (cytokeratin 19, KRT19, pyruvate kinase, PKM, and Rho GDP-dissociation inhibitor 2, ARHGDIB). The Western blot was performed as previously described [207] with some modifications. Equal amounts of protein (30 μg) were fractionated on 12% Criterion™ TGX Stain-Free™ Protein Gels (Bio-Rad). After electrophoresis, the gels were activated on a Chemidoc according to the manufacturer’s instructions (Bio-Rad); then, the proteins were transferred to PVDF membranes using a Mini Trans–Biol Cell (Bio-Rad) at 60 V for 90 min. After transfer, a stain-free image of the PVDF membranes for total protein normalization was obtained before the membranes were rinsed briefly in distilled water and blocked with 5% bovine serum albumin (Sigma-Aldrich) and then incubated with primary polyclonal antibodies (Abcam, Cambridge, UK) against LCP1 (1:1000), LMNA (1:500), ALDH2 (1:500), KRT19 (1:500), PKM (1:1000), and ARHGDIB (1:300) overnight at 4°C. After rinsing the membrane to remove unbound primary antibodies, it was exposed to goat anti-rabbit antibodies (1:5000; Sigma-Aldrich) linked to alkaline phosphatase. The products were visualized by incubation in a solution of alkaline phosphate buffer with the addition of nitro blue tetrazolium (Sigma-Aldrich) and 5-bromo-4-chloro-3-indolyl phosphate (Sigma-Aldrich) in the dark. Antibody-bound proteins were detected by enhanced chemiluminescence using the Chemidoc imaging system (Bio-Rad). All the band intensities were measured with the Image Lab software version 5.2 (Bio-Rad). The image of the gel acquired before transfer was used as a control for unequal protein loading among the samples. The volume density of each target protein band was normalized to its respective total protein content, whereas the total protein band was normalized to the total protein loaded into each lane using stain-free technology, with the data expressed in arbitrary units.

### Functional analysis

Ingenuity pathway analysis (IPA) software (IPA®, Qiagen, Redwood City, CA, USA) was used to investigate the functional and canonical pathways enriched by the differentially expressed proteins (http://www.ingenuity.com) and to predict upstream regulators. Fisher’s exact test and Benjamini–Hochberg multiple testing corrections were used to calculate statistical significance (p < 0.05). Protein–protein interaction network and protein–set enrichment analysis were performed using the STRING 11.0 software [208]. The minimum interaction confidence score for the interaction sources was set to 0.7 for all the networks except the center vs. margin comparison for SCC and ADC due to the short gene lists. The interaction confidence for the last two comparisons was set to 0.4. The MCL clustering algorithm was used, with the inflation set to 3.

### Statistical analysis

Statistical analysis of the changes in protein abundance was performed using the Biological Variance module of the DeCyder Differential Analysis software version 5.02 (GE Healthcare) on 8 and 7 biological replicates corresponding to individual ADC and SCC patients, respectively. Direct comparisons of spot volumes were made between Cy3- or Cy5-labelled samples and the Cy2-labelled pool standard for each gel. The Cy3/Cy2 and Cy5/Cy2 ratios were used to calculate the average changes in abundance. The data were expressed as the log standardized abundance to ensure that the data were normally distributed. One-way ANOVA, t-tests, and average ratio tests were performed; changes in protein spot abundance were considered statistically significant at p < 0.05. For the MS PMF and MS/MS ion search, statistically significant (p ≤ 0.05) matches found by MASCOT were regarded as correct hits. The differences in the expression of verified proteins between the margin and center were analyzed by Student’s t-tests using the GraphPad Prism 5 statistical software (GraphPad Software Inc., San Diego, CA, USA). The data are presented as the means ± SDs.

## Supporting information

S1 Table Proteins found to be present in different abundances in the lung cancer of center and margin of ADC tumor in relation to control. (DOCX)

S2 Table Proteins found to be present in different abundances in the lung cancer of center and margin of SCC tumor in relation to control. (DOCX)

S3 Table Mixing and dying scheme of ADC lung cancer samples, n = 8 for each group. (DOCX)

S4 Table Mixing and dying scheme of SCC lung cancer samples, n = 7 for each group. (DOCX)

S1 Material IPA of ADC. (XLSX)

S2 Material IPA of SCC. (XLSX)

S3 Material Enrichment of ADC (center vs. control). (XLSX)

S4 Material Enrichment of ADC (center vs. margin). (XLSX)

S5 Material Enrichment of ADC (margin vs. control). (XLSX)

S6 Material Enrichment of SCC (center vs. control). (XLSX)

S7 Material Enrichment of SCC (center vs. margin). (XLSX)

S8 Material Enrichment of SCC (margin vs. control). (XLS)

S9 Material Summary of standard operating procedure regulating the process of tissue samples collection in the clinical setting–macroscopic evaluation of resected specimen and the process of microscopic evaluation of fresh frozen and formalin-fixed paraffin-embedded tissue samples. (DOCX)

S1 Raw images Uncropped, unadjusted images of western blots. (PDF)
