# Supplementary material for: Application of two-dimensional difference gel electrophoresis to identify protein changes between center, margin, and adjacent non-tumor tissues obtained from non-small-cell lung cancer with adenocarcinoma or squamous cell carcinoma subtype
Source: PLoS One. 2022 May 5;17(5):e0268073. doi: 10.1371/journal.pone.0268073 (PMC9071164; doi:10.1371/journal.pone.0268073)
Supplement: S1 Table — (DOCX) [file pone.0268073.s001.docx]

| **S1 Table.** Proteins found to be present in different abundances in the lung cancer of center and margin of ADC tumor in relation to control. | | | | | | | | | | | | | | |
| --- | --- | --- | --- | --- | --- | --- | --- | --- | --- | --- | --- | --- | --- | --- |
| **Spot no in Fig. 1a-b** | ***Protein name*** | **Gene name** | **Accession**  **number** | **Calculated**  **MW/pI** | **Protein score** | **Sequence coverage%** | **No of unique peptides** | **Precursor mass** | | **Peptide score** | **Peptide sequence** | **Centrum vs Margin** | **Centrum vs Control** | **Margin vs Control** |
|  |  |  |  |  |  |  |  | **Observed** | **Theoretical** |  |  |  |  |  |
| 391 | *Staphylococcal nuclease domain-containing protein 1 [Homo sapiens]* | SND1 | NP_055205 | 102618/6.74 | 308 | 21 | 4 | 1429.6999  1709.8240  1071.4967  1245.5090 | 1428.6885  1708.8169  1070.4821  1244.4946 | 32  81  29  32 | R.SEAVVEYVFSGSR.L  R.NDIASHPPVEGSYAPR.R  K.FVDGEWYR.A  R.ADDADEFGYSR.- |  | 1.58 | 1.63 |
| 495 | *elongation factor 2 [Homo sapiens]* | EEF2 | NP_001952 | 96246/6.41 | 389 | 26 | 6 | 1091.5814  1402.7947  1543.7714  2143.0523  1138.5156  1977.9450  1799.8876 | 1090.5771  1401.7980  1542.7678  2142.0705  1137.5091  1976.9666  1798.8890 | 34  14  17  32  38  75  15 | M.VNFTVDQIR.A  K.KEDLYLKPIQR.T  K.ARPFPDGLAEDIDK.G  K.ARPFPDGLAEDIDKGEVSAR.Q  K.YEWDVAEAR.K  R.GHVFEESQVAGTPMFVVK.A + Oxidation (M)  K.AYLPVNESFGFTADLR.S |  | 2.59 | 2.69 |
| 499 | *elongation factor 2 [Homo sapiens]* | EEF2 | NP_001952 | 96246/6.41 | 283 | 28 | 5 | 1091.5974  1402.8118  1274.7210  1138.5315  1308.6593  760.3445 | 1090.5771  1401.7980  1273.7030  1137.5091  1307.6510  759.3704 | 18  16  23  20  21  5 | M.VNFTVDQIR.A  K.KEDLYLKPIQR.T  K.EDLYLKPIQR.T  K.YEWDVAEAR.K  K.DSVVAGFQWATK.E  R.FYAFGR.V |  | 4.01 | 3.99 |
| 500 | *elongation factor 2 [Homo sapiens]* | EEF2 | NP_001952 | 96246/6.41 | 478 | 32 | 9 | 2576.2567  1494.7781  1402.7912  1543.7566  2143.0367  1138.5035  1208.4916  1378.6989  1799.8792 | 2575.2986  1493.7952  1401.7980  1542.7678  2142.0705  1137.5091  1207.4961  1377.7075  1798.8890 | 19  44  35  21  37  59  23  22  34 | R.VTDGALVVVDCVSGVCVQTETVLR.Q  R.TFCQLILDPIFK.V  K.KEDLYLKPIQR.T  K.ARPFPDGLAEDIDK.G  K.ARPFPDGLAEDIDKGEVSAR.Q  K.YEWDVAEAR.K  K.EGALCEENMR.G  R.CLYASVLTAQPR.L  K.AYLPVNESFGFTADLR.S |  | 3.99 | 4.03 |
| 505 | *elongation factor 2 [Homo sapiens]* | EEF2 | NP_001952 | 96246/6.41 | 450 | 28 | 7 | 1091.5654  1402.7800  1615.7450  890.4995  2143.0403  1138.4958  1378.6950  1799.8722 | 1090.5771  1401.7980  1614.7573  889.5022  2142.0705  1137.5091  1377.7075  1798.8890 | 46  32  26  27  32  55  29  20 | M.VNFTVDQIR.A  K.KEDLYLKPIQR.T  K.TGTITTFEHAHNMR.V  K.FSVSPVVR.V  K.ARPFPDGLAEDIDKGEVSAR.Q  K.YEWDVAEAR.K  R.CLYASVLTAQPR.L  K.AYLPVNESFGFTADLR.S |  | 3.07 | 3.09 |
| 593 | *lamin isoform A [Homo sapiens]* | LMNA | NP_733821 | 74322/6.73 | 495 | 42 | 7 | 1089.5375  1182.5910  1752.8374  1430.7494  1331.6577  1491.7167  1566.7258 | 1088.5462  1181.6040  1751.8550  1429.7776  1330.6703  1490.7399  1565.7434 | 41  39  51  88  24  27  36 | R.SLETENAGLR.L  R.TLEGELHDLR.G  R.NSNLVGAAHEELQQSR.I  R.IDSLSAQLSQLQK.Q  K.LALDMEIHAYR.K  R.TALINSTGEEVAMR.K  R.SVGGSGGGSFGDNLVTR.S | 2.08 |  | -1.82 |
| 594 | *lamin isoform A [Homo sapiens]* | LMNA | NP_733821 | 74380/6.57 | 387 | 35 | 5 | 1182.6189  1752.8662  1893.9255  1331.6835  1566.7614 | 1181.6040  1751.8550  1892.9189  1330.6703  1565.7434 | 35  53  106  23  15 | R.TLEGELHDLR.G  R.NSNLVGAAHEELQQSR.I  R.MQQQLDEYQELLDIK.L  K.LALDMEIHAYR.K  R.SVGGSGGGSFGDNLVTR.S | 1.42 |  |  |
| 596 | *heat shock protein HSP 90-beta isoform c [Homo sapiens]* | HSP90AB1 | NP_001258901 | 82611/4.98 | 499 | 26 | 5 | 1194.6136  2255.9214  1311.5452  1847.7686  829.5215  1513.7577  1249.5915  1782.9250 | 1193.6404  2254.9516  1310.5626  1846.7897  828.5221  1512.7784  1248.6098  1781.9424 | 51  25  30  122  27  49  25  10 | K.IDIIPNPQER.T  K.HNDDEQYAWESSAGGSFTVR.A  K.EDQTEYLEER.R  R.NPDDITQEEYGEFYK.S  R.ALLFIPR.R  R.GVVDSEDLPLNISR.E  K.EQVANSAFVER.V  K.HLEINPDHPIVETLR.Q |  | 9.41 | 7.36 |
| 632 | *lipoma-preferred partner isoform X1 [Homo sapiens]* | LPP | XP_011511125 | 69829/6.79 | 282 | 34 | 4 | 1643.7065  1947.8207  1553.7486  1133.4762 | 1642.7052  1946.8179  1552.7555  1132.4873 | 81  40  46  20 | R.YYEGYYAAGPGYGGR.N  K.MLYDMENPPADEYFGR.C  K.EPIMPAPGQEETVR.I  R.DFHVHCYR.C |  | 2.16 |  |
| 710 | *protein disulfide-isomerase A4 isoform 1 precursor [Homo sapiens]* | PDIA4 | NP_001358173 | 73357/4.96 | 713 | 37 | 10 | 1126.5554  1209.5647  1081.4787  2039.8810  1317.6322  1442.7543  1458.7401  1627.7803  1755.8719  1502.7005 | 1125.5706  1208.5786  1080.4836  2038.8980  1316.6513  1441.7599  1457.7548  1626.7930  1754.8879  1501.7161 | 64  29  20  133  66  70  54  51  32  24 | R.FDVSGYPTIK.I  K.KGQAVDYEGSR.T  K.GQAVDYEGSR.T  K.GESDPAYQQYQDAANNLR.E  K.FHHTFSTEIAK.F  K.VSQGQLVVMQPEK.F  K.VSQGQLVVMQPEK.F + Oxidation (M)  K.VEGFPTIYFAPSGDK.K  K.VEGFPTIYFAPSGDKK.N  K.FEGGDRDLEHLSK.F |  | 3.18 | 2.59 |
| 757 | *moesin isoform X1 [Homo sapiens]* | MSN | XP_011529261 | 71434/5.83 | 463 | 30 | 7 | 1660.7805  2362.1356  1104.5759  1310.6793  1182.5883  1831.9406  3312.3557 | 1659.7933  2361.1601  1103.5764  1309.6819  1181.5869  1830.9509  3311.4120 | 70  36  67  30  73  23  52 | R.EVWFFGLQYQDTK.G  K.GSELWLGVDALGLNIYEQNDR.L  K.IGFPWSEIR.N  K.KAPDFVFYAPR.L  K.APDFVFYAPR.L  K.KTQEQLALEMAELTAR.I  K.TAMSTPHVAEPAENEQDEQDENGAEASADLR.A | -1.11 |  |  |
| 798 | *Vimentin isoform X1 [Homo sapiens]* | VIM | XP_006717563 | 53676/5.06 | 603 | 39 | 6 | 1115.5635  1254.5606  1323.6150  1533.8404  1093.5222  1570.8920  1668.8532 | 1114.5618  1253.5598  1322.6102  1532.8450  1092.5200  1569.8878  1667.8366 | 23  55  70  122  43  100  40 | K.VELQELNDR.F  R.LGDLYEEEMR.E  R.EEAENTLQSFR.Q  R.KVESLQEEIAFLK.K  K.FADLSEAANR.N  R.ISLPLPNFSSLNLR.E  R.ETNLDSLPLVDTHSK.R |  |  | -1.51 |
| 818 | *albumin, partial [Homo sapiens]* | ALB | AEE60908 | 68484/5.73 | 219 | 37 | 5 | 2086.8857  1467.8912  1311.7726  960.5928  1342.6847 | 2085.8303  1466.8358  1310.7347  959.5552  1341.6275 | 28  28  10  26  16 | K.VHTECCHGDLLECADDR.A  R.RHPDYSVVLLLR.L  R.HPDYSVVLLLR.L  K.FQNALLVR.Y  K.AVMDDFAAFVEK.C |  |  | 1.56 |
| 823 | *heat shock cognate 71 kDa protein isoform X1 [Homo sapiens]* | HSPA1A | XP_011541100 | 71082/5.37 | 280 | 20 | 4 | 1228.6653  1981.9801  1691.7326  1253.6205 | 1227.6207  1980.9905  1690.7183  1252.6088 | 12  59  71  73 | K.VEIIANDQGNR.T  K.TVTNAVVTVPAYFNDSQR.Q  K.STAGDTHLGGEDFDNR.M  R.FEELNADLFR.G |  |  | 2.07 |
| 900 | *plastin-2 isoform X1 [Homo sapiens]* | LCP1 | XP_005266431 | 70814/5.29 | 503 | 36 | 7 | 1154.5873  1069.6277  1576.6842  1743.8589  1287.6142  1675.8338  1799.9231 | 1153.5920  1068.6179  1575.6888  1742.8509  1286.6190  1674.8326  1798.9214 | 33  26  29  86  40  45  44 | K.YAFVNWINK.A  K.LSPEELLLR.W  R.WANYHLENAGCNK.I  K.GDEEGVPAVVIDMSGLR.E  R.NWMNSLGVNPR.V  K.FSLVGIGGQDLNEGNR.T  K.VNDDIIVNWVNETLR.E | -1.32 | -1.55 |  |
| 911 | *plastin-2 isoform X1 [Homo sapiens]* | LCP1 | XP_005266431 | 70814/5.29 | 695 | 46 | 11 | 1877.8414  1502.7444  1069.6110  1576.6907  1431.7485  1743.8432  1405.7793  1287.6138  1675.8296  1458.8378  1799.9150 | 1876.8473  1501.7446  1068.6179  1575.6888  1430.7558  1742.8509  1404.7877  1286.6190  1674.8326  1457.8428  1798.9214 | 32  31  34  19  49  78  52  21  70  13  82 | R.EITENLMATGDLDQDGR.I  K.MINLSVPDTIDER.T  K.LSPEELLLR.W  R.WANYHLENAGCNK.I  K.AYYHLLEQVAPK.G  K.GDEEGVPAVVIDMSGLR.E  K.LNLAFIANLFNR.Y  R.NWMNSLGVNPR.V  K.FSLVGIGGQDLNEGNR.T  R.TLTLALIWQLMR.R  K.VNDDIIVNWVNETLR.E | -1.33 |  |  |
| 916 | *plastin-2 isoform X1 [Homo sapiens]* | LCP1 | XP_005266431 | 70814/5.29 | 965 | 59 | 9 | 1877.8707  1502.7651  1431.7605  1743.8714  1405.8030  1287.6366  1675.8521  1458.8629  1799.9371 | 1876.8473  1501.7446  1430.7558  1742.8509  1404.7877  1286.6190  1674.8326  1457.8428  1798.9214 | 80  67  58  134  48  55  86  22  94 | R.EITENLMATGDLDQDGR.I  K.MINLSVPDTIDER.T  K.AYYHLLEQVAPK.G  K.GDEEGVPAVVIDMSGLR.E  K.LNLAFIANLFNR.Y  R.NWMNSLGVNPR.V  K.FSLVGIGGQDLNEGNR.T  R.TLTLALIWQLMR.R  K.VNDDIIVNWVNETLR.E | -1.29 |  |  |
| 991 | *bifunctional purine biosynthesis protein ATIC [Homo sapiens]* | ATIC | NP_004035 | 65089/6.27 | 183 | 24 | 2 | 1355.7691  2034.8872 | 1354.7833  2033.9119 | 35  38 | K.TLHPAVHAGILAR.N  K.AFTHTAQYDEAISDYFR.R |  | 2.06 | 2.05 |
| 1011 | *leukotriene A-4 hydrolase isoform 1 [Homo sapiens]* | LTA4H | NP_000886 | 69868/5.80 | 367 | 35 | 3 | 1917.9599  1756.8562  1999.9989 | 1916.9803  1755.8693  1999.0123 | 25  136  59 | R.TLTGTAALTVQSQEDNLR.S  R.HFNALGGWGELQNSVK.T  K.DLSSHQLNEFLAQTLQR.A | -1.2 |  |  |
| 1026 | *mitochondrial heat shock 60kD protein 1 variant 1 [Homo sapiens]* | HSPD1 | ACE06961 | 60813/5.83 | 726 | 36 | 5 | 1344.7030  1601.7305  1919.0445  2365.3077  960.5025  1684.8884 | 1343.7085  1600.7443  1918.0636  2364.3264  959.5036  1683.8978 | 101  76  169  120  17  132 | R.TVIIEQSWGSPK.V  K.CEFQDAYVLLSEK.K  K.ISSIQSIVPALEIANAHR.K  R.KPLVIIAEDVDGEALSTLVLNR.L  R.VTDALNATR.A  R.AAVEEGIVLGGGCALLR.C |  |  | 6.23 |
| 1032 | *dihydropyrimidinase-related protein 3 isoform X1 [Homo sapiens]* | DPYSL3 | XP_011535876 | 61869/5.69 | 146 | 37 | 2 | 922.4518  2008.7630 | 921.5072  2007.9108 | 17  50 | K.IFNLYPR.K  K.NHQSAAEYNIFEGMELR.G | 1.8 |  |  |
| 1036 | *mitochondrial heat shock 60kD protein 1 variant 1 [Homo sapiens]* | HSPD1 | ACE06961 | 60813/5.83 | 319 | 22 | 5 | 1344.7104  1919.0630  2365.3117  833.3836  1684.9032 | 1343.7085  1918.0636  2364.3264  832.3828  1683.8978 | 65  47  78  26  71 | R.TVIIEQSWGSPK.V  K.ISSIQSIVPALEIANAHR.K  R.KPLVIIAEDVDGEALSTLVLNR.L  K.APGFGDNR.K  R.AAVEEGIVLGGGCALLR.C | -1.16 |  | 2.88 |
| 1057 | *heterogeneous nuclear ribonucleoprotein K isoform X4 [Homo sapiens]* | HNRNPK | XP_016870158 | 48708/5.69 | 277 | 19 | 5 | 1579.7288  1780.8273  1098.4769  1194.7203 | 1578.6984  1779.7911  1097.4448  1193.6921 | 46  72  49  52 | K.RPAEDMEEEQAFK.R  R.TDYNASVSVPDSSGPER.I  K.GSDFDCELR.L  R.NLPLPPPPPPR.G |  | 2.19 | 2.24 |
| 1080 | *UDP-glucose 6-dehydrogenase isoform 1 [Homo sapiens]* | UGDH | NP_003350 | 55674/6.73 | 614 | 55 | 7 | 1913.0345  1497.7701  882.4282  1370.7201  1074.6275  1244.5896  1758.8171 | 1912.0094  1496.7511  881.4065  1369.6838  1073.5981  1243.5656  1757.7831 | 64  71  20  56  37  77  82 | R.INAWNSPTLPIYEPGLK.E  K.NLFFSTNIDDAIK.E  K.YIEACAR.R  R.VLIGGDETPEGQR.A  K.LAANAFLAQR.I  K.ASVGFGGSCFQK.D  R.YWQQVIDMNDYQR.R |  | 3.69 | 3.72 |
| 1098 | *pyruvate kinase PKM isoform X2 [Homo sapiens]* | PKM | XP_005254500 | 65108/8.20 | 197 | 24 | 2 | 1197.6551  1019.5202 | 1196.6401  1018.5083 | 43  30 | R.LDIDSPPITAR.N  K.GDYPLEAVR.M |  | 4.03 | 3.56 |
| 1104 | *fibrinogen beta chain isoform 3 preproprotein [Homo sapiens]* | FGB | NP_001369688 | 51692/8.86 | 428 | 51 | 6 | 1950.9683  1587.6580  980.4330  1239.5013  1668.6864  1032.5507 | 1949.9959  1586.6705  979.4359  1238.5105  1667.7079  1031.5553 | 18  20  26  74  90  41 | R.EEAPSLRPAPPPISGGGYR.A  K.LESDVSAQMEYCR.T  R.QDGSVDFGR.K  K.EDGGGWWYNR.C  R.YYWGGQYTWDMAK.H  K.IRPFFPQQ.- |  |  | -1.77 |
| 1122 | *unnamed protein product [Homo sapiens] similar to: thioredoxin reductase [Homo sapiens]* | TXNRD1 | BAG35193 | 55253/6.07 | 253 | 35 | 3 | 1645.9045  1749.8823  1159.6480 | 1644.8909  1748.8634  1158.6509 | 45  70  17 | K.VMVLDFVTPTPLGTR.W  K.VVYENAYGQFIGPHR.I  R.FLIATGERPR.Y |  |  | 1.72 |
| 1150 | *histone-binding protein RBBP4 isoform a [Homo sapiens]* | RBBP4 | NP_005601 | 47911/4.74 | 307 | 27 | 4 | 1251.5570  934.4837  950.4693  973.5452  2873.3469 | 1250.5415  933.4742  949.4691  972.5393  2872.3515 | 44  30  13  35  102 | K.EAAFDDAVEER.V  K.LMIWDTR.S  K.LMIWDTR.S + Oxidation (M)  K.TVALWDLR.N  K.IGEEQSPEDAEDGPPELLFIHGGHTAK.I |  | 2.06 | 2.04 |
| 1172 | *PREDICTED: inosine-5'-monophosphate dehydrogenase 2 [Homo sapiens]* | IMPDH2 | NP_000875 | 56226/6.44 | 164 | 32 | 3 | 1430.7168  1780.0000  1156.6620 | 1429.6773  1778.9778  1155.6248 | 26  12  22 | R.HGFCGIPITDTGR.M  K.LPIVNEDDELVAIIAR.T  K.NLIDAGVDALR.V |  |  | 2.06 |
| 1176 | *T-complex protein 1 subunit beta isoform 2 [Homo sapiens]* | CCT2 | NP_001185771 | 53027/6.00 | 229 | 36 | 3 | 1291.7101  1130.5396  1330.6456 | 1290.7197  1129.5451  1329.6524 | 28  49  40 | K.IHPQTIIAGWR.E  K.HGINCFINR.Q  R.GATQQILDEAER.S | -1.19 |  |  |
| 1196 | *aldehyde dehydrogenase, mitochondrial isoform 1 precursor [Homo sapiens]* | ALDH2 | NP_000681 | 56859/6.63 | 235 | 25 | 3 | 1132.5804  1531.7440  1403.7589 | 1131.5825  1530.7355  1402.7530 | 27  85  53 | R.AAFQLGSPWR.R  K.TIPIDGDFFSYTR.H  K.EEIFGPVMQILK.F | -1.46 |  |  |
| 1206 | *tryptophan-tRNA ligase, cytoplasmic isoform X2 [Homo sapiens]* | WARS1 | XP_024305476 | 49163/6.03 | 556 | 32 | 7 | 863.4759  1144.6361  972.5461  1416.6707  2325.2369  1801.0596 | 862.4450  1143.6077  971.5189  1415.6391  2324.1953  1800.0257 | 36  58  72  80  119  62 | R.GIFFSHR.D  K.KPFYLYTGR.G  K.HVTFNQVK.G  K.GIFGFTDSDCIGK.I  K.ISFPAIQAAPSFSNSFPQIFR.D  K.ALIEVLQPLIAEHQAR.R | -1.22 |  |  |
| 1209 | *aldehyde dehydrogenase, mitochondrial isoform 1 precursor [Homo sapiens]* | ALDH2 | NP_000681 | 56859/6.63 | 752 | 43 | 7 | 1132.5923  829.4869  973.4603  1531.7490  3019.4899  1403.7577  902.4909  1599.7886 | 1131.5825  828.4705  972.4341  1530.7355  3018.5087  1402.7530  901.4869  1598.7828 | 71  11  70  103  168  61  36  62 | R.AAFQLGSPWR.R  R.LADLIER.D  R.YYAGWADK.Y  K.TIPIDGDFFSYTR.H  K.EAGFPPGVVNIVPGFGPTAGAAIASHEDVDK.V  K.EEIFGPVMQILK.F  K.TIEEVVGR.A  R.ELGEYGLQAYTEVK.T | -1.42 |  |  |
| 1294 | *elongation factor 1-gamma [Homo sapiens]* | EEF1G | NP_001395 | 50429/6.25 | 385 | 25 | 6 | 1707.8771  821.4660  975.5213  1609.7984  1241.6414  1461.6453  1684.7785 | 1706.8641  820.4443  974.5185  1608.7871  1240.6452  1460.6361  1683.7682 | 62  14  10  44  36  49  86 | R.VLSAPPHFHFGQTNR.T  R.TFLVGER.V  K.QVLEPSFR.Q  R.WFLTCINQPQFR.A  K.STFVLDEFKR.K  K.DGWSLWYSEYR.F  R.EYFSWEGAFQHVGK.A |  | 3.05 |  |
| 1340 | *beta-tubulin [Homo sapiens]* | TUBB | AAC28654 | 50095/4.78 | 360 | 23 | 9 | 1301.7297  1615.9516  1631.9368  1077.6227  1130.6912  1159.7202  1028.6119  1287.8086  1696.9409 | 1300.6299  1614.8287  1630.8236  1076.5250  1129.5880  1158.6219  1027.5121  1286.7169  1695.8257 | 46  28  32  25  77  14  26  8  7 | R.ISVYYNEATGGK.Y  R.AILVDLEPGTMDSVR.S  R.AILVDLEPGTMDSVR.S + Oxidation (M)  K.IREEYPDR.I  R.FPGQLNADLR.K  K.LAVNMVPFPR.L + Oxidation (M)  K.TAVCDIPPR.G  R.KLAVNMVPFPR.L + Oxidation (M)  K.NSSYFVEWIPNNVK.T |  | 2.13 |  |
| 1350 | *retinal dehydrogenase 1A1 [Homo sapiens]* | ALDH1A1 | NP_000680 | 55454/6.30 | 220 | 30 | 2 | 1189.6269  1544.7975  1645.8293  1700.7987 | 1188.6040  1543.7671  1644.8035  1699.7729 | 51  61  15  25 | R.QAFQIGSPWR.T  R.TIPIDGNFFTYTR.H  R.IFVEESIYDEFVR.R  R.ELGEYGFHEYTEVK.T |  |  | 1.74 |
| 1357 | *PREDICTED: elongation factor 1-gamma [Homo sapiens]* | EEF1G | NP_001395 | 50429/6.25 | 158 | 33 | 2 | 1707.8337  1241.6365  1609.8113 | 1706.8641  1240.6452  1608.7871 | 18  26  15 | R.VLSAPPHFHFGQTNR.T  K.STFVLDEFKR.K  R.WFLTCINQPQFR.A |  | 3.23 |  |
| 1378 | *sorting nexin-6 isoform b [Homo sapiens]* | SNX6 | NP_689419 | 46905/5.81 | 110 | 14 | 2 | 1362.6778  1192.6213 | 1361.6584  1191.6036 | 43  37 | K.TVAMHEVFLCR.V  R.TFLLEYHNR.V | -1.14 |  |  |
| 1388 | *alpha-1-antitrypsin isoform X1 [Homo sapiens]* | SERPINA1 | XP_016876859 | 46878/5.37 | 200 | 26 | 3 | 1779.8161  1891.8973  1803.9970 | 1778.7609  1890.8483  1802.9526 | 30  53  43 | K.TDTSHHDQDHPTFNK.I  K.DTEEEDFHVDQVTTVK.V  K.LQHLENELTHDIITK.F |  | -2.22 | -1.87 |
| 1391 | *haptoglobin, partial [Homo sapiens]* | HP | AAC27432 | 38722/6.14 | 280 | 28 | 3 | 920.4607  980.4979  1707.8287 | 919.4552  979.4876  1706.8120 | 53  45  95 | K.GSFPWQAK.M  R.VGYVSGWGR.N  K.YVMLPVADQDQCIR.H |  | -2.24 | -1.89 |
| 1394 | *glucose-6-phosphate 1-dehydrogenase isoform b [Homo sapiens]* | G6PD | NP_001346945 | 59675/6.39 | 917 | 59 | 9 | 1664.8441  1807.7608  1762.8352  1731.7465  1173.6116  1002.5275  1273.6033  2344.0468  1191.5447 | 1663.8570  1806.7809  1761.8692  1730.7617  1172.6302  1001.5447  1272.6139  2343.0841  1190.5608 | 105  96  35  37  57  53  86  125  70 | R.DGLLPENTFIVGYAR.S  R.NSYVAGQYDDAASYQR.L  R.LNSHMNALHLGSQANR.L  K.NIHESCMSQIGWNR.I  R.LSNHISSLFR.E  R.IFGPIWNR.D  R.GGYFDEFGIIR.D  K.KPGMFFNPEESELDLTYGNR.Y  R.VGFQYEGTYK.W |  | 4.25 |  |
| 1425 | *isocitrate dehydrogenase [NADP] cytoplasmic [Homo sapiens]* | IDH1 | NP_001269316 | 46915/6.53 | 497 | 43 | 8 | 1695.7782  976.5591  1437.9025  1009.4500  1170.5757  1154.5320  1341.6741  1509.6654 | 1694.7604  975.5502  1436.8867  1008.4413  1169.5604  1153.5226  1340.6684  1508.6493 | 23  35  17  51  74  59  18  71 | K.ISGGSVVEMQGDEMTR.I  R.NILGGTVFR.E  R.LVSGWVKPIIIGR.H  R.HAYGDQYR.A  K.DIFQEIYDK.Q  K.SEGGFIWACK.N  K.TVEAEAAHGTVTR.H  R.SDYLNTFEFMDK.L |  | 2.47 | 2.50 |
| 1429 | *alpha-enolase isoform 3 [Homo sapiens]* | ENO1 | NP_001340275 | 47696/6.57 | 245 | 22 | 3 | 1804.9366  1425.7160  806.4331 | 1803.9366  1424.7187  805.4446 | 99  57  19 | R.AAVPSGASTGIYEALELR.D  R.YISPDQLADLYK.S  K.YNQLLR.I |  | 3.16 | 3.11 |
| 1430 | *alpha-enolase isoform 3 [Homo sapiens]* | ENO1 | NP_001340275 | 47696/6.57 | 614 | 38 | 4 | 1406.7261  1804.9424  1425.7203  2033.0286  806.4331 | 1405.7089  1803.9366  1424.7187  2032.0477  805.4446 | 80  155  104  114  40 | R.GNPTVEVDLFTSK.G  R.AAVPSGASTGIYEALELR.D  R.YISPDQLADLYK.S  K.FTASAGIQVVGDDLTVTNPK.R  K.YNQLLR.I |  | 4.96 |  |
| 1452 | *adenosylhomocysteinase isoform X2 [Homo sapiens]* | AHCY | XP_016883198 | 48255/5.92 | 340 | 28 | 6 | 1128.6146  1559.7645  1575.7657  1056.6229  1259.5898  1102.5656 | 1127.6087  1558.7483  1574.7432  1055.6127  1258.5764  1101.5454 | 68  33  26  47  17  39 | K.VADIGLAAWGR.K  K.ALDIAENEMPGLMR.M  K.ALDIAENEMPGLMR.M + Oxidation (M)  K.YPQLLPGIR.G  K.SKFDNLYGCR.E  K.WLNENAVEK.V |  |  | 1.84 |
| 1467 | *unknown [Homo sapiens] similar to:*  *beta actin variant, partial [Homo sapiens]* | ACTB | AAP22343 | 42052/5.29 | 356 | 43 | 5 | 1171.6353  1515.8157  3183.6997  1132.5962  2343.2376 | 1170.5638  1514.7419  3182.6071  1131.5197  2342.1576 | 69  75  28  59  25 | R.HQGVMVGMGQK.D  K.IWHHTFYNELR.V  R.TTGIVMDSGDGVTHTVPIYEGYALPHAILR.L  R.GYSFTTTAER.E  R.KDLYANTVLSGGTTMYPGIADR.M |  | -1.52 | -1.35 |
| 1532 | *citrate synthase, mitochondrial precursor [Homo sapiens]* | CS | NP_004068 | 51908/8.45 | 333 | 33 | 3 | 1762.8993  1338.6588  1127.6632 | 1761.8785  1337.6364  1126.6499 | 92  90  32 | K.GLVYETSVLDPDEGIR.F  R.DYIWNTLNSGR.V  R.ALGFPLERPK.S | -1.25 |  | 2.22 |
| 1542 | *elongation factor 1-alpha 1 [Homo sapiens]* | EEF1A1 | NP_001393 | 50451/9.10 | 283 | 25 | 4 | 1404.7308  3149.6545  1025.6225  2515.3733 | 1403.7197  3148.6550  1024.6030  2514.3768 | 75  35  52  60 | K.YYVTIIDAPGHR.D  R.KDGNASGTTLLEALDCILPPTRPTDKPLR.L  K.IGGIGTVPVGR.V  R.VETGVLKPGMVVTFAPVNVTTEVK.S |  | 3.09 | 3.09 |
| 1596 | *leukocyte elastase inhibitor isoform X1 [Homo sapiens]* | SERPINB1 | XP_011512636 | 42829/5.90 | 332 | 28 | 5 | 1602.7199  1686.8173  2052.8525  1218.5621  1785.8457 | 1601.7587  1685.8665  2051.9185  1217.5968  1784.8944 | 45  49  134  54  8 | K.TFHFNTVEEVHSR.F  K.TYNFLPEFLVSTQK.T  K.TYGADLASVDFQHASEDAR.K  K.FAYGYIEDLK.C  R.FKLEESYTLNSDLAR.L |  |  | 2.70 |
| 1611 | *40S ribosomal protein SA isoform 1 [Homo sapiens]* | RPSA | NP_002286 | 32947/4.79 | 674 | 48 | 6 | 1740.9223  1203.6319  1698.8397  912.5345  2996.4136  1881.8327 | 1739.9417  1202.6408  1697.8526  911.5440  2995.4709  1880.8556 | 105  96  123  19  166  98 | R.AIVAIENPADVSVISSR.N  K.FAAATGATPIAGR.F  R.FTPGTFTNQIQAAFR.E  R.LLVVTDPR.A  R.ADHQPLTEASYVNLPTIALCNTDSPLR.Y  R.EHPWEVMPDLYFYR.D |  | 2.03 | 1.87 |
| 1614 | *interleukin enhancer-binding factor 2 isoform 2 [Homo sapiens]* | ILF2 | NP_001254738 | 39057/4.90 | 178 | 31 | 3 | 1732.0569  2582.6137  1236.8883 | 1730.9090  2581.3752  1235.7601 | 52  25  42 | R.VKPAPDETSFSEALLK.R  K.INNVIDNLIVAPGTFEVQIEEVR.Q  K.ILITTVPPNLR.K |  | -1.36 |  |
| 1662 | *unnamed protein product [Homo sapiens] similar to: 26S proteasome non-ATPase regulatory subunit 7 [Homo sapiens]* | PSMD7 | BAG37774 | 37046/6.29 | 193 | 39 | 2 | 1157.5977  2573.1923 | 1156.6029  2572.2181 | 59  64 | R.IVGWYHTGPK.L  K.DLGLPTEAYISVEEVHDDGTPTSK.T |  | 1.93 | 1.85 |
| 1714 | *elongation factor 1-delta isoform X2 [Homo sapiens]* | EEF1D | XP_024302856 | 71834/6.02 | 156 | 26 | 2 | 2185.0846  1358.7636  1733.8808 | 2184.0407  1357.7201  1732.8420 | 13  50  41 | K.SLAGSSGPGASSGTSGDHGELVVR.I  R.IASLEVENQSLR.G  K.FEEHVQSVDIAAFNK.I |  |  | -1.44 |
| 1716 | *tubulin beta chain isoform d [Homo sapiens]* | TUBB | NP_001280143 | 45246/4.88 | 271 | 33 | 5 | 1130.5758  1143.6030  1039.5627  1696.7980  1229.5815 | 1129.5880  1142.6270  1038.5862  1695.8257  1228.5910 | 62  28  35  42  33 | R.FPGQLNADLR.K  K.LAVNMVPFPR.L  R.YLTVAAVFR.G  K.NSSYFVEWIPNNVK.T  R.ISEQFTAMFR.R | 1.84 |  |  |
| 1720 | *inorganic pyrophosphatase [Homo sapiens]* | PPA1 | NP_066952 | 33095/5.54 | 261 | 36 | 3 | 1053.5456  1863.9372  1327.7115  1694.9062 | 1052.5291  1862.9203  1326.6755  1693.8828 | 15  63  40  65 | R.AAPFSLEYR.V  K.GQYISPFHDIPIYADK.D  K.DVFHMVVEVPR.W  R.LKPGYLEATVDWFR.R |  | -2.03 | -1.42 |
| 1828 | *carbonic anhydrase 1 isoform a [Homo sapiens]* | CA1 | NP_001158302 | 28909/6.59 | 140 | 40 | 2 | 985.4426  1580.8063 | 984.4301  1579.7842 | 42  48 | K.GGPFSDSYR.L  K.ESISVSSEQLAQFR.S | 1.73 | 2.09 |  |
| 1845 | *hepatoma-derived growth factor isoform a [Homo sapiens]* | HDGF | NP_004485 | 26886/4.70 | 203 | 32 | 3 | 983.4811  1127.5235  1819.8857 | 982.4773  1126.5182  1818.8788 | 32  47  58 | K.GYPHWPAR.I  K.DLFPYEESK.E  K.GFSEGLWEIENNPTVK.A |  | 2.00 |  |
| 1856 | *aldose reductase [Homo sapiens]* | AKR1B1 | AAA51712 | 36231/6.51 | 217 | 35 | 3 | 892.4767  1120.6193  1083.4440 | 891.4814  1119.6288  1082.4345 | 30  21  58 | K.VAIDVGYR.H  K.REELFIVSK.L  K.DYPFHEEF.- |  | 1.76 |  |
| 1860 | *glyceraldehyde-3-phosphate dehydrogenase isoform 4 [Homo sapiens]* | GAPDH | NP_001344872 | 34075/8.29 | 528 | 34 | 4 | 2369.2230  2595.3667  1530.8094  1763.8274 | 2368.2031  2594.3527  1529.7872  1762.7951 | 15  193  139  117 | K.RVIISAPSADAPMFVMGVNHEK.Y  K.VIHDNFGIVEGLMTTVHAITATQK.T  R.VPTANVSVVDLTCR.L  K.LISWYDNEFGYSNR.V |  | 2.17 | 2.14 |
| 1880 | *annexin A2 isoform X1 [Homo sapiens]* | ANXA2 | XP_016877580 | 38808/7.57 | 1140 | 66 | 10 | 1844.8809  1542.8455  1650.9609  1244.6220  1811.8539  1225.5705  2064.9748  1908.8682  1460.6663  1421.6755 | 1843.8952  1541.8413  1649.9716  1243.6156  1810.8585  1224.5623  2063.9760  1907.8748  1459.6653  1420.6874 | 116  107  99  82  43  55  166  156  77  76 | K.LSLEGDHSTPPSAYGSVK.A  K.GVDEVTIVNILTNR.S  K.SALSGHLETVILGLLK.T  R.TNQELQEINR.V  K.TDLEKDIISDTSGDFR.K  K.DIISDTSGDFR.K  R.RAEDGSVIDYELIDQDAR.D  R.AEDGSVIDYELIDQDAR.D  K.SYSPYDMLESIR.K  K.SLYYYIQQDTK.G |  | -1.63 |  |
| 1958 | *annexin A3 [Homo sapiens]* | ANXA3 | NP_005130 | 36524/5.63 | 646 | 42 | 8 | 1781.8508  929.5283  1673.9003  1350.6894  1585.7305  1882.9320  943.4624  1018.5516  1222.6417 | 1780.8156  928.4290  1672.8632  1349.6503  1584.6904  1881.8931  942.4447  1017.5243  1221.5990 | 111  16  71  69  54  52  48  42  36 | R.DYPDFSPSVDAEAIQK.A  K.EYQAAYGK.E  K.GAGTNEDALIEILTTR.T  K.DISQAYYTVYK.K  K.SLGDDISSETSGDFR.K  R.WGTDEDKFTEILCLR.S  K.LTFDEYR.N  R.NTPAFLAER.L  K.GIGTDEFTLNR.I |  | -2.25 | -1.51 |
| 1961 | *carbonic anhydrase 1 isoform a [Homo sapiens]* | CA1 | NP_001158302 | 28909/6.59 | 485 | 50 | 4 | 1928.9415  2255.9776  1612.7365  1580.7462 | 1928.0003  2255.0356  1611.7794  1579.7842 | 133  56  102  126 | K.HDTSLKPISVSYNPATAK.E  K.EIINVGHSFHVNFEDNDNR.S  K.YSAELHVAHWNSAK.Y  K.ESISVSSEQLAQFR.S | -1.19 |  |  |
| 1975 | *elongation factor 1-beta [Homo sapiens]* | EEF1B2 | NP_001032752 | 24919/4.50 | 294 | 38 | 3 | 1603.8464  945.5716  860.4401 | 1602.8253  944.5695  859.4341 | 132  58  32 | K.SPAGLQVLNDYLADK.S  K.LVPVGYGIK.K  R.WYNHIK.S |  | 2.46 | 2.23 |
| 1995 | *annexin A4 isoform X1 [Homo sapiens]* | ANXA4 | XP_024308603 | 36290/5.84 | 594 | 41 | 7 | 1597.7357  1692.8838  1661.8228  1371.6781  1118.5059  1134.5288  1414.7245  1570.8155 | 1596.7202  1691.8730  1660.8090  1370.6579  1117.4863  1133.4812  1413.7041  1569.8052 | 50  93  69  75  44  12  80  68 | K.AASGFNAMEDAQTLR.K + Oxidation (M)  K.GLGTDEDAIISVLAYR.N  K.GAGTDEGCLIEILASR.T  R.ISQTYQQQYGR.S  R.SDTSFMFQR.V  R.SDTSFMFQR.V + Oxidation (M)  R.NHLLHVFDEYK.R  R.NHLLHVFDEYKR.I |  | 1.36 |  |
| 2049 | *carbonic anhydrase 1 isoform a [Homo sapiens]* | CA1 | NP_001158302 | 28909/6.59 | 573 | 54 | 6 | 945.4243  2256.0156  985.4239  1612.7590  1580.7770  2775.3351 | 944.4352  2255.0356  984.4301  1611.7794  1579.7842  2774.3770 | 54  50  69  117  130  53 | K.NGPEQWSK.L  K.EIINVGHSFHVNFEDNDNR.S  K.GGPFSDSYR.L  K.YSAELHVAHWNSAK.Y  K.ESISVSSEQLAQFR.S  R.SLLSNVEGDNAVPMQHNNRPTQPLK.G + Oxidation (M) |  | -5.23 | -4.09 |
| 2156 | *unnamed protein product [Homo sapiens] similar to:* *serum albumin [Homo sapiens]* | ALB | BAG37325 | 71343/5.92 | 340 | 20 | 5 | 2045.0921  1657.7616  960.5659  1639.9438  1342.6568  1358.6370 | 2044.0881  1656.7453  959.5552  1638.9305  1341.6275  1357.6224 | 44  67  25  20  88  49 | K.VFDEFKPLVEEPQNLIK.Q  K.QNCELFEQLGEYK.F  K.FQNALLVR.Y  K.KVPQVSTPTLVEVSR.N  K.AVMDDFAAFVEK.C  K.AVMDDFAAFVEK.C + Oxidation (M) | -1.25 | -3.92 | -3.29 |
| 2314 | *proteasome subunit alpha type-2 [Homo sapiens]* | PSMA1 | NP_002778 | 25996/6.92 | 144 | 36 | 3 | 895.4639  1564.7583  1580.7512 | 894.4447  1563.7504  1579.7453 | 37  47  14 | K.SILYDER.S  K.HIGLVYSGMGPDYR.V  K.HIGLVYSGMGPDYR.V + Oxidation (M) | -1.4 |  |  |
| 2341 | *apolipoprotein A-I isoform 1 preproprotein [Homo sapiens]* | APOA1 | NP_001304947 | 30759/5.56 | 665 | 54 | 6 | 1612.8073  1932.9505  1252.6413  1283.6019  1031.5392  1386.7373 | 1611.7781  1931.9265  1251.6136  1282.5652  1030.5117  1385.7078 | 142  119  59  58  35  105 | K.LLDNWDSVTSTFSK.L  R.EQLGPVTQEFWDNLEK.E  K.VQPYLDDFQK.K  K.WQEEMELYR.Q  K.LSPLGEEMR.D  K.VSFLSALEEYTK.K |  | -2.06 |  |
| 2483 | *Transgelin [Homo sapiens]* | TAGLN | NP_001001522 | 22653/8.87 | 254 | 57 | 2 | 2109.1326  990.6005 | 2108.0837  989.5698 | 76  42 | R.LVEWIIVQCGPDVGRPDR.G  R.LGFQVWLK.N |  |  | -1.69 |
| 2486 | *peroxiredoxin-2 [Homo sapiens]* | PRDX2 | NP_005800 | 22049/5.66 | 370 | 42 | 4 | 2699.3600  1863.0783  924.4595  1211.7359 | 2698.3537  1862.0625  923.4348  1210.6670 | 64  109  18  68 | K.LGCEVLGVSVDSQFTHLAWINTPR.K  R.KEGGLGPLNIPLLADVTR.R  K.TDEGIAYR.G  R.QITVNDLPVGR.S |  | -1.39 |  |
| 2544 | *marginal zone B- and B1-cell-specific protein precursor [Homo sapiens]* | MZB1 | NP_057543 | 21023/5.37 | 383 | 46 | 4 | 1551.7976  1037.4836  2651.1952  1817.8630 | 1550.7828  1036.4726  2650.1870  1816.8448 | 92  47  93  82 | R.ELSELVYTDVLDR.S  R.NWQDYGVR.E  R.TCLHYLGEFGEDQIYEAHQQGR.G  R.GALEALLCGGPQGACSEK.V |  | 4.86 | 3.27 |
| 2576 | *nucleoside diphosphate kinase A isoform a [Homo sapiens]* | NME1 | NP_937818 | 19869/5.42 | 278 | 37 | 4 | 1344.7664  1149.6406  1785.9043  1051.4994 | 1343.7561  1148.6342  1784.9091  1050.4917 | 70  62  42  62 | R.TFIAIKPDGVQR.G  K.DRPFFAGLVK.Y  R.VMLGETNPADSKPGTIR.G  R.GDFCIQVGR.N |  | 2.66 | 2.63 |
| 2633 | *peroxiredoxin-5, mitochondrial isoform S [Homo sapiens]* | PRDX5 | NP_001345445 | 17191/6.73 | 317 | 67 | 4 | 1827.8524  1593.7572  1539.7683  1906.9108 | 1826.9050  1592.8021  1538.8093  1905.9684 | 87  49  87  30 | K.VGDAIPAVEVFEGEPGNK.V  K.GVLFGVPGAFTPGCSK.T  K.THLPGFVEQAEALK.A  K.ETDLLLDDSLVSIFGNR.R |  | -1.66 | -1.81 |
| 2704 | *peptidyl-prolyl cis-trans isomerase A isoform 1 [Homo sapiens]* | PPIA | NP_066953 | 18229/7.68 | 259 | 50 | 3 | 1945.9864  1598.7379  1154.5616 | 1944.9946  1597.7381  1153.5655 | 60  77  47 | M.VNPTVFFDIAVDGEPLGR.V  R.IIPGFMCQGGDFTR.H  K.FEDENFILK.H |  | -1.49 | -1.50 |
| 2808 | *profilin-1 isoform 2 [Homo sapiens]* | PFN1 | NP_005013 | 15216/8.44 | 238 | 46 | 5 | 1625.7543  1641.7546  1379.7159  1166.5048  1182.5167 | 1624.7403  1640.7352  1378.7093  1165.5008  1181.4957 | 111  36  27  42  53 | R.DSLLQDGEFSMDLR.T  R.DSLLQDGEFSMDLR.T + Oxidation (M)  K.STGGAPTFNVTVTK.T  K.CYEMASHLR.R  K.CYEMASHLR.R + Oxidation (M) |  | -1.46 |  |
| 2813 | *myosin light chain [Homo sapiens]* | MYL3 | AAA59853 | 17091/4.51 | 360 | 41 | 5 | 1025.5065  1341.6320  1903.9743  1786.8082  1544.6857 | 1024.4978  1340.6217  1902.9914  1785.8169  1543.6791 | 65  48  29  55  99 | K.EAFQLFDR.T  K.ILYSQCGDVMR.A  K.VLDFEHFLPMLQTVAK.N + Oxidation (M)  K.NKDQGTYEDYVEGLR.V  K.DQGTYEDYVEGLR.V | 1.73 |  |  |
| 2871 | *LGALS1 [Homo sapiens]* | LGALS1 | CAG30397 | 15048/5.34 | 548 | 70 | 5 | 1486.6676  1647.7246  1076.4665  968.4581  1784.8274 | 1485.6783  1646.7471  1075.4683  967.4651  1783.8450 | 93  106  78  63  130 | K.DSNNLCLHFNPR.F  R.FNAHGDANTIVCNSK.D  K.DGGAWGTEQR.E  K.LPDGYEFK.F  R.LNLEAINYMAADGDFK.I | 1.38 |  |  |
| 2907 | *hemoglobin subunit delta [Homo sapiens]* | HBD | NP_000510 | 16159/7.85 | 366 | 62 | 4 | 1256.6994  2060.9282  1669.9219  1126.5884 | 1255.6521  2059.9197  1668.8835  1125.5567 | 21  102  109  52 | K.VNVDAVGGEALGR.L  R.FFESFGDLSSPDAVMGNPK.V + Oxidation (M)  K.VLGAFSDGLAHLDNLK.G  K.LHVDPENFR.L |  |  | -2.36 |
| 2920 | *hemoglobin subunit beta [Homo sapiens]* | HBB | QBH68943 | 16102/6.75 | 512 | 77 | 4 | 2074.8917  1669.8576  1126.5412  1378.6752 | 2073.9354  1668.8835  1125.5567  1377.6929 | 130  103  65  82 | R.FFESFGDLSTPDAVMGNPK.V + Oxidation (M)  K.VLGAFSDGLAHLDNLK.G  K.LHVDPENFR.L  K.EFTPPVQAAYQK.V |  | -10.51 | -8.79 |
| 2921 | *hemoglobin alpha 1 globin chain, partial [Homo sapiens]* | HBA1 | ADV59921 | 10776/8.09 | 419 | 69 | 3 | 1529.7662  1071.5710  1833.9202 | 1528.7270  1070.5471  1832.8846 | 158  50  146 | K.VGAHAGEYGAEALER.M  R.MFLSFPTTK.T  K.TYFPHFDLSHGSAQVK.G |  |  | -2.40 |
| 2923 | *hemoglobin subunit delta [Homo sapiens]* | HBD | NP_000510 | 16159/7.85 | 449 | 77 | 3 | 1669.8736  2629.2087  1126.5439  1441.6810 | 1668.8835  2628.2391  1125.5567  1440.6707 | 130  89  55  63 | K.VLGAFSDGLAHLDNLK.G  K.GTFSQLSELHCDKLHVDPENFR.L  K.LHVDPENFR.L  K.EFTPQMQAAYQK.V |  | -2.74 | -2.14 |
| 2927 | *hemoglobin subunit beta [Homo sapiens]* | HBB | NP_000509 | 16102/6.75 | 464 | 53 | 4 | 2074.9509  1669.9080  1126.5781  1378.7090 | 2073.9354  1668.8835  1125.5567  1377.6929 | 90  139  71  77 | R.FFESFGDLSTPDAVMGNPK.V + Oxidation (M)  K.VLGAFSDGLAHLDNLK.G  K.LHVDPENFR.L  K.EFTPPVQAAYQK.V |  | -6.71 | -4.67 |
| 2928 | *hemoglobin subunit beta [Homo sapiens]* | HBB | QBH68942 | 16102/6.75 | 401 | 54 | 4 | 2074.9557  1669.9013  1126.5948  1378.7240 | 2073.9354  1668.8835  1125.5567  1377.6929 | 103  79  68  61 | R.FFESFGDLSTPDAVMGNPK.V + Oxidation (M)  K.VLGAFSDGLAHLDNLK.G  K.LHVDPENFR.L  K.EFTPPVQAAYQK.V |  | -6.46 |  |
| 2929 | *mutant hemoglobin subunit alpha 2 [Homo sapiens]* | HBA1 | AXY54999 | 15177/7.96 | 399 | 68 | 3 | 1529.7162  1071.5382  1833.8580 | 1528.7270  1070.5471  1832.8846 | 147  63  137 | K.VGAHAGEYGAEALER.M  R.MFLSFPTTK.T  K.TYFPHFDLSHGSAQVK.G |  | -2.08 | -1.50 |
| 2936 | *hemoglobin subunit beta [Homo sapiens]* | HBB | NP_000509 | 16102/6.75 | 679 | 88 | 5 | 2074.9646  1669.9407  2586.2580  1126.6015  1378.7407 | 2073.9354  1668.8835  2585.2333  1125.5567  1377.6929 | 134  129  81  82  82 | R.FFESFGDLSTPDAVMGNPK.V + Oxidation (M)  K.VLGAFSDGLAHLDNLK.G  K.GTFATLSELHCDKLHVDPENFR.L  K.LHVDPENFR.L  K.EFTPPVQAAYQK.V |  | -3.75 | -2.63 |
| 3038 | *hemoglobin alpha 1 globin chain, partial [Homo sapiens]* | HBA1 | ADV59921 | 10776/8.09 | 397 | 69 | 4 | 1529.7706  1071.5779  1087.6212  1833.9295 | 1528.7270  1070.5471  1086.5420  1832.8846 | 154  51  41  133 | K.VGAHAGEYGAEALER.M  R.MFLSFPTTK.T  R.MFLSFPTTK.T + Oxidation (M)  K.TYFPHFDLSHGSAQVK.G |  | -8.54 | -5.68 |
| 3045 | *protein S100-A4 [Homo sapiens]* | S100A4 | NP_062427 | 11949/5.85 | 135 | 27 | 2 | 1247.6236  890.4714 | 1246.6380  889.4909 | 59  55 | K.ALDVMVSTFHK.Y  R.ELPSFLGK.R |  | -1.79 | -1.48 |
| 3050 | *beta globin, partial [Homo sapiens]* | HBB | ULD54884 | 11554/5.90 | 588 | 85 | 4 | 2074.9263  1669.8960  2586.2189  1126.5700 | 2073.9354  1668.8835  2585.2333  1125.5567 | 157  129  78  92 | R.FFESFGDLSTPDAVMGNPK.V + Oxidation (M)  K.VLGAFSDGLAHLDNLK.G  K.GTFATLSELHCDKLHVDPENFR.-  K.LHVDPENFR.- |  | -11.78 |  |
| 3150 | *hemoglobin subunit beta [Homo sapiens]* | HBB | QBH68942 | 16102/6.75 | 594 | 88 | 5 | 932.4919  1669.8778  2586.2286  1126.5592  1378.6940 | 931.5127  1668.8835  2585.2333  1125.5567  1377.6929 | 22  131  158  65  79 | K.SAVTALWGK.V  K.VLGAFSDGLAHLDNLK.G  K.GTFATLSELHCDKLHVDPENFR.L  K.LHVDPENFR.L  K.EFTPPVQAAYQK.V |  | -2.41 | -1.73 |
| 3151 | *hemoglobin subunit alpha [Homo sapiens]* | HBA2 | NP_000549 | 15305/8.72 | 684 | 80 | 5 | 1529.7657  1071.5719  1833.9250  2996.5277  1087.6315 | 1528.7270  1070.5471  1832.8846  2995.4821  1086.6186 | 162  48  133  221  28 | K.VGAHAGEYGAEALER.M  R.MFLSFPTTK.T  K.TYFPHFDLSHGSAQVK.G  K.VADALTNAVAHVDDMPNALSALSDLHAHK.L  K.LRVDPVNFK.L |  | -3.12 | -2.36 |
| 3152 | *mutant hemoglobin subunit alpha 2 [Homo sapiens]* | HBA1 | AXY55003 | 15348/9.07 | 433 | 61 | 4 | 1529.7433  1071.5595  1087.5854  1833.8726 | 1528.7270  1070.5471  1086.5420  1832.8846 | 153  51  26  133 | K.VGAHAGEYGAEALER.M  R.MFLSFPTTK.T  R.MFLSFPTTK.T + Oxidation (M)  K.TYFPHFDLSHGSAQVK.G |  | -2.27 | -1.34 |
| 3153 | *calreticulin precursor [Homo sapiens]* | CALR | NP_004334 | 48283/4.29 | 715 | 42 | 6 | 1410.6519  975.4975  1607.7775  1147.7010  1019.5929  1800.8799  2760.3128 | 1409.6212  974.4781  1606.7667  1146.6550  1018.5600  1799.8326  2759.2562 | 116  13  107  72  50  97  109 | K.EQFLDGDGWTSR.W  K.GLQTSQDAR.F  R.FYALSASFEPFSNK.G  K.KVHVIFNYK.G  K.VHVIFNYK.G  K.IKDPDASKPEDWDER.A  K.IDDPTDSKPEDWDKPEHIPDPDAK.K |  | 7.09 |  |
| 3154 | *14-3-3 protein zeta/delta isoform X1 [Homo sapiens]* | YWHAZ | XP_024303034 | 27899/4.73 | 727 | 53 | 4 | 1548.7123  1304.6679  2040.9664  1252.6311  1124.5417  2131.9605  1189.6526  1205.6512 | 1547.7063  1303.6772  2039.9800  1251.6394  1123.5444  2130.9844  1188.6536  1204.6485 | 132  52  149  45  39  126  24  37 | K.SVTEQGAELSNEER.N  K.FLIPNASQAESK.V  K.GIVDQSQQAYQEAFEISK.K  K.KEMQPTHPIR.L + Oxidation (M)  K.EMQPTHPIR.L + Oxidation (M)  K.TAFDEAIAELDTLSEESYK.D  K.DSTLIMQLLR.D  K.DSTLIMQLLR.D + Oxidation (M) |  | 2.38 |  |
| 3155 | *keratin, type I cytoskeletal 19 [Homo sapiens]* | KRT19 | NP_002267 | 44079/5.04 | 697 | 62 | 8 | 1554.8373  1104.6229  1008.5568  1674.7941  993.5551  1082.5427  1485.7782  1389.7868  1122.6505 | 1553.7434  1103.5393  1007.5188  1673.7685  992.4927  1081.4829  1484.7042  1388.6783  1121.5717 | 84  15  23  108  30  55  34  45  33 | R.QSSATSSFGGLGGGSVR.F  K.LTMQNLNDR.L  K.IRDWYQK.Q  R.DYSHYYTTIQDLR.D  K.FETEQALR.M  K.DAEAWFTSR.T  R.EVAGHTEQLQMSR.S  K.AALEDTLAETEAR.F  R.LEQEIATYR.S |  |  | -1.56 |
