# Supplementary material for: Application of two-dimensional difference gel electrophoresis to identify protein changes between center, margin, and adjacent non-tumor tissues obtained from non-small-cell lung cancer with adenocarcinoma or squamous cell carcinoma subtype
Source: PLoS One. 2022 May 5;17(5):e0268073. doi: 10.1371/journal.pone.0268073 (PMC9071164; doi:10.1371/journal.pone.0268073)
Supplement: S2 Table — (DOCX) [file pone.0268073.s002.docx]

| **S2 Table.** Proteins found to be present in different abundances in the lung cancer of center and margin of SCC tumor in relation to control | | | | | | | | | | | | | | |
| --- | --- | --- | --- | --- | --- | --- | --- | --- | --- | --- | --- | --- | --- | --- |
| **Spot no in Fig. 1c-d** | **Proein name** | **Gene name** | **Accession**  **number** | **Calculated**  **MW/pI** | **Protein score** | **Sequence coverage**  **%** | **No of unique peptides** | **Precursor mass** | | **Peptide score** | **Peptide sequence** | **Centrum vs Margin** | **Centrum vs Control** | **Margin vs Control** |
|  |  |  |  |  |  |  |  | **Observed** | **Theoretical** |  |  |  |  |  |
| 394 | elongation factor 2 [*Homo sapiens*] | EEF2 | NP_001952 | 96246/6.41 | 244 | 19 | 5 | 1091.6322  1274.7644  1543.8322  1138.5751  760.3913  1378.7644 | 1090.5771  1273.7030  1542.7678  1137.5091  759.3704  1377.7075 | 55  19  13  42  37  8 | M.VNFTVDQIR.A  K.EDLYLKPIQR.T  K.ARPFPDGLAEDIDK.G  K.YEWDVAEAR.K  R.FYAFGR.V  R.CLYASVLTAQPR.L |  | 5.91 | 4.75 |
| 403 | elongation factor 2 [*Homo sapiens*] | EEF2 | NP_001952 | 96246/6.41 | 259 | 23 | 3 | 1091.5761  890.4902  2143.0511  1138.5095  969.5199  1378.6998 | 1090.5771  889.5022  2142.0705  1137.5091  968.5403  1377.7075 | 50  11  19  51  15  10 | M.VNFTVDQIR.A  K.FSVSPVVR.V  K.ARPFPDGLAEDIDKGEVSAR.Q  K.YEWDVAEAR.K  R.GGGQIIPTAR.R  R.CLYASVLTAQPR.L |  | 14.13 | 9.35 |
| 439 | unnamed protein product [Homo sapiens] similar to: ezrin [*Homo sapiens*] | EZR | BAG35489 | 69472/5.94 | 338 | 21 | 4 | 1669.7897  1104.5738  1310.6866  1182.5952 | 1668.7936  1103.5764  1309.6819  1181.5869 | 52  67  57  97 | R.EVWYFGLHYVDNK.G  K.IGFPWSEIR.N  K.KAPDFVFYAPR.L  K.APDFVFYAPR.L |  |  | 7.34 |
| 444 | tumor rejection antigen (gp96) 1 variant, partial [*Homo sapiens*] | TRA1 | CAI64497 | 92567/4.77 | 320 | 19 | 5 | 1529.7943  1187.7002  1627.7312  1485.7633  2260.1045 | 1528.7668  1186.6710  1626.7058  1484.7471  2259.0556 | 22  63  44  55  42 | K.NLLHVTDTGVGMTR.E + Oxidation (M)  K.SILFVPTSAPR.G  R.VFITDDFHDMMPK.Y + 2 Oxidation (M)  K.GVVDSDDLPLNVSR.E  R.FQSSHHPTDITSLDQYVER.M |  | 11.84 | 10.64 |
| 516 | keratin, type I cytoskeletal 10 isoform 1 [*Homo sapiens*] | KRT10 | NP_000412.4 | 59020/5.13 | 286 | 31 | 5 | 1707.7945  2367.2646  807.4131  1365.6562  1390.7017  1434.7837 | 1706.7649  2366.2553  806.3923  1364.6320  1389.6736  1433.7626 | 12  25  19  48  30  19 | K.GSLGGGFSSGGFSGGSFSR.G  K.NQILNLTTDNANILLQIDNAR.L  R.LAADDFR.L  R.SQYEQLAEQNR.K  K.QSLEASLAETEGR.Y  K.IRLENEIQTYR.S |  | 3.62 | 3.12 |
| 539 | aconitate hydratase, mitochondrial isoform X2 [*Homo sapiens*] | ACO2 | XP_016884301.1 | 82405 /6.61 | 121 | 7 | 2 | 985.5204  935.5057  1667.7640 | 984.5029  934.4872  1666.7587 | 35  11  41 | K.EGWPLDIR.V  R.DGYAQILR.D  R.WVVIGDENYGEGSSR.E |  | 2.28 | 2.13 |
| 547 | aconitate hydratase, mitochondrial isoform X2 [*Homo sapiens*] | ACO2 | XP_016884301 | 82405/6.61 | 250 | 18 | 4 | 985.4980  1463.7271  935.4754  1667.7498 | 984.5029  1462.7416  934.4872  1666.7587 | 46  28  31  75 | K.EGWPLDIR.V  K.SQFTITPGSEQIR.A  R.DGYAQILR.D  R.WVVIGDENYGEGSSR.E |  | 4.70 | 4.91 |
| 558 | heat shock protein HSP 90-alpha isoform X1 [*Homo sapiens*] | HSP90AA1 | XP_011535020.1 | 98583/5.07 | 163 | 18 | 3 | 815.5128  1264.6568  1108.5468 | 814.5065  1263.6360  1107.5349 | 32  35  33 | R.ALLFVPR.R  R.RAPFDLFENR.K  R.APFDLFENR.K |  | 20.95 | 13.69 |
| 560 | heat shock protein HSP 90-beta isoform c [*Homo sapiens*] | HSP90AB1 | NP_001258901.1 | 82611/4.98 | 325 | 26 | 3 | 1194.7166  1348.7487  1236.7165  1783.0593 | 1193.6404  1347.6572  1235.6299  1781.9424 | 50  83  25  78 | K.IDIIPNPQER.T  K.HFSVEGQLEFR.A  R.RAPFDLFENK.K  K.HLEINPDHPIVETLR.Q |  | 15.09 | 16.04 |
| 562 | serotransferrin isoform 2 [*Homo sapiens*] | TF | NP_001341632.2 | 74289/6.63 | 418 | 34 | 10 | 1881.9598  2549.3842  1689.9195  1478.8059  1195.6151  1249.6866  1577.7454  1283.6368  1354.6997  1586.8460 | 1880.8687  2548.2856  1688.8417  1477.7275  1194.5352  1248.5986  1576.6504  1282.5618  1353.6235  1585.7671 | 29  25  39  20  25  24  45  36  28  23 | K.ADRDQYELLCLDNTR.K  R.KPVDEYKDCHLAQVPSHTVVAR.S  K.DCHLAQVPSHTVVAR.S  K.MYLGYEYVTAIR.N  K.WCALSHHER.L  K.SASDLTWDNLK.G  R.FDEFFSEGCAPGSK.K  K.EGYYGYTGAFR.C  K.DYELLCLDGTR.K  R.KPVEEYANCHLAR.A | 1.34 |  |  |
| 597 | unnamed protein product, partial [*Homo sapiens*] similar to: MYH9 protein, partial [*Homo sapiens*] | MYH9 | BAF84298.1 | 117373/6.16 | 151 | 9 | 4 | 1571.9396  961.5535  1318.8161  924.5531 | 1570.8468  960.4777  1317.7405  923.4865 | 52  15  49  18 | K.VSHLLGINVTDFTR.G  R.NTNPNFVR.C  K.LDPHLVLDQLR.C  R.VVFQEFR.Q |  |  | 2.44 |
| 658 | protein disulfide-isomerase A4 isofrom 2 precursor [*Homo sapiens*] | PDIA4 | NP_004902 | 73229/4.96 | 197 | 16 | 3 | 1317.6470  1025.5685  879.4311 | 1316.6513  1024.5818  878.4399 | 69  46  18 | K.FHHTFSTEIAK.F  K.YALPLVGHR.K  R.AATQFWR.S |  | 8.60 | 9.41 |
| 682 | heat shock cognate 71 kDa protein isoform 2 [Homo sapiens] | HSPA8 | NP_694881 | 53598/5.62 | 234 | 18 | 4 | 1487.6953  1199.6654  1691.7116  1253.6117 | 1486.6940  1198.6670  1690.7183  1252.6088 | 46  23  72  37 | R.TTPSYVAFTDTER.L  K.DAGTIAGLNVLR.I  K.STAGDTHLGGEDFDNR.M  R.FEELNADLFR.G |  |  | 3.75 |
| 695 | heat shock cognate 71 kDa protein isoform X1 [*Homo sapiens*] | HSPA8 | XP_011541100.1 | 71082/5.37 | 221 | 29 | 2 | 1691.7015  1253.5998 | 1690.7183  1252.6088 | 88  49 | K.STAGDTHLGGEDFDNR.M  R.FEELNADLFR.G |  | 27.16 | 18.30 |
| 699 | unnamed protein product [*Homo sapiens*], highly similar to Homo sapiens heat shock 70kD protein 1A, mRNA | HSPA1A | BAF83984.1 | 70249/5.48/5.39 | 202 | 21 | 4 | 1197.6296  1261.5954  1417.6951  1315.5527  1110.5154 | 1196.6877  1260.6503  1416.7514  1314.5914  1109.5506 | 16  44  24  33 | K.DAGVIAGLNVLR.I  R.LVNHFVEEFK.R  R.LVNHFVEEFKR.K  R.FEELCSDLFR.S |  |  | 5.01 |
| 714 | HSPA1A [Homo sapiens] | HSPA1A | AQY76878.1 | 70280/5.48 | 408 | 32 | 3 | 1487.7010  1197.6488  1675.7381  1315.5761 | 1486.6940  1196.6877  1674.7234  1314.5914 | 98  31  85  76 | R.TTPSYVAFTDTER.L  K.DAGVIAGLNVLR.I  K.ATAGDTHLGGEDFDNR.L  R.FEELCSDLFR.S |  | 14.41 | 10.43 |
| 730 | endoplasmic reticulum chaperone BiP precursor [*Homo sapiens*] | HSPA5 | NP_005338.1 | 7402/5.07 | 436 | 35 | 6 | 2016.0635  1512.7564  1528.7537  1313.6302  1460.7726  1934.0195 | 2015.0589  1511.7442  1527.7391  1312.6122  1459.7518  1933.0058 | 35  28  14  61  78  91 | K.KVTHAVVTVPAYFNDAQR.Q  R.AKFEELNMDLFR.S  R.AKFEELNMDLFR.S + Oxidation (M)  K.FEELNMDLFR.S  K.SDIDEIVLVGGSTR.I  K.DNHLLGTFDLTGIPPAPR.G |  | 11.02 | 12.73 |
| 741 | keratin, type II cytoskeletal 8 isoform 1 [*Homo sapiens*] | KRT8 | NP_001243211.1 | 6573/5.37 | 218 | 32 | 2 | 1419.7440  1344.6798 | 1418.7405  1343.6681 | 69  42 | R.LEGLTDEINFLR.Q  R.ASLEAAIADAEQR.G |  | 8.15 | 7.52 |
| 752 | heat shock cognate 71 kDa protein isoform 1 [*Homo sapiens]* | HSPA8 | XP_011541100.1 | 71082/5.37 | 286 | 37 | 4 | 1669.8242  1199.6801  1253.6254  1319.6019  1304.6095 | 1668.8195  1198.6670  1252.6088  1318.5863  1303.6230 | 11  35  49  60  12 | K.HWPFMVVNDAGRPK.V + Oxidation (M)  K.DAGTIAGLNVLR.I  R.FEELNADLFR.G  K.NSLESYAFNMK.A + Oxidation (M)  K.CNEIINWLDK.N |  | 6.92 | 5.94 |
| 770 | ERO1-like protein alpha isoform 6 precursor [*Homo sapiens*] | ERO1A | NP_001369397.1 | 53670/5.69 | 685 | 41 | 11 | 2613.0197  1042.5155  2083.8612  2182.0537  1322.6818  1290.6256  1857.8114  1985.9096  2061.9759  2077.9691  1380.7169 | 2612.0288  1041.5131  2082.8800  2181.0928  1321.6918  1289.6252  1856.8370  1984.9319  2060.9989  2076.9939  1379.7197 | 47  33  71  16  42  77  63  119  37  13  62 | R.CFCQVSGYLDDCTCDVETIDR.F  K.LLESDYFR.Y  K.YSEEANNLIEECEQAER.L  K.IWNVIYEENCFKPQTIK.R  R.YLLQETWLEK.K  R.FDGILTEGEGPR.R  K.SFPLHFDENSFFAGDK.K  K.SFPLHFDENSFFAGDKK.E  K.LIANMPESGPSYEFHLTR.Q  K.LIANMPESGPSYEFHLTR.Q+Oxidation(M)  R.QEIVSLFNAFGR.I | 1.94 | 5.07 |  |
| 843 | T-complex protein 1 subunit theta isoform 1 [*Homo sapiens*] | CCT8 | NP_006576.2 | 60153/5.42 | 165 | 10 | 3 | 978.6008  1333.8704  1146.6952  1150.6983 | 977.5004  1332.7401  1145.5928  1149.5818 | 19  48  31  38 | K.APGFAQMLK.E + Oxidation (M)  K.LFVTNDAATILR.E  R.DIDEVSSLLR.T  K.FAEAFEAIPR.A |  |  | 3.82 |
| 855 | EH domain-containing protein 1 isoform 1 [*Homo sapiens*] | EHD1 | NP_001269373.1 | 60646/6.35 | 303 | 31 | 7 | 1169.6352  1342.6607  1358.6559  1236.6496  1482.7280  2046.0697 | 1168.6240  1341.6499 1357.6449 1235.6411 1481.7263 2045.0946 | 20  37  20  29  57  62 | K.LLPLEEHYR.F  R.HLIEQDFPGMR.I  R.HLIEQDFPGMR.I+Oxidation(M) K.LNAFGNAFLNR.F  R.EHQISPGDFPSLR.K K.LEGHELPADLPPHLVPPSK.R | 1.68 |  |  |
| 862 | keratin, type II cytoskeletal 5 *[Homo sapiens]* | KRT5 | NP_000415.2 | 62568/7.59 | 175 | 25 | 2 | 1203.6375  1890.9710  1093.5387  1194.5942 | 1202.6295 1889.9635 1092.5199 1193.5676 | 19  13  33  34 | K.WTLLQEQGTK.T  R.QNLEPLFEQYINNLR.R  K.AQYEEIANR.S  K.YEELQQTAGR.H | 1.46 |  |  |
| 891 | mitochondrial heat shock 60kD protein 1 variant 1 [*Homo sapiens*] | HSPD1 | ACE06961 | 60813/5.83 | 314 | 23 | 3 | 1344.7114  1919.0604  1684.8924 | 1343.7085  1918.0636  1683.8978 | 91  84  68 | R.TVIIEQSWGSPK.V  K.ISSIQSIVPALEIANAHR.K  R.AAVEEGIVLGGGCALLR.C |  | 15.54 | 11.64 |
| 892 | heterogeneous nuclear ribonucleoprotein K isoform a variant, partial [*Homo sapiens*] | HNRNPK | BAD92799.1 | 49002/5.48 | 159 | 13 | 2 | 1780.8348  1098.4897  1194.7179 | 1779.7911  1097.4448  1193.6921 | 48  21  63 | R.TDYNASVSVPDSSGPER.I  K.GSDFDCELR.L  R.NLPLPPPPPPR.G |  | 3.18 | 2.84 |
| 905 | heterogeneous nuclear ribonucleoprotein K isoform X2 [Homo sapiens] | HNRNPK | XP_016870157.1 | 51230/5.39 | 157 | 7 | 3 | 1780.8380  1098.4856  1194.7234 | 1779.7911  1097.4448  1193.6921 | 43  26  68 | R.TDYNASVSVPDSSGPER.I  K.GSDFDCELR.L  R.NLPLPPPPPPR.G |  |  | 2.93 |
| 908 | keratin, type I cytoskeletal 10 isoform 1 [*Homo sapiens*] | KRT10 | NP_000412.4 | 59020/5.13 | 155 | 21 | 2 | 1234.6644  1365.6267 | 1233.6717  1364.6320 | 31  50 | R.LKYENEVALR.Q  R.SQYEQLAEQNR.K |  | 4.23 | 3.34 |
| 920 | mitochondrial heat shock 60kD protein 1 variant 1 [*Homo sapiens*] | HSPD1 | ACE06961 | 60813/5.83 | 190 | 13 | 5 | 1344.7247  1919.1003  833.4314  960.5628  1684.9372 | 1343.7085  1918.0636  832.3828  959.5036  1683.8978 | 63  42  18  25  21 | R.TVIIEQSWGSPK.V  K.ISSIQSIVPALEIANAHR.K  K.APGFGDNR.K  R.VTDALNATR.A  R.AAVEEGIVLGGGCALLR.C |  | 9.35 | 8.22 |
| 968 | protein disulfide-isomerase A3 precursor [*Homo sapiens*] | PDIA3 | NP_005304.3 | 57146/5.98 | 468 | 32 | 10 | 1084.6556  877.5597  2575.4986  1172.6473  1359.7755  1515.8813  1664.8946  1680.8945  1341.7974  1370.8098 | 1083.5601  876.4817  2574.2966  1171.5332  1358.6507  1514.7518  1663.7512  1679.7461  1340.6765  1369.6878 | 11  17  34  35  49  72  27  62  64  49 | K.YGVSGYPTLK.I  K.LNFAVASR.K  K.TFSHELSDFGLESTAGEIPVVAIR.T  K.FVMQEEFSR.D  R.FLQDYFDGNLK.R  R.FLQDYFDGNLKR.Y  K.MDATANDVPSPYEVR.G  K.MDATANDVPSPYEVR.G + Oxidation (M)  R.GFPTIYFSPANK.K  R.ELSDFISYLQR.E |  | 3.55 | 3.68 |
| 977 | keratin, type II cytoskeletal 6A [*Homo sapiens*] | KRT6B | NP_005545 | 60293/8.09 | 318 | 27 | 5 | 1424.6409  1598.7538  1407.7150  1107.5487 | 1423.6263  1597.7519  1406.7041  1106.5356 | 35  68  94  27 | R.GSGGLGGACGGAGFGSR.S  R.ISIGGGSCAISGGYGSR.A  K.ADTLTDEINFLR.A  K.AQYEEIAQR.S |  | 23.03 | 11.24 |
| 979 | protein disulfide-isomerase precursor [*Homo sapiens*] | P4HB | NP_000909 | 57480/4.76 | 279 | 30 | 3 | 1081.6316  1833.9038  910.4254 | 1080.6695  1832.9057  909.4345 | 54  74  50 | K.THILLFLPK.S  K.ILFIFIDSDHTDNQR.I  K.FFPASADR.T |  | 25.16 | 23.93 |
| 990 | keratin, type II cytoskeletal 5 [Homo sapiens] | KRT5 | NP_000415.2 | 62568/5.79 | 239 | 35 | 2 | 1093.5423  1194.5929  1263.7126 | 1092.5199  1193.5676  1262.6870 | 27  38  67 | K.AQYEEIANR.S  K.YEELQQTAGR.H  K.LALDVEIATYR.K |  | 45.03 | 14.67 |
| 1006 | keratin type II, partial [*Homo sapiens*] | KRTL1 | AAA59466 | 60258/8.05 | 262 | 15 | 4 | 1424.6377  1598.7652  827.4371  1407.7123  1107.5460 | 1423.6263  1597.7519  826.4225  1406.7041  1106.5356 | 14  49  43  75  35 | R.GSGGLGGACGGAGFGSR.S  R.ISIGGGSCAISGGYGSR.A  K.FASFIDK.V  K.ADTLTDEINFLR.A  K.AQYEEIAQR.S |  | 49.47 | 12.97 |
| 1013 | tubulin alpha-1B chain [Homo sapiens] | TUBA1B | NP_006073.2 | 50804/4.94 | 442 | 37 | 5 | 2007.8464  1410.7527  1756.9242  1249.5229  1584.7280 | 2006.8858  1409.7667  1755.9559  1248.5453  1583.7443 | 130  64  69  11  60 | K.TIGGGDDSFNTFFSETGAGK.H  R.QLFHPEQLITGK.E  R.IHFPLATYAPVISAEK.A  K.YMACCLLYR.G  R.SIQFVDWCPTGFK.V |  | 7.28 | 5.94 |
| 1024 | keratin, type II cytoskeletal 5 [Homo sapiens] | KRT5 | NP_000415.2 | 62568/7.59 | 149 | 15 | 3 | 1410.7573  1093.5864  1194.6385 | 1409.6722  1092.5199  1193.5676 | 41  32  39 | R.VSLAGACGVGGYGSR.S  K.AQYEEIANR.S  K.YEELQQTAGR.H |  | 1.95 | 1.95 |
| 1042 | keratin, type II cytoskeletal 8 isoform 1 [Homo sapiens] | KRT8 | NP_001243211.1 | 56573/5.37 | 156 | 27 | 2 | 827.4817  1419.7420  1344.6801 | 826.4225  1418.7405  1343.6681 | 27  57  28 | K.FASFIDK.V  R.LEGLTDEINFLR.Q  R.ASLEAAIADAEQR.G |  | 3.37 | 5.42 |
| 1051 | keratin, type II cytoskeletal 8 isoform 1 [*Homo sapiens*] | KRT8 | NP_001243211.1 | 6573/5.37 | 218 | 32 | 2 | 1419.7440  1344.6798 | 1418.7405  1343.6681 | 69  42 | R.LEGLTDEINFLR.Q  R.ASLEAAIADAEQR.G |  | 7.74 | 10.66 |
| 1067 | tumor rejection antigen (gp96) 1 variant, partial [*Homo sapiens*] | HSP90B1 | BAD92771 | 66140/5.08 | 208 | 28 | 6 | 1785.9292  1081.5821  1529.8094  1187.7135  1015.5220  1485.7938 | 1784.8904  1080.5352  1528.7668  1186.6710  1014.4658  1484.7471 | 13  15  16  40  25  28 | R.EEEAIQLDGLNASQIR.E  K.FAFQAEVNR.M  K.NLLHVTDTGVGMTR.E + Oxidation (M)  K.SILFVPTSAPR.G  R.GLFDEYGSK.K  K.GVVDSDDLPLNVSR.E |  | 6.58 | 10.40 |
| 1075 | methanethiol oxidase isoform 3 [*Homo sapiens*] | SELENBP1 | NP_001245218.1 | 57457/6.04 | 172 | 23 | 4 | 1084.6478  1233.6294  1025.5818  1051.5400 | 1083.6652  1232.6401  1024.5917  1050.5498 | 23  38  18  35 | K.LVLPSLISSR.I  R.IYVVDVGSEPR.A  K.DGLIPLEIR.F  K.QFYPDLIR.E |  | -3.75 | -2.45 |
| 1114 | actin-related protein 3 isoform 2 [*Homo sapiens*] | ACTR3 | NP_001264069.1 | 42262/5.41 | 222 | 29 | 3 | 2444.5076  2482.5206  1214.7290  1297.8212  1003.5989 | 2443.1795  2481.1812  1213.5615  1296.6496  1002.4593 | 64  44  27  16 | K.GVDDLDFFIGDEAIEKPTYATK.W  R.AEPEDHYFLLTEPPLNTPENR.E  K.EFSIDVGYER.F  K.NIVLSGGSTMFR.D + Oxidation (M) |  |  | 1.46 |
| 1115 | ruvB-like 1 isoform X3 [*Homo sapiens*] | RUVBL1 | XP_005247898.1 | 44141/5.57 | 154 | 20 | 3 | 1687.9100  1948.9633  1708.7735 | 1686.9304 1947.9902 1707.7991 | 41  54  30 | R.ALESSIAPIVIFASNR.G R.GTEDITSPHGIPLDLLDR.V K.EHVEEISELFYDAK.S | -1.41 |  |  |
| 1140 | heterogeneous nuclear ribonucleoprotein H isoform i [*Homo sapiens*] | HNRPH1 | NP_001351179.1 | 43449/6.02 | 184 | 21 | 4 | 1684.7631  1092.5824 | 1683.7601  1091.5724 | 64  48 | K.HTGPNSPDTANDGFVR.L  R.VHIEIGPDGR.V |  | 1.55 | 1.65 |
| 1163 | glucose-6-phosphate 1-dehydrogenase isoform a [*Homo sapiens*] | G6PD | NP_000393.4 | 62942/8.23 | 209 | 18 | 5 | 1807.7954  1058.6403  1173.6450  1002.5642  1273.6416 | 1806.7809  1057.6284  1172.6302  1001.5447  1272.6139 | 38  22  15  11  51 | R.NSYVAGQYDDAASYQR.L  R.IIVEKPFGR.D  R.LSNHISSLFR.E  R.IFGPIWNR.D  R.GGYFDEFGIIR.D |  |  | 4.85 |
| 1167 | elongation factor 1-gamma [*Homo sapiens*] | EEF1G | NP_001395.1 | 50429/6.25 | 150 | 16 | 3 | 821.4771  1241.6761  1461.6822  1684.8029 | 820.4443  1240.6452  1460.6361  1683.7682 | 14  25  15  37 | R.TFLVGER.V  K.STFVLDEFKR.K  K.DGWSLWYSEYR.F  R.EYFSWEGAFQHVGK.A |  | 2.74 | 2.78 |
| 1184 | pyruvate kinase PKM isoform X4 [*Homo sapiens*] | PKM | XP_005254502.1 | 58470/7.96 | 265 | 32 | 7 | 1197.6409  1359.6937  953.4809  1837.8993  1019.5112  1931.9819  2175.0894 | 1196.6401 1358.6976 952.4726 1836.9040 1018.5083 1930.9788 2174.1107 | 40  30  16  15  33  18  27 | R.LDIDSPPITAR.N  R.NTGIICTIGPASR.S  K.IENHEGVR.R  R.RFDEILEASDGIMVAR.G + Oxidation (M) K.GDYPLEAVR.M  R.EAEAAIYHLQLFEELR.R R.LAPITSDPTEATAVGAVEASFK.C | -1.3 |  |  |
| 1196 | keratin, type I cytoskeletal 10 isoform 1 [*Homo sapiens*] | KRT10 | NP_000412 | 59020/5.13 | 164 | 22 | 2 | 807.3982  1365.6637 | 806.3923  1364.6320 | 24  60 | R.LAADDFR.L  R.SQYEQLAEQNR.K |  | -2.00 | -1.54 |
| 1232 | eukaryotic initiation factor 4AII [*Homo sapiens*] | EIF4A2 | NP_001958.2 | 46601/5.33 | 142 | 18 | 4 | 1544.8487  894.4513  910.4461  1114.6842 | 1543.8471  893.4429  909.4378  1113.6758 | 34  16  39  38 | K.LQAEAPHIVVGTPGR.V  R.VFDMLNR.R  R.VFDMLNR.R + Oxidation (M)  R.VLITTDLLAR.G |  | 2.77 | 2.86 |
| 1269 | heterogeneous nuclear ribonucleo protein F [*Homo sapiens*] | HNRPF | NP_001091676.1 | 45985/5.38 | 384 | 32 | 8 | 1709.7567  798.4543  1630.6889  1867.8901  1616.6604  1632.6569  1996.9308  1092.5588 | 1708.7792  797.4323  1629.7132 1866.9363 1615.6759 1631.6708  1995.9690 1091.5724 | 22  22  47  34  46  19  130  37 | R.QSGEAFVELGSEDDVK.M  R.YIEVFK.S  K.HSGPNSADSANDGFVR.L  K.ITGEAFVQFASQELAEK.A  R.DLSYCLSGMYDHR.Y  R.DLSYCLSGMYDHR.Y + Oxidation (M)  K.ATENDIYNFFSPLNPVR.V  R.VHIEIGPDGR.V | -1.28 |  |  |
| 1304 | keratin, type I cytoskeletal 17 [*Homo sapiens*] | KRT17 | NP_000413 | 48361/4.97 | 422 | 42 | 4 | 994.4187  807.3923  1222.6363  1272.5662  833.4234  1379.7147  1122.5583 | 993.4192  806.3923  1221.6353  1271.5822  832.4290  1378.7204  1121.5717 | 39  27  37  49  20  13  49 | R.DYSQYYR.T  R.LAADDFR.T  R.TKFETEQALR.L  R.KDAEDWFFSK.T  K.SEISELR.R  K.TRLEQEIATYR.R  R.LEQEIATYR.R |  | 80.65 | 53.96 |
| 1310 | unknown [*Homo sapiens*], similar to: Homo sapiens isocitrate dehydrogenase 1 (NADP+), soluble (IDH1) | IDH1 | AAX93221.1 | 46915/6.53 | 438 | 40 | 6 | 976.5523  1009.4433  1087.5559  1170.5589  1154.5212  1341.6684 | 975.5502  1008.4413  1086.5710  1169.5604  1153.5226  1340.6684 | 70  74  49  41  60  29 | R.NILGGTVFR.E  R.HAYGDQYR.A  R.ATDFVVPGPGK.V  K.DIFQEIYDK.Q  K.SEGGFIWACK.N  K.TVEAEAAHGTVTR.H |  | 5.20 | 5.56 |
| 1317 | alpha-enolase isoform 3 [*Homo sapiens*] | ENO1 | NP_001340275 | 47696/6.57 | 322 | 24 | 3? | 1804.9253  1425.7049  806.4220 | 1803.9366  1424.7187  805.4446 | 118  95  33 | R.AAVPSGASTGIYEALELR.D  R.YISPDQLADLYK.S  K.YNQLLR.I |  | 7.26 | 5.41 |
| 1399 | keratin, type I cytoskeletal 10 isoform 1 [*Homo sapiens*] | KRT10 | NP_000412.4 | 59020/5.13 | 167 | 22 | 2 | 807.4231  993.5080  1365.6838 | 806.3923  992.4927  1364.6320 | 24  11  50 | R.LAADDFR.L  K.YENEVALR.Q  R.SQYEQLAEQNR.K |  |  | 2.54 |
| 1407 | keratin, type I cytoskeletal 19 [*Homo sapiens*] | KRT19 | NP_002267.2 | 44079/5.04 | 889 | 67 | 7 | 1554.7602  1104.5570  1674.7865  1354.6139  1082.4992  1485.7173  1389.6918  1122.5771  1904.9050 | 1553.7434 1103.5393 1673.7685 1353.5983 1081.4829 1484.7042 1388.6783 1121.5717  1903.8911 | 130  15  117  69  65  43  36  33  94 | R.QSSATSSFGGLGGGSVR.F  K.LTMQNLNDR.L  R.DYSHYYTTIQDLR.D  R.SQYEVMAEQNR.K  K.DAEAWFTSR.T  R.EVAGHTEQLQMSR.S  K.AALEDTLAETEAR.F  R.LEQEIATYR.S  R.SLLEGQEDHYNNLSASK.V | -1.4 |  |  |
| 1426 | EF1a-like protein [*Homo sapiens*] | EEF1A1 | AAG44730 | 46901/7.12 | 152 | 11 | 2 | 1589.0465  1404.9050  1314.9037 | 1587.8733  1403.7197  1313.7343 | 32  92  15 | K.THINIVVIGHVDSGK.S  K.YYVTIIDAPGHR.D  R.EHALLAYTLGVK.Q |  | 12.71 |  |
| 1430 | PREDICTED: elongation factor 1-alpha 1 [*Homo sapiens*] | EEF1A1 | AAK93966.1 | 43282/8.94 | 90 | 12 | 2 | 1404.7065  1025.6031 | 1403.7197  1024.6030 | 50  15 | K.YYVTIIDAPGHR.D  K.IGGIGTVPVGR.V |  | 4.11 | 4.50 |
| 1435 | citrate synthase, mitochondrial precursor [*Homo sapiens*] | CS | NP_004068.2 | 51908/8.45 | 176 | 14 | 4 | 1762.8660  1338.6278  1167.6549 | 1761.8785  1337.6364  1166.6560 | 20  41  72 | K.GLVYETSVLDPDEGIR.F  R.DYIWNTLNSGR.V  R.VVPGYGHAVLR.K |  | 1.85 | 2.14 |
| 1453 | serpin H1 isoform X1 [*Homo sapiens*] | SERPINH1 | XP_024304524.1 | 46525/8.75 | 507 | 41 | 8 | 986.5273  1437.6839  1659.7488  1819.8108  1637.7191  1337.6844  1293.6369 | 985.5556 1436.7259 1658.7941 1818.8748 1636.7256 1336.7350 1292.6725 | 20  110  87  36  55  53  53 | K.AVLSAEQLR.D  R.DEEVHAGLGELLR.S  R.LYGPSSVSFADDFVR.S R.SALQSINEWAAQTTDGK.L  R.TGLYNYYDDEKEK.L  K.HLAGLGLTEAIDK.N  R.DTQSGSLLFIGR.L | 1.46 | 2.72 |  |
| 1465 | unnamed protein product [Homo sapiens]  highly similar to Homo sapiens serpin peptidase  inhibitor, clade B (ovalbumin), member 1 (SERPINB1), mRNA | SERPINB1 | BAF84016.1 | 42891/6.22 | 513 | 25 | 7 | 1602.8104  1305.7356  1686.9038  2052.9721  1218.6372  1785.9466  1510.7834 | 1601.7587 1304.6837 1685.8665 2051.9185 1217.5968 1784.8944 1509.7311 | 107  31  65  134  56  28  46 | K.TFHFNTVEEVHSR.F  R.FQSLNADINKR.G  K.TYNFLPEFLVSTQK.T K.TYGADLASVDFQHASEDAR.K  K.FAYGYIEDLK.C  R.FKLEESYTLNSDLAR.L  K.LEESYTLNSDLAR.L | -1.49 |  |  |
| 1502 | heterogeneous nuclear ribonucleoprotein H isoform x [*Homo sapiens*] | HNRNPH1 |  | 34108/5.14 | 173 | 24 | 4 | 1504.6992  1684.7772  1996.9672  1092.5823 | 1503.6776  1683.7601  1995.9690  1091.5724 | 22  44  32  31 | R.GLPWSCSADEVQR.F  K.HTGPNSPDTANDGFVR.L  R.ATENDIYNFFSPLNPVR.V  R.VHIEIGPDGR.V |  |  | 1.72 |
| 1546 | ribosomal protein SA pseudogene [*Homo sapiens*] | RPSA | NP_001342216.1 | 33002/4.78 | 218 | 33 | 3 | 1741.0131  1203.7022  2996.5848  1897.9265 | 1739.9417  1202.6408  2995.4709  1896.8505 | 95  61  10  11 | R.AIVAIENPADVSVISSR.N  K.FAAATGATPIAGR.F  R.ADHQPLTEASYVNLPTIALCNTDSPLR.Y  R.EHPWEVMPDLYFYR.D + Oxidation (M) |  | 1.81 | 2.06 |
| 1595 | inorganic pyrophosphatase [*Homo sapiens*] | PPA1 | NP_066952 | 33095/5.54 | 248 | 61 | 5 | 1053.5588 1863.9467  1327.7085  1114.6051  1694.9159 | 1052.5291 1862.9203 1326.6755 1113.5859 1693.8828 | 12  59  26  22  13 | R.AAPFSLEYR.V  K.GQYISPFHDIPIYADK.D  K.DVFHMVVEVPR.W  R.YVANLFPYK.G  R.LKPGYLEATVDWFR.R | -1.16 |  | 1.63 |
| 1611 | keratin, type I cytoskeletal 10 isoform 1 [*Homo sapiens*] | KRT10 | NP_000412.4 | 59020/5.13 | 158 | 35 | 3 | 1234.7860  1365.7632  1434.8977 | 1233.6717  1364.6320  1433.7626 | 22  33  21 | R.LKYENEVALR.Q  R.SQYEQLAEQNR.K  K.IRLENEIQTYR.S |  | 2.14 | 2.45 |
| 1612 | N-acetyl-D-glucosamine kinase isoform 1 [*Homo sapiens*] | NAGK | NP_060037.4 | 37694/5.81 | 255 | 42 | 4 | 1036.6411  1561.7860  1554.7534  1341.7119  1875.1127  1204.7002 | 1035.6077 1560.7380 1553.7085 1340.6684 1874.0666 203.6612 | 22  24  14  19  49  63 | K.AGVDPLVPLR.S  R.SLGLSLSGGDQEDAGR.I  K.QAMFHYFQVPDR.L + Oxidation (M)  K.IAEGAQQGDPLSR.Y  R.HIVAVLPEIDPVLFQGK.I  K.EGFLLALTQGR.E | -1.38 |  | 1.77 |
| 1615 | keratin, type I cytoskeletal 10 isoform [*Homo sapiens*] | KRT10 | NP_000412.4 | 59020/5.13 | 123 | 15 | 3 | 1707.7428  1365.6173  1493.7032 | 1706.7649  1364.6320  1492.7270 | 21  31  43 | K.GSLGGGFSSGGFSGGSFSR.G  R.SQYEQLAEQNR.K  R.SQYEQLAEQNRK.D |  |  | 4.10 |
| 1618 | F-actin-capping protein subunit alpha-1 [*Homo sapiens*] | CAPZA1 | NP_006126 | 33073/5.45 | 667 | 57 | 7 | 2088.9597  1197.6647  2140.9116  2245.0186  2314.0901  1570.8737  2028.9505 | 2088.0065 1196.6877 2139.9684    2244.0658 2313.1423 1569.9131 2028.0065 | 100  46  55  112  83  63  132 | K.FITHAPPGEFNEVFNDVR.L R.LLLNNDNLLR.E R.LLLNNDNLLR.E  R.EGAAHAFAQYNMDQFTPVK.I+Oxidation(M)  K.IEGYEDQVLITEHGDLGNSR.F K.TIDGQQTIIACIESHQFQPK.NK.FTITPPTAQVVGVLK.I K.IQVHYYEDGNVQLVSHK.D | -1.2 |  |  |
| 1722 | glyceraldehyde-3-phosphate dehydrogenase isoform 2 [*Homo sapiens*] | GAPDH | NP_001243728.1 | 31699/7.15 | 193 | 21 | 2 | 1530.8775  1763.8832 | 1529.7872  1762.7951 | 76  93 | R.VPTANVSVVDLTCR.L  K.LISWYDNEFGYSNR.V |  | 3.83 |  |
| 1749 | glyceraldehyde-3-phosphate dehydrogenase isoform 2 [*Homo sapiens*] | GAPDH | NP_001243728.1 | 31699/7.15 | 144 | 22 | 2 | 1530.8779  1763.8942 | 1529.7872  1762.7951 | 52  69 | R.VPTANVSVVDLTCR.L  K.LISWYDNEFGYSNR.V |  | 3.08 |  |
| 1756 | tryptase alpha/beta-1 precursor  [*Homo sapiens*] | TPSB2 | NP_003285.2 | 30952/6.62 | 143 | 20 | 4 | 1071.6015  1888.9781  908.5565  1366.6788 | 1070.5661  1887.9479  907.5531  1365.6313 | 17  53  12  32 | K.WPWQVSLR.V  R.EQHLYYQDQLLPVSR.I  R.LPPPFPLK.Q  K.YHLGAYTGDDVR.I |  | -2.23 |  |
| 1758 | alpha-soluble NSF attachment protein | NAPA | NP_003818.2 | 33667/5.23 | 638 | 64 | 10 | 1549.7468  1253.6114  1623.74491417.6901  1433.6980  1168.5937  1558.7382  1453.7864  1460.69472091.0020  1405.7641  1175.5883 | 1548.7209 1252.5757 1622.7148 1416.6489 1432.6439 1167.5594 1557.7099 1452.7612 1459.6619 2089.9804 1404.7435 1174.5466 | 51  49  72  15  14  18  72  42  79  41  12  12 | K.NSQSFFSGLFGGSSK.I  K.IEEACEIYAR.A  K.HDAATCFVDAGNAFK.K K.ADPQEAINCLMR.A  K.ADPQEAINCLMR.A + Oxidation (M) R.AIEIYTDMGR.F  K.AIAHYEQSADYYK.G  K.VAGYAALLEQYQK.A  K.YEELFPAFSDSR.E  K.LLEAHEEQNVDSYTESVK.E  R.LDQWLTTMLLR.I + Oxidation (M) K.TIQGDEEDLR.- | -1.32 |  |  |
| 1767 | glyceraldehyde-3-phosphate dehydrogenase [*Homo sapiens*] | GAPDH | NP_001243728.1 | 31699/7.15 | 261 | 31 | 3 | 811.5065  1530.9520  1763.9943 | 810.4058  1529.7872  1762.7951 | 17  101  113 | K.LTGMAFR.V + Oxidation (M)  R.VPTANVSVVDLTCR.L  K.LISWYDNEFGYSNR.V |  | 3.15 | 3.38 |
| 1775 | glyceraldehyde-3-phosphate dehydrogenase isoform 2 [*Homo sapiens*] | GAPDH | NP_001243728 | 31699/7.15 | 229 | 33 | 2 | 1530.7996  1763.8134 | 1529.7872  1762.7951 | 83  97 | R.VPTANVSVVDLTCR.L  K.LISWYDNEFGYSNR.V |  | 5.66 | 5.41 |
| 1782 | similar to Homo sapiens glyceraldehyde-3-phosphate dehydrogenase (GAPDH) mRNA with GenBank Accession Number M33197.1 [Homo sapiens] | GAPDH | AAG01996.1 | 36201/8.57 | 262 | 22 | 5 | 2229.2342  2245.2088  811.4885  1530.8809  1763.9103 | 2228.0970  2244.0919  810.4058  1529.7872  1762.7951 | 19  37  20  63  99 | R.VIISAPSADAPMFVMGVNHEK.Y + Oxidation (M)  R.VIISAPSADAPMFVMGVNHEK.Y + 2 Oxidation (M)  K.LTGMAFR.V + Oxidation (M)  R.VPTANVSVVDLTCR.L  K.LISWYDNEFGYSNR.V |  | 6.58 | 7.05 |
| 1785 | similar to Homo sapiens glyceraldehyde-3-phosphate dehydrogenase (GAPDH) mRNA with GenBank Accession Number M33197.1 [*Homo sapiens*] | GAPDH | AAG01996.1 | 36201/8.57 | 263 | 31 | 2 | 1530.9186  1763.9561 | 1529.7872  1762.7951 | 118  95 | R.VPTANVSVVDLTCR.L  K.LISWYDNEFGYSNR.V |  | 7.77 | 5.91 |
| 1820 | microtubule-associated protein RP/EB family member 1 isoform X1 [*Homo sapiens*] | MAPRE1 | XP_011526998.1 | 30151/5.02 | 304 | 36 | 5 | 1320.6832  1634.8111  1076.5195  2019.1633  1925.1003  2270.1184 | 1319.6510 1633.7565 1075.4611 2018.1160 1924.0490 2269.0644 | 54  11  29  69  19  38 | K.LEHEYIQNFK.I  K.FQDNFEFVQWFK.K  K.FFDANYDGK.D  R.QGQETAVAPSLVAPALNKPK.K  K.KPLTSSSAAPQRPISTQR.T R.NIELICQENEGENDPVLQR.I | -1.18 | 3.32 | 3.99 |
| 1870 | Cathepsin D preproprotein | CTSD | NP_001900.1 | 45037/6.10 | 179 | 25 | 3 | 963.4564  820.4510 | 962.4498  819.4239 | 41  30 | R.YYTVFDR.D  R.VGFAEAAR.L | -1.49 | -1.84 |  |
| 1877 | receptor of activated protein C kinase 1 [*Homo sapiens*] | GNB2L1 | NP_006089.1 | 35511/7.60 | 236 | 41 | 4 | 1192.6725  1264.7595  1309.7623  1366.7955 | 1191.5520  1263.6459  1308.6310  1365.6751 | 38  29  37  58 | R.DETNYGIPQR.A  R.LWDLTTGTTTR.R  K.DVLSVAFSSDNR.Q  R.YWLCAATGPSIK.I |  | 2.23 | 2.19 |
| 1902 | intracellular chloride channel protein [*Homo sapiens*] | CLIC1 | AQY77259.1 | 27119/5.17 | 208 | 27 | 4 | 1078.4688  1844.9024  1037.5021  957.4471 | 1077.5026  1843.9679  1036.5342  956.4715 | 15  63  38  35 | K.IGNCPFSQR.L  K.LAALNPESNTAGLDIFAK.F  R.GFTIPEAFR.G  R.YLSNAYAR.E |  | 1.84 | 2.02 |
| 1912 | lactate dehydrogenase A chain isoform 1 [*Homo sapiens*] | LDHA | NP_005557.1 | 36950/8.44 | 162 | 25 | 4 | 1649.8143  1665.7717  1248.6119  1118.5917 | 1648.7701  1664.7651  1247.5928  1117.5768 | 12  10  37  47 | K.GEMMDLQHGSLFLR.T + Oxidation (M)  K.GEMMDLQHGSLFLR.T + 2 Oxidation (M)  R.VIGSGCNLDSAR.F  K.SADTLWGIQK.E | 1.27 | 3.74 | 2.80 |
| 1915 | voltage-dependent anion-selective channel protein 1 isoform 1 [*Homo sapiens*] | VDAC1 | NP_001387950.1 | 30868/8.62 | 239 | 39 | 5 | 854.5443  1374.7491  1400.7569  1213.6914 | 853.4698  1373.6503  1399.6620  1212.6139 | 31  14  51  55 | K.GYGFGLIK.L  R.WTEYGLTFTEK.W  K.LTFDSSFSPNTGK.K  R.VTQSNFAVGYK.T |  | 2.11 | 1.69 |
| 1918 | L-lactate dehydrogenase A chain isoform 1 [*Homo sapiens*] | LDHA | NP_005557.1 | 36950/8.44 | 177 | 36 | 2 | 913.5306  929.5236  1118.5224 | 912.5756  928.5746  1117.5768 | 36  14  28 | K.LVIITAGAR.Q  K.FIIPNVVK.Y  K.SADTLWGIQK.E |  | 4.04 | 3.46 |
| 1921 | porin isoform 1 [Homo sapiens] | VDAC1 | NP_001387950.1 | 30868/8.62 | 205 | 35 | 2 | 854.4933  1374.6885  1400.7038  2176.0707  1213.6319  2103.2170 | 853.4698  1373.6503  1399.6620  2174.9725  1212.6139  2102.1735 | 15  20  23  12  32  37 | K.GYGFGLIK.L  R.WTEYGLTFTEK.W  K.LTFDSSFSPNTGK.K  R.EHINLGCDMDFDIAGPSIR.G + Oxidation (M)  R.VTQSNFAVGYK.T  K.VNNSSLIGLGYTQTLKPGIK.L |  | 2.48 | 2.43 |
| 1922 | porin isoform 1 [Homo sapiens] | VDAC1 | NP_001387950.1 | 30868/8.62 | 196 | 28 | 3 | 854.5275  1374.7316  1400.7452  1213.6833 | 853.4698  1373.6503  1399.6620  1212.6139 | 17  43  61  48 | K.GYGFGLIK.L  R.WTEYGLTFTEK.W  K.LTFDSSFSPNTGK.K  R.VTQSNFAVGYK.T |  | 2.40 | 2.01 |
| 1928 | 14-3-3 protein epsilon [*Homo sapiens*] | YWHAE | NP_006752 | 29326/4.63 | 203 | 38 | 2 | 1256.5919  1189.6699 | 1255.5833  1188.6536 | 81  61 | R.YLAEFATGNDR.K  K.DSTLIMQLLR.D |  | 3.71 | 2.99 |
| 1950 | unnamed protein product [Homo sapiens], similar to: heat shock protein 27 [*Homo sapiens*] | HSPB1 | BAG34835.1 | 22826/5.98 | 379 | 41 | 5 | 960.4647  1163.6525  1783.9429  1104.5343  1906.0225 | 959.4250  1162.6135  1782.9152  1103.4996  1904.9843 | 28  66  103  32  61 | R.DWYPHSR.L  R.LFDQAFGLPR.L  R.VSLDVNHFAPDELTVK.T  R.QDEHGYISR.C  K.LATQSNEITIPVTFESR.A |  |  | 1.52 |
| 1964 | carbonic anhydrase 1 isoform a [*Homo sapiens*] | CA1 | NP_001158302.1 | 28909/6.59 | 350 | 28 | 3 | 1928.9464  985.4128  1612.7384 | 1928.0003  984.4301  1611.7794 | 141  66  96 | K.HDTSLKPISVSYNPATAK.E  K.GGPFSDSYR.L  K.YSAELHVAHWNSAK.Y |  | -3.32 | -5.26 |
| 2015 | 14-3-3 protein beta/alpha [*Homo sapiens*] | YWHAB | XP_016883528.1 | 28179/4.76 | 166 | 29 | 4 | 932.4380  1252.6560  1108.5741  1124.5693  1189.6863  1205.6789 | 931.4222  1251.6394  1107.5495  1123.5444  1188.6536  1204.6485 | 11  33  28  17  51  47 | K.MKGDYFR.Y + Oxidation (M)  K.KEMQPTHPIR.L + Oxidation (M)  K.EMQPTHPIR.L  K.EMQPTHPIR.L + Oxidation (M)  K.DSTLIMQLLR.D  K.DSTLIMQLLR.D + Oxidation (M) |  | 3.43 | 3.51 |
| 2018 | 14-3-3 protein zeta/delta isoform X1 [*Homo sapiens*] | YWHAZ | XP_024303034.1 | 27899/4.73 | 112 | 24 | 3 | 1108.5758  1189.6803  1205.6770 | 1107.5495  1188.6536  1204.6485 | 27  51  33 | K.EMQPTHPIR.L  K.DSTLIMQLLR.D  K.DSTLIMQLLR.D + Oxidation (M) |  | 6.89 | 4.33 |
| 2026 | albumin, partial [*Homo sapiens*] | ALB | AEE60908.1 | 68484/5.73 | 120 | 13 | 2 | 960.5982  1358.6970 | 959.5552  1357.6224 | 41  37 | K.FQNALLVR.Y  K.AVMDDFAAFVEK.C + Oxidation (M) |  | -2.57 | -2.57 |
| 2027 | actin, cytoplasmic 2 [*Homo sapiens*] | ACTB | NP_001186883.1 | 42108/5.31 | 262 | 24 | 4 | 1132.5555  1790.9122  2231.1007  795.4659  1516.7356 | 1131.5197  1789.8846  2230.0576  794.4650  1515.6954 | 27  92  10  16  63 | R.GYSFTTTAER.E  K.SYELPDGQVITIGNER.F  K.DLYANTVLSGGTTMYPGIADR.M + Oxidation (M)  K.IIAPPER.K  K.QEYDESGPSIVHR.K |  | 1.74 | 2.86 |
| 2059 | similar to Homo sapiens phosphoglycerate mutase (PGAM-B) mRNA with GenBank Accession Number J04173.1 [*Homo sapiens*] | PGAM1 | AAG01990.1 | 28900/6.67 | 203 | 35 | 3 | 1312.6161  2433.1528  1150.6837 | 1311.5956  2432.0995  1149.6618 | 66  45  11 | R.HGESAWNLENR.F  R.SYDVPPPPMEPDHPFYSNISK.D + Oxidation (M)  R.VLIAAHGNSLR.G |  | 1.50 | 1.64 |
| 2062 | peroxiredoxin-4 precursor [*Homo sapiens*] | PRDX4 | 3TKS_A | 28132/6.10 | 500 | 67 | 8 | 2443.1232  2185.9640  797.4967  1464.8661  1624.8022  920.5347  1225.7252  1212.6593  2403.2153 | 2442.0659 2184.9171 796.4555  1463.8348  1623.7529  919.5015  1224.6826  1211.618242402.1689 | 18  27  37  44  98  22  51  50  39 | R.TREEECHFYAGGQVYPGEASR.V R.EEECHFYAGGQVYPGEASR.V  R.QGGLGPIR.I  R.IPLLSDLTHQISK.D  K.DYGVYLEDSGHTLR.G  R.GLFIIDDK.G  R.QITLNDLPVGR.S  R.LVQAFQYTDK.H K.HGEVCPAGWKPGSETIIPDPAGK.L | -1.23 |  | 3.55 |
| 2067 | carbonic anhydrase 2 isoform 1 [*Homo sapiens*] | CA2 | NP_000058.1 | 29285/6.87 | 227 | 47 | 4 | 2140.1188  935.4744  1581.8330  1169.5374 | 2139.0848  934.4509  1580.8099  1168.5149 | 40  29  53  43 | K.YDPSLKPLSVSYDQATSLR.I  K.GGPLDGTYR.L  K.YAAELHLVHWNTK.Y  K.SADFTNFDPR.G |  | -4.50 | -4.97 |
| 2151 | mutant hemoglobin subunit alpha 2 [*Homo sapiens*] | HBA1 | AXY55000.1 | 1577/7.96 | 192 | 58 | 2 | 1529.8186  1071.6189 | 1528.7270  1070.5471 | 92  36 | K.VGAHAGEYGAEALER.M  R.MFLSFPTTK.T |  | 2.54 | 2.61 |
| 2218 | apolipoprotein A-I isoform 1 preproprotein [*Homo sapiens*] | APOA1 | NP_001304947.1 | 30759/5.56 | 321 | 59 | 3 | 1612.7944  1283.5861  1386.7294 | 1611.7781  1282.5652  1385.7078 | 126  25  72 | K.LLDNWDSVTSTFSK.L  K.WQEEMELYR.Q  K.VSFLSALEEYTK.K |  |  | -2.76 |
| 2253 | glutathione S-transferase P[*Homo sapiens*] | GSTP1 | NP_000843.1 | 23569/5.43 | 216 | 31 | 4 | 1484.7787  1337.7538  1136.5929  751.4331  2155.0888 | 1483.7534  1336.7180  1135.5696  750.4276  2154.0521 | 12  72  36  22  29 | -.MPPYTVVYFPVR.G + Oxidation (M)  M.PPYTVVYFPVR.G  K.ASCLYGQLPK.F  R.TLGLYGK.D  K.YISLIYTNYEAGKDDYVK.A |  | 2.57 | 2.51 |
| 2270 | superoxide dismutase [Mn], mitochondrial isoform C precursor [*Homo sapiens*] | SOD2 | NP_001309744.1 | 18364/8.48 | 134 | 33 | 2 | 1738.9869  1744.0195 | 1737.8434  1742.8740 | 84  15 | K.HHAAYVNNLNVTEEK.Y  K.AIWNVINWENVTER.Y |  | 2.46 | 2.89 |
| 2418 | cyclophilin B [*Homo sapiens*] | PPIB | NP_000933.1 | 23785/9.42 | 206 | 35 | 4 | 812.4254  880.5231  1286.5981  1490.6962 | 811.4228  879.5218  1285.5761  1489.6772 | 30  41  30  29 | K.VYFDLR.I  R.VIFGLFGK.T  K.DFMIQGGDFTR.G  K.HYGPGWVSMANAGK.D + Oxidation (M) | -1.28 | 2.66 | 3.46 |
| 2444 | nucleoside diphosphate kinase A isoform a [*Homo sapiens*] | NME1 | NP_937818 | 19869/5.42 | 134 | 27 | 2 | 1344.7726  1149.6556 | 1343.7561  1148.6342 | 45  43 | R.TFIAIKPDGVQR.G  K.DRPFFAGLVK.Y |  | 3.17 | 3.24 |
| 2446 | cyclophilin B [*Homo sapiens*] | PPIB | NP_000933.1 | 23785/9.42 | 345 | 33 | 6 | 880.5407  1286.5942  896.4329  1490.6963  1031.5732 | 879.5218 1285.5761  895.4076 1489.6772  1030.5481 | 29  73  42  72  29 | R.VIFGLFGK.T  K.DFMIQGGDFTR.G  R.FPDENFK.L  K.HYGPGWVSMANAGK.D + Oxidation (M) K.VLEGMEVVR.K | -1.35 | 2.51 | 3.37 |
| 2570 | AF162690_1 transthyretin precursor [*Homo sapiens*] | TTR | AAD45014.1 | 15991/5.52 | 367 | 64 | 5 | 1366.7556  1522.7097  1394.6160  2451.1857 | 1365.7517  1521.7100  1393.6150  2450.1979 | 82  66  85  11 | R.GSPAINVAVHVFR.K  R.KAADDTWEPFASGK.T  K.AADDTWEPFASGK.T  K.ALGISPFHEHAEVVFTANDSGPR.R |  |  | -2.52 |
| 2695 | myosin light polypeptide 6 isoform 1 [*Homo sapiens*] | MYL6 | NP_066299.2 | 17090/4.56 | 160 | 38 | 3 | 1025.5486  1357.6765  1544.7473 | 1024.4978  1356.6166  1543.6791 | 41  17  55 | K.EAFQLFDR.T  K.ILYSQCGDVMR.A + Oxidation (M)  K.DQGTYEDYVEGLR.V |  |  | 1.71 |
| 2706 | protein S100-A9 [*Homo sapiens*] | S100A9 | NP_002956 | 13291/5.71 | 293 | 68 | 3 | 1806.9843  1455.7624  971.5108 | 1805.9312  1454.7154  970.4872 | 66  109  47 | R.NIETIINTFHQYSVK.L  K.LGHPDTLNQGEFK.E  R.LTWASHEK.M |  |  | -1.85 |
| 2777 | hemoglobin subunit delta [*Homo sapiens*] | HBD | NP_000510.1 | 16159/7.85 | 242 | 53 | 2 | 2060.9205  1669.8915  1126.5612 | 2059.9197  1668.8835  1125.5567 | 77  55  39 | R.FFESFGDLSSPDAVMGNPK.V + Oxidation (M)  K.VLGAFSDGLAHLDNLK.G  K.LHVDPENFR.L |  | -2.23 | -3.98 |
| 2791 | mutant hemoglobin subunit alpha 2 | HBA1 | AXY55000.1 | 15177/7.96 | 239 | 58 | 3 | 1071.5396  1087.5510  1833.8743 | 1070.5471  1086.5420  1832.8846 | 63  50  119 | R.MFLSFPTTK.T  R.MFLSFPTTK.T + Oxidation (M)  K.TYFPHFDLSHGSAQVK.G |  | -2.57 | -4.61 |
| 2797 | delta-globin B2 variant, partial [*Homo sapiens*] | HBD | ACC69182.1 | 11651/6.49 | 257 | 72 | 2 | 2060.9193  1669.8928  1126.5665 | 2059.9197  1668.8835  1125.5567 | 80  65  39 | R.FFESFGDLSSPDAVMGNPK.V + Oxidation (M)  K.VLGAFSDGLAHLDNLK.G  K.LHVDPENFR.- |  | -2.38 | -4.33 |
| 2857 | unnamed protein product [*Homo sapiens*] highly similar to Homo sapiens S100 calcium binding protein A11 (calgizzarin) (S100A11), mRNA | S100A11 | BAG35117.1 | 11744/6.56 | 100 | 21 | 2 | 1849.9943  1866.0019 | 1848.8968  1864.8917 | 65  80 | K.TEFLSFMNTELAAFTK.N  K.TEFLSFMNTELAAFTK.N + Oxidation (M) |  | 3.44 | 3.84 |
| 2917 | beta globin, partial [*Homo sapiens*] | HBB | ACF16788 | 11550/6.17 | 507 | 93 | 2 | 932.5221  2074.9655  1669.9007  1126.5715  1140.5882 | 931.5127  2073.9354  1668.8835  1125.5567  1139.5723 | 65  148  119  53  55 | K.SAVTALWGK.V  R.FFESFGDLSTPDAVMGNPK.V + Oxidation (M)  K.VLGAFSDGLAHLDNLK.G  K.LHVDPENFR.-  K.LHVEPENFR.- |  | -6.42 | -6.48 |
| 2920 | mutant hemoglobin subunit alpha 1 [*Homo sapiens*] | HBA1 | AXY55000.1 | 15177/7.96 | 228 | 38 | 3 | 1071.5576  1087.5705  1833.8902 | 1070.5471  1086.5420  1832.8846 | 59  49  122 | R.MFLSFPTTK.T  R.MFLSFPTTK.T + Oxidation (M)  K.TYFPHFDLSHGSAQVK.G |  | -2.88 | -3.20 |
| 2921 | mutant hemoglobin subunit alpha 2 [*Homo sapiens*] | HBA2 | AXY55000.1 | 15177/7.96 | 246 | 58 | 3 | 1071.5496  1087.5700  1833.8864 | 1070.5471  1086.5420  1832.8846 | 66  60  125 | R.MFLSFPTTK.T  R.MFLSFPTTK.T + Oxidation (M)  K.TYFPHFDLSHGSAQVK.G |  | -6.72 | -6.54 |
| 2929 | mutant hemoglobin subunit alpha 2 [*Homo sapiens]* | HBA2 | AXY55000.1 | 15177/7.96 | 220 | 38 | 2 | 1071.5374  1087.5909  1833.8737 | 1070.5471  1086.5420  1832.8846 | 59  47  124 | R.MFLSFPTTK.T  R.MFLSFPTTK.T + Oxidation (M)  K.TYFPHFDLSHGSAQVK.G |  | -7.29 | -6.09 |
| 3064 | Cofilin-1 | CFL1 | NP_005498.1 | 18719/8.22 | 319 | 35 | 2 | 1309.8306  2166.3440  1337.7898  1990.2898 | 1308.6748  2165.0892  1336.6187  1989.0611 | 56  86  101  38 | K.AVLFCLSEDKK.N  K.EILVGDVGQTVDDPYATFVK.M  R.YALYDATYETK.E  K.KEDLVFIFWAPESAPLK.S |  | 2.62 | 2.81 |
| 3065 | 14-3-3 protein sigma [*Homo sapiens*] | SFN | CAG46703 | 27871/4.68 | 143 | 27 | 3 | 1070.5306  1189.6759  1205.6615 | 1069.5226  1188.6536  1204.6485 | 21  51  32 | K.EMPPTNPIR.L + Oxidation (M)  K.DSTLIMQLLR.D  K.DSTLIMQLLR.D + Oxidation (M) |  | 6.27 | 3.88 |
| 3066 | calreticulin precursor [*Homo sapiens*] | CARL | NP_004334.1 | 48283/4.29 | 404 | 25 | 4 | 1410.6802  1607.7970  1147.7244  1019.6273 | 1409.6212  1606.7667  1146.6550  1018.5600 | 101  83  60  57 | K.EQFLDGDGWTSR.W  R.FYALSASFEPFSNK.G  K.KVHVIFNYK.G  K.VHVIFNYK.G |  | 6.80 | 8.55 |
| 3067 | beta globin, partial [*Homo sapiens*] | HBB | ULD54883.1 | 11553/6.49 | 281 | 77 | 2 | 1669.8864  1126.5648 | 1668.8835  1125.5567 | 137  59 | K.VLGAFSDGLAHLDNLK.G  K.LHVDPENFR.- |  | -2.22 | -4.15 |
| 3068 | phosphoglycerate kinase 1 [*Homo sapiens*] | PGK1 | NP_000282 | 44985/8.30 | 267 | 30 | 3 | 1634.7805  2023.0191  809.3884 | 1633.7849  2022.0310  808.4079 | 111  81  22 | K.LGDVYVNDAFGTAHR.A  K.ITLPVDFVTADKFDENAK.T  K.YAEAVTR.A |  | 4.14 | 4.79 |
| 3069 | ezrin [*Homo sapiens*] | EZR | NP_001104547.1 | 69484/5.94 | 326 | 22 | 3 | 894.5590  1104.6038  1310.7047  1182.6167 | 893.5375  1103.5764  1309.6819  1181.5869 | 17  49  87  83 | K.LFFLQVK.E  K.IGFPWSEIR.N  K.KAPDFVFYAPR.L  K.APDFVFYAPR.L |  | -2.01 | -4.07 |
| 3093 | rho GDP-dissociation inhibitor 2 [Homo sapiens] | ARHGDIB | XP_024304747.1 | 23031/5.10 | 148 | 11 | 3 | 1084.6166  855.5080  966.4841 | 1083.6077  854.4974  965.4719 | 32  26  52 | K.LNYKPPPQK.S  K.APNVVVTR.L  K.YVQHTYR.T | -1.51 |  |  |
| 3070 | hemoglobin subunit alpha [*Homo sapiens*] | HBA2 | NP_000508.1 | 15305/8.72 | 528 | 64 | 2 | 1529.8388  1071.6224  1087.6426  1833.9974  2996.6983  3012.6569 | 1528.7270  1070.5471  1086.5420  1832.8846  2995.4821  3011.4771 | 107  38  28  139  163  168 | K.VGAHAGEYGAEALER.M  R.MFLSFPTTK.T  R.MFLSFPTTK.T + Oxidation (M)  K.TYFPHFDLSHGSAQVK.G  K.VADALTNAVAHVDDMPNALSALSDLHAHK.L  K.VADALTNAVAHVDDMPNALSALSDLHAHK.L + Oxidation (M) |  | -2.30 | -4.11 |
| 3071 | fructose-bisphosphate aldolase A isoform 1 [*Homo sapiens]* | ALDOA | NP_001121089.1 | 39851/8.30 | 176 | 43 | 2 | 1342.7113  1647.1062 | 1341.7041  1645.8019 | 68  30 | K.ADDGRPFPQVIK.S  R.LQSIGTENTEENRR.F |  | 4.51 | 4.51 |
| 3073 | hemoglobin subunit beta [*Homo sapiens*] | HBB | QBH68937.1 | 16072/7.12 | 357 | 65 | 2 | 2074.9361  1669.8845  1126.5591 | 2073.9354  1668.8835  1125.5567 | 107  109  47 | R.FFESFGDLSTPDAVMGNPK.V + Oxidation (M)  K.VLGAFSDGLAHLDNLK.G  K.LHVDPENFR.L |  | -4.19 | -6.52 |
| 3074 | peptidyl-prolyl cis-trans isomerase A isoform 1 [*Homo sapiens*] | PPIA | NP_066953 | 18229/7.68 | 308 | 55 | 4 | 1379.7651  1598.7696  1831.9344  1154.5866  848.4434 | 1378.7496  1597.7381  1830.9039  1153.5655  847.4076 | 55  43  94  43  23 | R.VSFELFADKVPK.T  R.IIPGFMCQGGDFTR.H  K.SIYGEKFEDENFILK.H  K.FEDENFILK.H  K.TEWLDGK.H |  |  | 2.10 |
