# Supplementary material for: Application of two-dimensional difference gel electrophoresis to identify protein changes between center, margin, and adjacent non-tumor tissues obtained from non-small-cell lung cancer with adenocarcinoma or squamous cell carcinoma subtype
Source: PLoS One. 2022 May 5;17(5):e0268073. doi: 10.1371/journal.pone.0268073 (PMC9071164; doi:10.1371/journal.pone.0268073)
Supplement: S3 Table — (DOCX) [file pone.0268073.s003.docx]

S3 Table. Mixing and dying scheme of ADC lung cancer samples, n=8 for each group.

| **Gel no** | **Cy2** | **Cy3** | **Cy5** |
| --- | --- | --- | --- |
| 1 | Pooled Std. | Control 1 | Margin 5 |
| 2 | Pooled Std. | Center 1 | Control 5 |
| 3 | Pooled Std. | Margin 1 | Center 5 |
| 4 | Pooled Std. | Control 2 | Margin 6 |
| 5 | Pooled Std. | Center 2 | Control 6 |
| 6 | Pooled Std. | Margin 2 | Center 6 |
| 7 | Pooled Std. | Control 3 | Margin 7 |
| 8 | Pooled Std. | Center 3 | Control 7 |
| 9 | Pooled Std. | Margin 3 | Center 7 |
| 10 | Pooled Std. | Control 4 | Margin 8 |
| 11 | Pooled Std. | Center 4 | Control 8 |
| 12 | Pooled Std. | Margin 4 | Center 8 |

Control (Control 1-8), center (Center 1-8) and margin (Margin 1-8) tissue collected from individual patients
