# Supplementary material for: Application of two-dimensional difference gel electrophoresis to identify protein changes between center, margin, and adjacent non-tumor tissues obtained from non-small-cell lung cancer with adenocarcinoma or squamous cell carcinoma subtype
Source: PLoS One. 2022 May 5;17(5):e0268073. doi: 10.1371/journal.pone.0268073 (PMC9071164; doi:10.1371/journal.pone.0268073)
Supplement: S9 Material — (DOCX) [file pone.0268073.s013.docx]

1. **Summary of Standard Operating Procedure regulating the process of tissue samples collection in the clinical setting – macroscopic evaluation of resected specimen**

During the surgical resection of lung cancer, Study Nurses from Biobank are present in the surgical room since the beginning of this procedure to record the warm ischemia time (including time of clamping of particular vessels), record the time of cold ischemia, collect final tissue samples and transport them to the Pathology Department and Biobank. After lung tumor resection, whole specimen is examined macroscopically by the pathologist to determine the exact tumor localization, presence or absence of macroscopic residual tumor, presence or absence of macroscopic infiltration of pulmonary pleura, macroscopic evaluation of possible presence of necrosis in the tumor center. Pathologist cut the exact tissue samples that represent the tumor center and tumor margin. Moreover, pathologist determine the possibility to collect adjacent pulmonary tissue (referred as “control tissue”) – if the distance from the tumor border is greater than 2 centimeters, pathologist is cutting the samples of adjacent tissue. Then, Study Nurses from Biobank (who were present in the surgery room during the resection procedure to record time of vessels clamping, warm ischemia time, and cold ischemia time), are putting the tissue samples cut by pathologist alternately into labelled cryotubes for vapour phase of liquid nitrogen (fresh frozen samples) and into labelled tubes with 10% buffered formalin (formalin-fixed samples). The tissue samples are preserved according to the following pattern: first tissue sample collected from the tumor center is preserved in cryotube in liquid nitrogen, second tissue sample collected from the tumor center is preserved in buffered formalin and so on until all samples are preserved. The same pattern is used for the tissue samples cut from the tumor margin and tissue samples cut from adjacent tissue.

1. **Summary of Standard Operating Procedure regulating the process of microscopic evaluation of fresh frozen and formalin-fixed paraffin-embedded (FFPE) tissue samples.**

Quality control and microscopic evaluation are performed for fresh frozen tissue samples and the corresponding FFPE samples. The whole procedure of microscopic quality control of H&E-stained tissue sections prepared from fresh frozen tissue samples and FFPE samples is performed by a pathologist, and tissue section preparation is done by the laboratory staff from the Pathology Department. The preparation of frozen sections are performed from tissues frozen in liquid nitrogen in a cryostat, where cryo-tissue sections are cut at 5 μm. An uninterrupted cold chain during removal and processing of tissue is maintained to ensure consistent tissue quality. After mounting the tissue section on the superfrost pluss glass slide, standardized hematoxylin and eosin (H&E) staining is performed.

The semi-automatic rotation microtome is used for the FFPE sample cuts. The cuts are routinely performed at 4 μm into mounting slide and then the H&E staining procedure is carried out.

The microscopic quality assessment of H&E-stained tissue section includes following attributes:
- Confirmation of the specified organ,

- Histopathological diagnosis compatible with the present H&E section,

- Percentage of tumor content: the epithelial part of the tumor (no tumor stroma),

- Percentage of necrosis (%),

- Severity of presence of acellular substance,

- Severity of inflammation,

- Severity of fibrosis,

- Severity of hemorrhage.

Following tissue characteristics: acellular substance, inflammation, fibrosis, and hemorrhage are classified according to severity based on a number system (low – 1, medium – 2, high – 3).

For adjacent normal tissue samples (referred as “control tissue”), pathologist is ensuring that they contain a representative proportion of organ-specific epithelium or tissue. Normal tissue samples that contain a tumor cells are evaluated as a tumor sample depending on tumor content and tissue quality.

Criteria used to distinguish the tumor margin during microscopic evaluation by pathologist include determination of invasive margin and borderline between normal and tumor tissue. Every tissue sample collected from tumor center and tumor margin defined so in the macroscopic evaluation after tumor resection, are examined in the context of intratumoral morphological heterogeneity. Since the width of the peritumoral area is not defined clearly in official standards, in this study and for routine examination of biobanked samples, the tumor margin is characterized within 1 µm.
